# Management of Tooth Extraction in Patients Taking Antiresorptive Drugs: An Evidence Mapping Review and Meta-Analysis

**DOI:** 10.3390/jcm12010239

**Published:** 2022-12-28

**Authors:** Chang Liu, Yu-Tao Xiong, Tao Zhu, Wei Liu, Wei Tang, Wei Zeng

**Affiliations:** State Key Laboratory of Oral Diseases, National Clinical Research Centre for Oral Diseases, Department of Oral and Maxillofacial Surgery, West China Hospital of Stomatology, Sichuan University, Chengdu 610041, China

**Keywords:** medication-related osteonecrosis of the jaw, tooth extraction, preventive dentistry, evidence mapping, meta-analysis

## Abstract

Background: Medication-related osteonecrosis of the jaw (MRONJ) is a well-known severe adverse reaction of antiresorptive, antiangiogenic or targeted therapies, and usually occurs after tooth extraction. This review is aimed at determining the efficacy of any intervention of tooth extraction to reduce the risk of MRONJ in patients taking antiresorptive drugs, and present the distribution of evidence in these clinical questions. Methods: Primary studies and reviews were searched from nine databases (Medline, EMBase, Cochrane Library, Scopus, WOSCC, Inspec, KCI-KJD, SciELO and GIM) and two registers (ICTRP and ClinicalTrials.gov) to 30 November 2022. The risk of bias was assessed with the ROBIS tool in reviews, and the RoB 2 tool and ROBINS-I tool in primary studies. Data were extracted and then a meta-analysis was undertaken between primary studies where appropriate. Results: Fifteen primary studies and five reviews were included in this evidence mapping. One review was at low risk of bias, and one randomized controlled trial was at moderate risk, while the other eighteen studies were at high, serious or critical risk. Results of syntheses: (1) there was no significant risk difference found between drug holiday and drug continuation except for a subgroup in which drug continuation was supported in the reduced incidence proportion of MRONJ for over a 3-month follow-up; (2) the efficacy of the application of autologous platelet concentrates in tooth extraction was uncertain; (3) there was no significant difference found between different surgical techniques in any subgroup analysis; and (4) the risk difference with antibacterial prophylaxis versus control was −0.57, 95% CI −0.85 to −0.29. Conclusions: There is limited evidence to demonstrate that a drug holiday is unnecessary (and may in fact be potentially harmful) in dental practice. Primary closure and antibacterial prophylaxis are recommended despite limited evidences. All evidence have been graded as either of a low or very low quality, and thus further high-quality randomized controlled trials are needed to answer this clinical question.

## 1. Introduction

Medication-related osteonecrosis of the jaw (MRONJ) is a well-known severe adverse reaction of antiresorptive, antiangiogenic or targeted therapies, usually known as bisphosphonate-related osteonecrosis of the jaw (BRONJ) or antiresorptive agent-related osteonecrosis of the jaw (ARONJ), because most MRONJ occurred related to antiresorptive drugs, such as bisphosphonates (BP) and denosumab (Dmab). The American Association of Oral and Maxillofacial Surgeons (AAOMS) defines MRONJ as having the following three characteristics: (1) current or previous treatment with antiresorptive therapy alone or in combination with immune modulators or antiangiogenic medications; (2) exposed bone or bone that can be probed through an intra- or extra-oral fistula(e) in the maxillofacial region that has persisted for more than 8 weeks; and (3) no history of radiation therapy to the jaws or metastatic disease to the jaws [1].

Antiresorptive drugs are widely used in the treatment of primary osteoporosis and glucocorticoid-induced osteoporosis (secondary to rheumatoid arthritis, systemic lupus erythematosus and other autoimmune diseases) in a low-dose oral route, and in the treatment of Paget’s disease and malignant tumors (e.g., multiple myeloma, bone metastases) in a high-dose route. Antiangiogenic or targeted drugs are usually applied in patients with cancer. A list of medications with a potential to cause MRONJ have been collected systematically from published literature [2,3,4,5,6], and are shown in Table 1 and Table 2.

Oral surgeries, especially tooth extractions, are confirmed as one of the vital risk factors of MRONJ [1,7], which means that patients taking antiresorptive or antiangiogenic drugs have a higher risk of delayed healing of wounds after extraction, and a higher risk for the wound to develop into MRONJ. The best current estimate for the risk of MRONJ after extraction is 0.5% in patients exposed to oral BP, and 1.6 to 14.8% to intravenous BP [8]. However, tooth extraction is sometimes necessary and unavoidable for patients suffering toothache due to serious caries or periodontitis. Hence, how to reduce the risk of MRONJ when extracting teeth from patients taking antiresorptive drugs, immune modulators or antiangiogenic drugs is an important question.

Most current studies about MRONJ are focused not prevention but treatment or risk factors [9,10,11,12,13,14]; few of the current studies are aimed at prevention of MRONJ for tooth extraction [15,16], but have included as many study designs (such as case series) as possible, which could have degraded the certainty of the evidence. Therefore, this evidence mapping review is aimed at determining the efficacy of any intervention of tooth extraction to reduce the risk of MRONJ in patients taking antiresorptive drugs, immune modulators or antiangiogenic drugs, and to present the distribution of evidence in these clinical questions.

## 2. Materials and Methods

A protocol was registered online in the PROSPERO (ID: CRD42021287246) [17], and the protocol and this evidence mapping review followed the PRISMA 2020 statement [18] and the Cochrane Handbook for Systematic Reviews of Interventions [19].

### 2.1. Eligibility Criteria

Inclusion criteria were in the PICOS framework as follows:

1. Participants/population: patients taking antiresorptive drugs, immune modulators, or antiangiogenic drugs who needed tooth extractions.
2. Intervention/exposure: tooth extraction with any unlimited intervention.
3. Comparator/control: tooth extraction with any unlimited comparator, including blank control and placebo control.
4. Outcomes: primary outcomes were the prevalence or incidence of MRONJ or the delayed healing of extracted sockets, all-cause mortality (crude death rate) and MRONJ-related mortality (death rate with MRONJ); secondary outcomes were complications after tooth extraction (such as pain, swelling, and skeletal-related events), and quality of life (QoL) after tooth extraction.
5. Study design: (1) primary controlled studies, including randomized controlled trials (RCTs) and nonrandomized controlled studies (NRSs), such as historical controlled trials and cohort studies; (2) secondary studies, including systematic reviews and scoping reviews; and (3) tertiary studies, including umbrella reviews (overviews of reviews) and meta-epidemiological studies.

Exclusion criteria were (1) non-controlled studies, such as case reports, case series, cross-sectional studies, and one-armed cohort studies; (2) reports without eligible outcomes (i.e., abstracts or protocols published only); (3) ongoing studies; (4) reports focused on other clinical questions; and (5) nonclinical studies, such as in vitro studies and animal studies.

### 2.2. Search Methods

The following nine databases were searched for both published and unpublished papers to 30 November 2022: Medline via Ovid, EMBase via Ovid, the Cochrane Library, Scopus, Web of Science Core Collection (WOSCC), Inspec, Korean Science Citation Index-Korean Journal Database (KCI-KJD), Science Electronic Library Online (SciELO), and Global Index Medicus (GIM). Moreover, two register platforms were searched for registered clinical trials to 30 November 2022 as well: the International Clinical Trials Registry Platform (ICTRP), and ClinicalTrials.gov. All search strategies are available in Supplementary Materials. There were no restrictions on language or publication date. Furthermore, a cited reference search was conducted based on the included studies.

### 2.3. Selection and Data Collection of Studies

Two reviewers screened the title and abstracts of each record retrieved on EndNote Desktop independently, and then obtained the full reports for all studies that appeared to meet the inclusion criteria. All of the full reports retrieved were assessed by the two reviewers independently to verify whether to include or exclude them, and any disagreements were resolved either by discussion or by the involvement of another reviewer as an arbiter. Two reviewers extracted data from included studies independently and resolved their disagreements by discussion or the involvement of another reviewer as an arbiter. 

### 2.4. Assessment of Risk of Bias in Included Studies

The risk of bias in the included studies was assessed independently by two reviewers, and any disagreements were resolved by discussion or the involvement of another reviewer as an arbiter. The risk of bias in reviews was assessed with the ROBIS tool (Risk of Bias in Systematic Reviews) [20]. The risk of bias in RCTs was assessed with RoB 2 tool (Revised Tool for Risk of Bias in Randomized Trials) [21]. The risk of bias in NRSs was assessed with a ROBINS-I tool (Risk of Bias in Nonrandomized Studies of Interventions) [22]. The effect of assignment to intervention was taken into consideration for all included primary studies. 

### 2.5. Effect Measures

For dichotomous outcomes, the effect estimate was calculated as a risk difference (RD) with a 95% confidence interval (CI) and a risk ratio (RR) with 95% CI would be reported if appropriate. For continuous outcomes, mean values and standard deviations (SDs) have been used to express the estimate of effect as a mean difference (MD) with 95% CI.

### 2.6. Data Synthesis Methods

A meta-analysis was undertaken with a random-effect model on Review Manager only when primary studies of similar comparisons reported the same outcomes, leading to a more conservative interpretation. Clinical heterogeneity was described as characteristics; statistical heterogeneity was assessed using the chi-squared test at a significance level of 0.10, and the I^2^ statistic ranged from 0% to 100%. If there were sufficient studies and heterogeneity, subgroup analyses would be taken into consideration, including types of interventions, types of study designs (randomized or nonrandomized), length of follow-up, characteristics of participants, or diagnostic criteria of the outcome. An intention-to-treat (ITT) analysis and a modified intention-to-treat (mITT) analysis were used in the studies with participants withdrawn or switching interventions, which was recommend to reduce performance bias and attrition bias [21,22]. Forest plots were present as the results of data synthesis.

### 2.7. Assessment of Publication Bias

If there had been more than ten studies in the same meta-analysis of any comparison, the publication bias would have been assessed by visually inspecting a funnel plot for asymmetry. 

### 2.8. Assessment of Certainty

The quality of the evidence was assessed as high, moderate, low, or very low by two reviewers independently in accordance with GRADE criteria [23] for study design, risk of bias, consistency, directness and precision of results, and reporting bias. Any disagreements were resolved by discussion or the involvement of another reviewer as an arbiter.

## 3. Results

### 3.1. Selection of Studies

**Figure 1:**
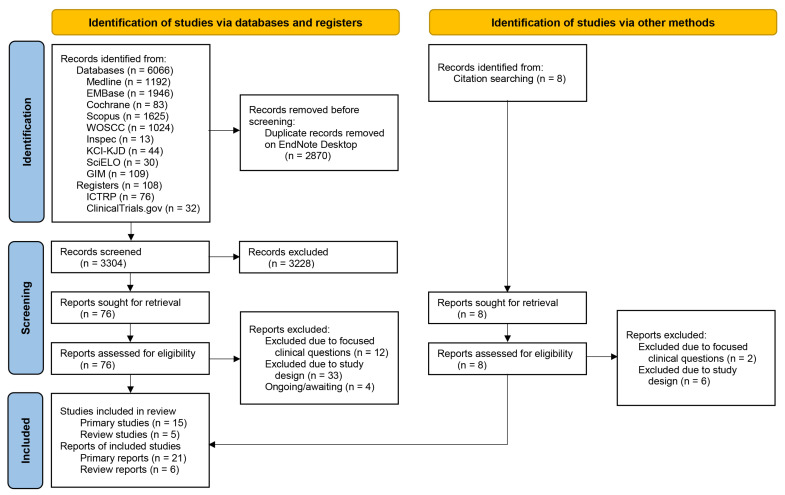
PRISMA 2020 flow diagram for this evidence mapping review.

As shown in Figure 1, a total of 6174 records were retrieved from nine databases and two registers, and 3304 records were screened by two reviewers after 3228 duplicates were removed. In addition to eight records from the cited reference search, a total of 84 full reports was retrieved and assessed for eligibility. A total of 15 primary studies (21 primary reports), and five review studies (six review reports) were included, while 57 reports were excluded; 39 reports were non-controlled studies (one-armed studies), four were studies ongoing or awaiting classification, and the other 14 were excluded due to focused clinical questions. Details about search strategies and the selection process of studies, and lists of excluded studies with references and reasons for exclusion are available in Supplementary Materials.

### 3.2. Evidence Map, Characteristics and Risk-of-Bias of Included Studies

**Figure 2:**
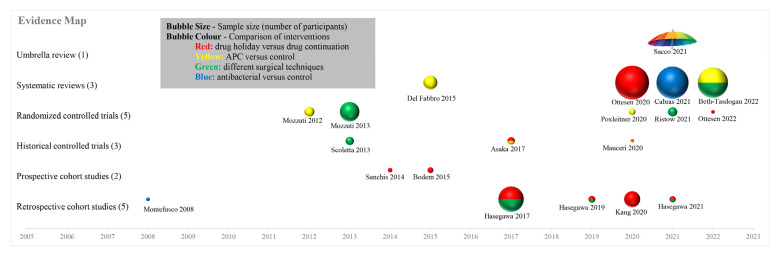
Evidence map (temporal profile) [15,16,24,25,26,27,28,29,30,31,32,33,34,35,36,37,38,39,40].

The distribution of different publication years, different study designs and different comparisons of all the twenty included studies were shown in an evidence map (Figure 2).

#### 3.2.1. Characteristics of Included Secondary and Tertiary Studies

Of all the five included review studies, there was one umbrella review (Sacco 2021 [24]) as a tertiary study, and four systematic reviews (Beth-Tasdogan 2022 [25,41], Cabras 2021 [16], Del Fabbro 2015 [26] and Ottesen 2020 [15]) as secondary studies. Characteristics of the included reviews are presented briefly in Table 3, and details are available in the Supplementary Materials.

#### 3.2.2. Characteristics of Included Primary Studies

Of all the fifteen included primary studies, there were five RCTs: Mozzati 2012 [27,42], Mozzati 2013 [28,43], Ottesen 2022 [29,44], Poxleitner 2020 [30] and Ristow 2021 [31,45]); three historical controlled trials (Asaka 2017 [32], Mauceri 2020 [33] and Scoletta 2013 [34,46]); two prospective cohort studies (Bodem 2015 [35] and Sanchis 2014 [36]); and five retrospective cohort studies (Hasegawa 2017 [37,47], Hasegawa 2019 [38], Hasegawa 2021 [39], Kang 2020 [40] and Montefusco 2008 [48]).

**Figure 3:**
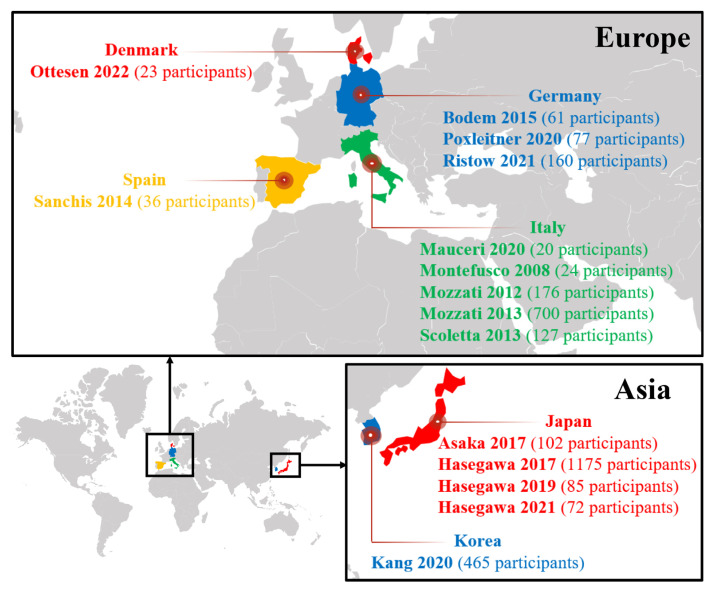
Evidence map of primary studies (regional profile) [27,28,29,30,31,32,33,34,35,36,37,38,39,40].

Ten of the fifteen primary studies were set in Europe; five in Italy; three in Germany; one in Denmark; and one in Spain. The other five were set in Asia: four in Japan and one in Korea (Figure 3). 

Characteristics of the included primary studies are briefly presented in Table 4, and details are available in the Supplementary Materials. Clinical and methodological heterogeneity were found regarding drug type, clinical indication, study design, follow-up duration, type of intervention, etc., which are described as characteristics in Table 4 and in the Supplementary Materials.

#### 3.2.3. Risk of Bias in Included Studies

For reviews, Beth-Tasdogan 2022 [25,41] was judged as low risk of bias while the other four reviews [15,16,24,26] were judged as high risk.

For primary studies, four RCTs were judged as having a high risk of overall bias, one RCT was judged as having some concerns (moderate risk), four NRSs were judged as having serious risk, and one NRS was judged as having a critical risk.

**Figure 4:**
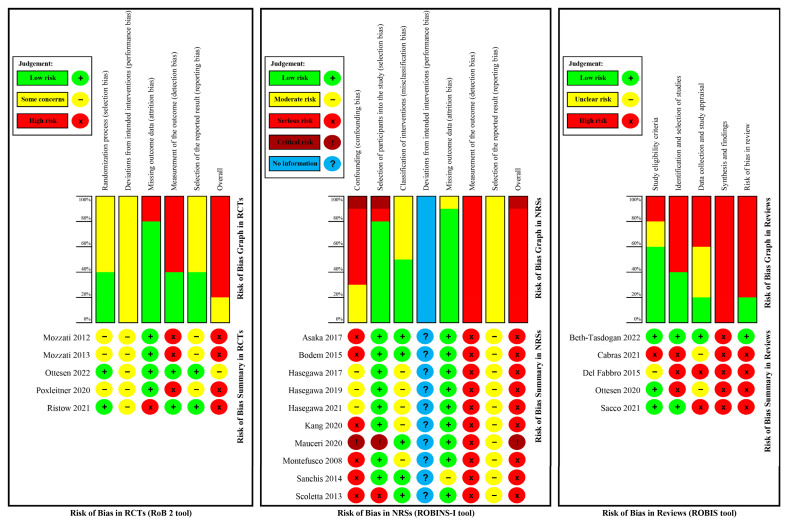
Risk of bias assessment in this evidence mapping review [15,16,24,25,26,27,28,29,30,31,32,33,34,35,36,37,38,39,40].

The risk of bias graph and summary in all included studies was shown in Figure 4. Responses to signalling questions and descriptions for judgements with the ROBIS tool, ROBINS-I tool and RoB 2 tool are available in the Supplementary Materials.

### 3.3. Results of Syntheses

Comparisons of interventions in all included studies were:

- Drug holiday versus drug continuation (nine primary studies: Asaka 2017 [32], Bodem 2015 [35], Hasegawa 2017 [37], Hasegawa 2019 [38], Hasegawa 2021 [39], Kang 2020 [40], Mauceri 2020 [33], Ottesen 2022 [29], and Sanchis 2014 [36]; two reviews: Ottesen 2020 [15] and Sacco 2021 [24]);
- Autologous platelet concentrates (APC) versus control, including platelet-rich fibrin (PRF), plasma rich in growth factors (PRGF) and platelet-rich plasma (PRP) (four primary studies: Asaka 2017 [32], Mauceri 2020 [33], Mozzati 2012 [27] and Poxleitner 2020 [30]; three reviews: Beth-Tasdogan 2022 [25], Del Fabbro 2015 [26] and Sacco 2021 [24]);
- Comparisons of different surgical techniques (six primary studies: Hasegawa 2017 [37], Hasegawa 2019 [38], Hasegawa 2021 [39], Mozzati 2013 [28], Ristow 2021 [31] and Scoletta 2013 [34]; one review: Beth-Tasdogan 2022 [25]);
- Antibacterial prophylaxis versus control (one primary study: Montefusco 2008 [48]; two reviews: Cabras 2021 [16] and Sacco 2021 [24]).

Primary outcomes were reported in all included primary studies, while only two studies (Ottesen 2022 [29] and Poxleitner 2020 [30]) reported secondary outcomes. An assessment of publication bias was unnecessary because there were not sufficient studies in each synthesis.

#### 3.3.1. Comparison 1: Drug Holiday versus Drug Continuation

Ottesen 2020 [15] and Sacco 2021 [24] reported that the efficacy of a drug holiday was uncertain, without quantitative analysis.

In the drug holiday groups from primary studies, there was a drug suspended for 3 months before extraction in Asaka 2017 [32], for an average of 17.6 months before extraction in Bodem 2015 [35], for over 2 months before extraction in Hasegawa 2017 [37] and Hasegawa 2019 [38], for over 1 month before extraction in Hasegawa 2021 [39], for an average of 7 months before extraction in Mauceri 2020 [33], for 1 month before extraction and 3 months after extraction in Ottesen 2022 [29], and for an average of 5.6 months before extraction in Sanchis 2014 [36].

**Figure 5:**
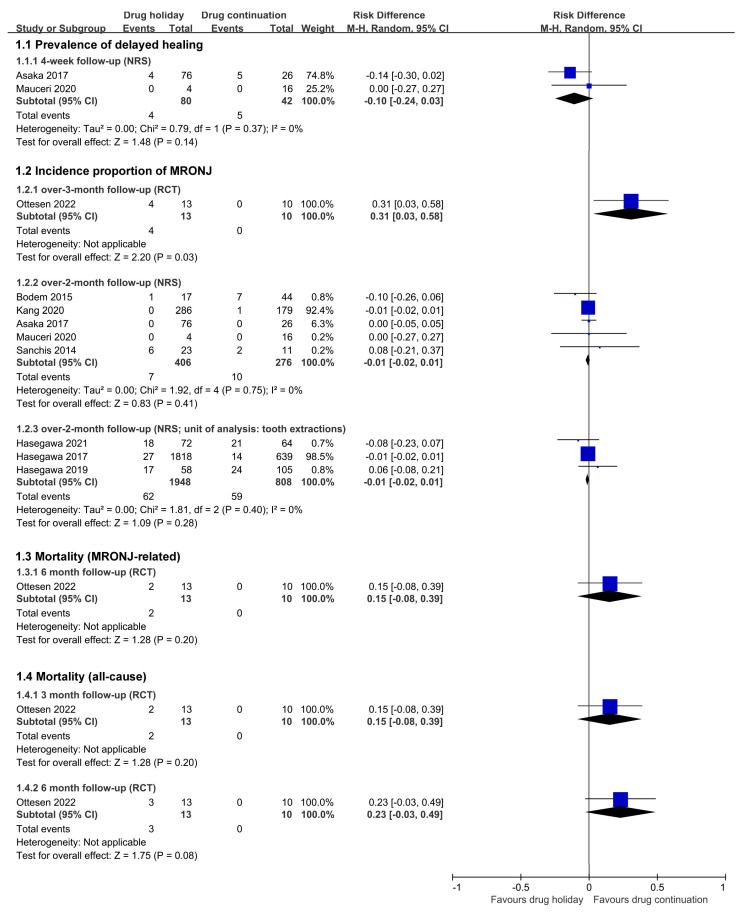
Forest plot of Comparison 1 (drug holiday versus drug continuation) [29,32,33,35,36,37,38,39,40].

Summary and syntheses measured with risk differences of primary outcomes are shown in Figure 5. There was no statistical heterogeneity found within subgroups. There was no significant risk difference found in any subgroup analysis except for subgroup 1.2.1., in which drug continuation was supported in the reduced incidence proportion of MRONJ in the case of over 3-month follow-up from one RCT (Ottesen 2022 [29]). 

Secondary outcomes were reported in Ottesen 2022 [29]: (1) Complications after extraction: there were four skeletal-related events (one fracture and three skeletal slight pains) from the 13 participants in the drug holiday group and two skeletal-related events (one fracture and one skeletal slight pain) from the 10 participants in the drug continuation group during the 6-month follow-up (RR 1.54, 95% CI 0.35 to 6.78, *p* = 0.57; RD 0.11, 95% CI −0.25 to 0.46, *p* = 0.55); progression of malignant tumours was observed in three participants from the drug holiday group and none from the drug continuation group during the 6-month follow-up (RD 0.23, 95% CI −0.03 to 0.43, *p* = 0.08). (2) QoL after extraction: statistical significance was found at 1-month follow-up in the EuroQoL-5D-5L results (*p* = 0.025), and the EuroQoL-Visual Analog Scale results showed that four participants who had developed MRONJ in the drug holiday group demonstrated great variation.

#### 3.3.2. Comparison 2: APC versus Control

**Figure 6:**
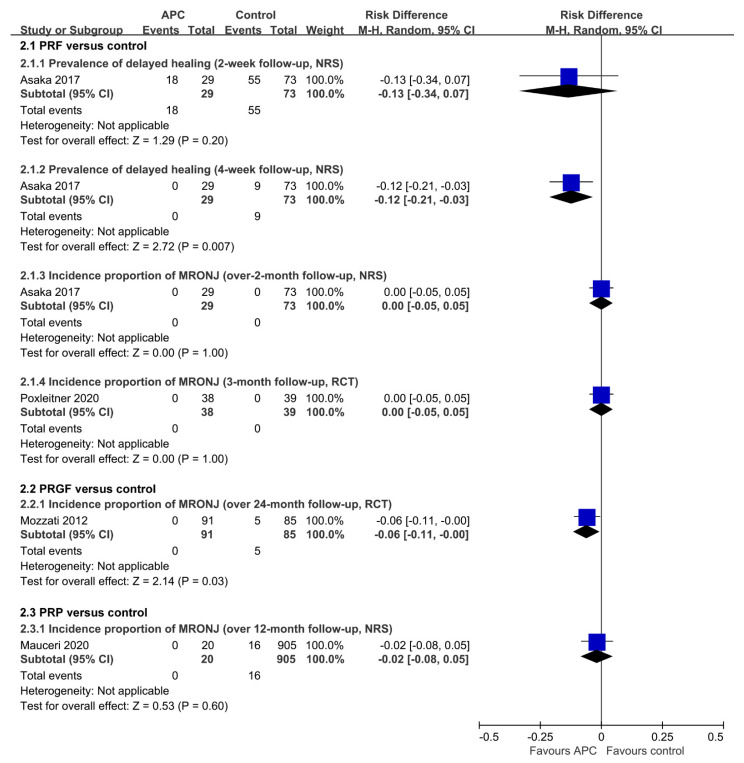
Forest plot of Comparison 2 (APC versus control) [27,30,32,33].

Comparison 2.1 (PRF versus control): Summary of primary outcomes measured with risk differences was shown in Figure 6. For prevalence of delayed healing of extracted sockets in Asaka 2017 [32], RD of PRF over control was −0.12, 95% CI −0.21 to −0.03 at 4-week follow-up (*p* = 0.007), while there was no significant difference found in the other subgroup analyses. Another study (Poxleitner 2020 [30]) reported that complications after extraction had been observed in one patient from 38 participants in the PRF group, and six patients from 39 participants in the control group (*p* = 0.108); however, there were no details about the complications.

Comparison 2.2 (PRGF versus control): The studies of Sacco 2021 [24] and Beth-Tasdogan 2022 [25] were based on only one RCT (Mozzati 2012 [27]), which compared the RRGF group with the control group, and the RD of PRGF for the incidence of MRONJ was −0.06, 95% CI −0.11 to −0.00 during a follow-up over 24 months (*p* = 0.03).

Comparison 2.3 (PRP versus control): The PRP group and the control group were compared in only one study (Mauceri 2020 [33]), and there was no statistical significance.

#### 3.3.3. Comparison 3: Different Surgical Techniques

A comparison of different surgical techniques was shown in Table 5. There was no significant difference found in any subgroup analysis. (Forest plots are shown in the Supplementary Materials).

#### 3.3.4. Comparison 4: Antibacterial Prophylaxis versus Control

An antibacterial prophylaxis was recommended in two reviews (Sacco 2021 [24] and Cabras 2021 [16]), the evidence of which was based on non-controlled studies. Moreover, antibacterial prophylaxis was reported or recommended in ten included primary studies (Table 6). However, only one retrospective cohort study (Montefusco 2008 [48]) reported a comparison of antibacterial prophylaxis versus control: no one developed MRONJ from 10 participants after tooth extraction with antibacterial prophylaxis, while eight developed it out of 14 participants without antibacterial prophylaxis (RD −0.57, 95% CI −0.85 to −0.29, *p* < 0.0001; RR 0.08, 95% CI 0.01 to 1.25, *p* = 0.07).

### 3.4. Certainty of Evidence

All studies were graded as either low or very low due to very serious imprecision and very serious risk of bias. Summary of findings tables are available in the Supplementary Materials.

## 4. Discussion

### 4.1. Interventions of Tooth Extraction in Patients at Risk of MRONJ

#### 4.1.1. Drug Holiday

In 2014, AAOMS [8] recommended a 2-month drug holiday before an invasive dental procedure to be a prudent approach for those patients with extended bisphosphonate-exposure histories (>4 years), although there had been limited data to support or refute the benefits of a drug holiday. The AAOMS proposal was supported as a position paper by the Japanese Allied Committee on Osteonecrosis of the Jaw [49] and the Korean Association of Oral and Maxillofacial Surgeons [50]. In 2017, a drug holiday was not recommended by the Scottish Dental Clinical Effectiveness Programme because the benefits of taking the drugs to manage the patients’ medical condition were likely to outweigh the small risk of developing MRONJ and, in the case of the bisphosphonates or denosumab, stopping the drug did not eliminate the risk of developing MRONJ [51]. In the last 2022 update of the AAOMS position paper [1], a drug holiday was thought to be controversial, based on Ottesen 2020 [15].

In this review, there were no significant benefits of a drug holiday found from nine included primary studies and the systematic review (Ottesen 2020 [15]), which included three prospective and 11 retrospective studies, and concluded that the efficacy of a high-dose antiresorptive drug holiday remained uncertain because of different results from different retrospective studies, while only one controlled prospective study (Bodem 2015 [35]) included in Ottesen’s review [15] indicated that the holiday would not reduce the risk of MRONJ and therefore must be seen as unnecessary. Moreover, a retrospective study of 5639 elderly patients with osteoporosis in Japan stated that waiting for over 2 months before extraction was a risk factor for delayed healing of longer than 8 weeks (OR 7.23, 95% CI 2.19 to 23.85, *p* = 0.001) [52]. Another retrospective cohort study of 81,427 elderly women in the U.S. reported that the adjusted hazard ratio (HR) for hip fractures of women who had discontinued alendronate for >2 years over those who had continued therapy was 1.3, 95% CI 1.1 to 1.4, adjusted HR for humerus fracture, 1.3, 95% CI 1.1 to 1.66, and adjusted HR for clinical vertebral fracture, 1.2, 95% CI 1.1 to 1.4, and that results were similar for risedronate, zoledronate and ibandronate for hip and clinical vertebral fracture [53]. Considering that incidence rate of MRONJ ranged from 8.2 to 12.8 per million person-years in the bisphosphonate-exposure population [54], the incidence proportion from 0.5% to 14.8% [8], and prevalence from 5% to 19% [55], it could be concluded that a drug holiday would increase the negative effects such as increased fracture occurrence and progression of malignant tumours, which would outweigh the risk of MRONJ [29,51].

#### 4.1.2. APC

In this review, the efficacy of three different APCs was reported from four included primary studies: two studies of PRF (Asaka 2017 [32] and Poxleitner 2020 [30]), one of PRGF (Mozzati 2012 [27]), and one of PRP (Mauceri 2020 [33]). However, the efficacy of APC to prevent MRONJ in most studies turned out to be not statistically different, in accordance with the findings of Del Fabbro (2015) [26]. However, when it came to the treatment of MRONJ, the efficacy of APC would be significant [26]. From limited evidence, it was still uncertain whether APC could prevent patients with tooth extractions from MRONJ.

#### 4.1.3. Surgical Techniques

There were insufficient controlled studies to support or refute the benefits of a primary closure (healing by primary intention) or a flap design to prevent patients from MRONJ. A primary closure was still recommended as a prudent approach for tooth extraction in patients at risk of MRONJ or who were suffering from MRONJ [1,8,49].

#### 4.1.4. Antibacterial Prophylaxis

Standard antibiotic schedules were undertaken in ten primary studies (Table 6): β-lactams were preferred antibiotics, such as amoxicillin, penicillin and sultamicillin. AAOMS [8] and KAOMS [50] recommended the use of antibiotics among patients with MRONJ at Stage 0, Stage 2 or Stage 3, despite the fact that the efficacy of antibiotics to reduce the risk of MRONJ was still uncertain because of a lack of controlled clinical studies.

### 4.2. Limitations

#### 4.2.1. Internal Validity of Included Primary Studies

In this review, internal validity was degraded due to the serious risk of bias within the included studies.

One of the five RCTs (Ottesen 2022 [29]) was judged as having some concerns, while the other four were at high risk of overall bias; three (Mozzati 2012 [27], Mozzati 2013 [28], and Poxleitner 2020 [30]) were at high risk of detection bias because of no blinding of outcome assessment, and one (Ristow 2021 [31]) was at high risk of attrition bias due to 28 withdrawals (17.5%) from 160 participants. Detection bias is a common bias during the measurement process, consisting of outcome assessors’ directed errors when aware of the intervention received, and inherent systematic error of measurement methods lacking sensitivity. Hence, the risk of detection bias will be lower if both a blinding of the outcome assessment and a more sensitive and more specific measurement method are applied [21,22].

There is a serious risk of detection bias in all ten NRSs due to no blinding of the outcome assessment. Confounding factors which could have biased the causal inferences due to the causal relationship from confounding factors to outcomes should have been controlled by appropriate analyses in NRSs and perfect randomization in RCTs [21,22]. However, in Asaka 2017 [32], Bodem 2015 [35], Kang 2020 [40], Montefusco 2008 [48], Sanchis 2014 [36] and Scoletta 2013, either not all confounding factors had been taken into consideration in regression analysis, or there was no control for confounding bias. In a historical controlled trial (Mauceri 2020 [33]), the participants in the retrospective group were all selected from a literature search, which had caused a critical risk of selection bias and confounding bias. Another historical controlled trial (Scoletta 2013 [34]) was at serious risk of selection bias due to the inclusion of a follow-up of at least 4 months. Thus, Mauceri (2020) was at critical risk of overall bias and the other nine were all at serious risk of overall bias.

#### 4.2.2. External Validity of Included Primary Studies

Remarkable clinical heterogeneity was found in primary studies concerning drug type, dose, route and duration of drug administration, clinical indication, etc. For example, all participants of the ten studies had taken different antiresorptive drugs: bisphosphonates and denosumab in four studies (Hasegawa 2019 [38], Ottesen 2022 [29], Poxleitner 2020 [30], and Ristow 2021 [31]), denosumab in one study (Hasegawa 2021 [39]), and bisphosphonates in the other ten studies. There was not enough confidence that the participants were representative of the targeted population. 

#### 4.2.3. Reliability of Included Primary Studies

There had been no sample size calculation applied in all of the fifteen included primary studies, which caused imprecise evidence due to the small sample sizes.

#### 4.2.4. Limitations of This Evidence Mapping Review

The PRISMA 2020 statement [18] and the Cochrane Handbook for Systematic Reviews of Interventions [19] have been followed in this review in order to minimize the potential bias in the review process. The inclusion of nonrandomized studies in this review might have degraded the quality of evidence, since the risk of bias in NRSs are usually more serious than that in RCTs in most instances. Furthermore, there is not an analysis that is more appropriate enough for rare events in both groups [56], which has indicates the slight unlikelihood of the syntheses where the numbers of events in the two groups are both zero. There is still likely a potential for publication bias, but we were unable to detect it in this review because of the insufficient number of studies included.

### 4.3. Implications for Future Research

Prospective controlled clinical studies with good quality and large sample sizes will lead to better evidence. Double-blind RCTs are thought to be a best study design type; the blinding of participants, personnel and outcome assessment should be taken into consideration. Large enough sample sizes are necessary for further studies in order to be representative of the targeted population, which calls for sample size calculation in protocols. In nonrandomized studies, an appropriate analysis such as regression and inverse probability weighting with all confounding factors included should be undertaken to reduce confounding bias.

## 5. Conclusions

Fifteen primary studies, with a total of 3303 participants, and five reviews were included in this evidence mapping review. There is limited evidence to show that a drug holiday is likely to be unnecessary and might be potentially harmful in dental practice. The efficacy of application of APC (PRF, PRGF and PRP) in tooth extraction is uncertain due to limited evidence. Furthermore, the efficacy of different surgical techniques and antibacterial prophylaxis in tooth extraction is also unclear. However, primary closure and antibacterial prophylaxis are still recommended despite limited evidence. All evidence has been graded as either of low or very low level, thus sufficient further high-quality RCTs are needed to answer this clinical question.

## Figures and Tables

**Table 1:** Antiresorptive drugs, potential to cause MRONJ.

Generic Name	Brand Name	Primary Indication	Common Dose	Route
Bisphosphonates, first-generation (non-nitrogen-containing)				
Clodronate	Bonefos	malignant tumors	300 mg per day	intravenous
	Clasteon/Clastoban/Ostac	malignant tumors	400 mg per day	oral
Etidronate	Didronel	Paget’s disease	300–750 mg per day	oral
Tiludronate	Skelid	Paget’s disease	400 mg per day	oral
Bisphosphonates, second-generation (nitrogen-containing, with an amino terminal group)				
Alendronate	Binosto/Fosavance	osteoporosis	70 mg per week	oral
	Fosamax	osteoporosis	10 mg per day	oral
Neridronate *	Nerixia	Investigational **	-	-
Pamidronate	Aredia/Pamidria/Pamidonat/Pamifos/Pamisol	malignant tumors	90 mg every 3 weeks	intravenous
Bisphosphonates, third-generation (nitrogen-containing, with a cyclic side-chain or a tertiary amino group)				
Ibandronate	Boniva	osteoporosis	2.5 mg per day	oral
	Bondenza/Bonviva/Boniva	osteoporosis	150 mg per month	oral
	Bondenza/Bonviva/Boniva	malignant tumors	3 mg every 3 months	intravenous
	Bondronat/Iasibon	malignant tumors	2–6 mg every 3 months	intravenous
	Bondronat/Iasibon	malignant tumors	50 mg per day	oral
Minodronate	Bonteo/Onobis/Recalbon	Investigational **	-	-
Risedronate	Actonel	osteoporosis	5 mg per day	oral
	Actonel/Atelvia	osteoporosis	35 mg per week	oral
Zoledronate	Aclasta/Reclast	osteoporosis	5 mg per year	intravenous
	Zomera/Zometa	malignant tumors	4 mg every 3 weeks	intravenous
Humanized monoclonal antibody				
Denosumab	Prolia	osteoporosis	60 mg every 6 months	subcutaneous
	Xgeva	bone metastases	120 mg every 4 months	subcutaneous

* There has been no evidence that neridronate could cause MRONJ from the few published studies so far; however, neridronate is kept in this table due to its similar chemical structure with alendronate and pamidronate. ** Both neridronate and minodronate are not approved but are investigational in Europe and the U.S.

**Table 2:** Non-antiresorptive drugs, potential to cause MRONJ.

Mechanism of Action	Generic Name	Brand Name
Tyrosine kinase inhibitors (TKI)	Axitinib	Inlyta
	Cabozantinib	Cabometyx/Cometriq
	Dasatinib	Sprycel
	Erlotinib	Tarceva
	Imatinib	Gleevec/Glivec
	Pazopanib	Votrient
	Regorafenib	Stivarga
	Sorafenib	Nexavar
	Sunitinib	Sutent
B-Raf inhibitors	Dabrafenib	Tafinlar
	Trametinib	Mekinist
Mammalian target of rapamycin (mTOR) inhibitors	Rapamycin/Sirolimus	Rapamune
	Temsirolimus	Torisel
	Everolimus	Afinitor/Certican/Votubia/Zortress
Vascular endothelial growth factor (VEGF) inhibitors	Aflibercept	Eylea/Zaltrap
	Bevacizumab	Abevmy/Alymsys/Avastin/Aybintio/Bambevi/Equidacent/ Mvasi/Onbevzi/Oyavas/Zirabev
Monoclonal antibodies used in immunotherapy	Ipilimumab	Yervoy
	Nivolumab	Opdivo
	Rituximab	Blitzima/Mabthera/Riabni/Ritemvia/Rituxan/Rixathon/ Riximyo/Ruxience/Truxima

**Table 3:** Characteristics of included reviews.

Study ID	Methods	Findings	
		Outcomes and Relative Effect	Certainty
Beth-Tasdogan 2022 [25]	Design: systematic review Registration: Cochrane protocol Included studies: 13 RCTs	Intervention: extraction with PRGF Control: extraction without PRGF Incidence proportion of MRONJ RR 0.08 (95% CI 0.00 to 1.51) from one RCT	Very low * Very low ^#^
		Intervention: sub-periosteal wound closure Control: epi-periosteal wound closure Incidence proportion of MRONJ RR 0.09 (95% CI 0.00 to 1.56) from one RCT	Very low * Very low ^#^
Cabras 2021 [16]	Design: systematic review Registration: PROSPERO Included studies: 17 primary studies	Intervention: antibacterial prophylaxis Control: no antibacterial prophylaxis Efficacy not reported	Not reported * Very low ^#^
Del Fabbro 2015 [26]	Design: systematic review Registration: not reported Included studies: 18 primary studies	Intervention: extraction with PRGF Control: extraction without PRGF Incidence proportion of MRONJ OR 0.08 (95% CI 0.00 to 1.47) from one RCT	Not reported * Very low ^#^
Ottesen 2020 [15]	Design: systematic review Registration: PROSPERO Included studies: 14 primary studies	Intervention: drug holiday Control: drug continuation Efficacy reported as uncertain	Not reported * Very low ^#^
Sacco 2021 [24]	Design: umbrella review Registration: INPLASY Included studies: 25 systematic reviews	All comparisons of interventions Efficacy not reported	Very low * Very low ^#^

* Certainty of evidence assessed with GRADE by included review authors; ^#^ Certainty of evidence assessed with GRADE by this evidence mapping review authors.

**Table 4:** Characteristics of included primary studies.

Study ID	Methods	Participants	Outcomes
Asaka 2017 [32]	Design: HCT Region: Japan Period: 2013 to 2015	102 patients (Male/Female = 9/93; none withdrawn), median age 69, range from 24 to 88 Systemic conditions: metabolic bone diseases (all 102) Drugs *: alendronate (53), etidronate (5), minodronate (12), risedronate (49)	Follow-up: 3 months Primary: MRONJ, delayed healing
Bodem 2015 [35]	Design: PCS Region: Germany Period: Not reported	61 patients (Male/Female = 19/42; none withdrawn), mean age 65.65, ranged from 34 to 87 Systemic conditions: malignant tumors (all 61) Drugs: ibandronate (17), pamidronate (6), zoledronate (38)	Follow-up: 3 months Primary: MRONJ
Hasegawa 2017 [37]	Design: multicenter RCS Region: Japan Period: 2008 to 2015	1175 patients (Male/Female = 161/1014; none withdrawn), mean age 70.7, ranged from 23 to 102 Systemic conditions *: malignant tumors, metabolic bone diseases Drugs *: alendronate (742), risedronate (334), minodronate (129), other bisphosphonates (10), unknown (11)	Follow-up: more than 2 months Primary: MRONJ
Hasegawa 2019 [38]	Design: multicenter RCS Region: Japan Period: 2008 to 2016	85 patients (Male/Female = 34/51; none withdrawn), mean age 64.5, ranged from 39 to 90 Systemic conditions: malignant tumors (all 85) Drugs *: zoledronate (52), alendronate (1), risedronate (1), denosumab (39)	Follow-up: more than 2 months Primary: MRONJ
Hasegawa 2021 [39]	Design: multicenter RCS Region: Japan Period: 2008 to 2019	72 patients (Male/Female = 31/41; none withdrawn), mean age 65.2, ranged from 41 to 85 Systemic conditions: malignant tumors (all 72) Drugs: denosumab (all 72)	Follow-up: more than 2 months Primary: MRONJ
Kang 2020 [40]	Design: RCS Region: Korea Period: 2008 to 2017	465 patients (Male/Female = 45/420; none withdrawn), mean age 68.8 Systemic conditions: malignant tumors (6), metabolic bone diseases (458), unknown (1) Drugs *: alendronate (439), ibandronate (56)	Follow-up: more than 2 months Primary: MRONJ
Mauceri 2020 [33]	Design: HCT Region: Italy Period: 2015 to 2016	20 patients (prospective; none withdrawn) and 905 patients (retrospective from literature), age not reported Systemic conditions in prospective: malignant tumors (6), metabolic bone diseases (14) Drugs in prospective: alendronate (6), clodronate (4), ibandronate (2), risedronate (2), zoledronate (6)	Follow-up: 24 months in prospective Primary: MRONJ, delayed healing
Montefusco 2008 [48]	Design: multicenter RCS Region: Italy Period: till 2006	24 patients Systemic conditions: malignant tumors (all 24) Drugs *: pamidronate, zoledronate	Follow-up: more than 2 months Primary: MRONJ
Mozzati 2012 [27]	Design: RCT Region: Italy Period: 2005–2009	176 patients (Male/Female = 75/101; none withdrawn), age ranged from 44 to 83 Systemic conditions: malignant tumors (all 176) Drugs: zoledronate (all 176)	Follow-up: 24 to 60 months Primary: MRONJ, delayed healing
Mozzati 2013 [28]	Design: RCT Region: Italy Period: 2005–2011	700 patients (Male/Female = 23/677; none withdrawn), age ranged from 52 to 79 Systemic conditions: metabolic bone diseases (all 700) Drugs: alendronate (all 700)	Follow-up: 12 to 72 months Primary: MRONJ, delayed healing
Ottesen 2022 [29]	Design: Single-blind RCT Region: Denmark Period: 2018–2019	23 patients (Male/Female = 11/12; three withdrawn but evaluated in ITT analysis), age ranged from 56 to 78 Systemic conditions: malignant tumors (all 23) Drugs: bisphosphonates (pamidronate or zoledronate) (10), denosumab (13)	Follow-up: 6 months Primary: MRONJ, mortality Secondary: complications, QoL
Poxleitner 2020 [30]	Design: RCT Region: Germany Period: 2017–2019	77 patients (Male/Female = 1/76; none withdrawn), median age 78, ranged from 44 to 88 Systemic conditions: metabolic bone diseases (all 77) Drugs: alendronate (28), ibandronate (9), pamidronate (1), risedronate (8), zoledronate (7), denosumab (24)	Follow-up: 3 months Primary: MRONJ Secondary: complications
Ristow 2021 [31]	Design: Double-blind RCT Region: Germany Period: 2016–2018	160 patients (Male/Female = 43/117; 28 withdrawn but evaluated in ITT analysis), mean age 68.1 Systemic conditions: malignant tumors (87), metabolic bone diseases (73) Drugs *: bisphosphonates (130), denosumab (46)	Follow-up: 6 months Primary: MRONJ, mortality
Sanchis 2014 [36]	Design: PCS Region: Spain Period: 2009–2011	36 patients (Male/Female = 16/20; two withdrawn and not evaluated), mean age 63.81 Systemic conditions: malignant tumors (33), Crohn’s disease (1) Drugs: zoledronate (all 36)	Follow-up: 4 months Primary: MRONJ
Scoletta 2013 [34]	Design: HCT Region: Italy Period: 2010–2011	127 patients (Male/Female = 38/89; none withdrawn), mean age 65.31 Systemic conditions: malignant tumors (117), metabolic bone diseases (10) Drugs *: ibandronate (5), pamidronate (11), zoledronate (116)	Follow-up: 4 to 12 months Primary: MRONJ

* There was some overlapping for systemic conditions or drugs. Abbreviations: for study design, RCT = randomized controlled trial, HCT = historical controlled trial, RCS = retrospective cohort study, PCS = prospective cohort study.

**Table 5:** Comparison of different surgical techniques.

Study ID	Incidence of MRONJ among Different Surgical Techniques				
(Study Design)	A	B	C	D	E
Hasegawa 2017 [37] (RCS, 3-armed)	0/105 extractions	18/1470 extractions	23/855 extractions		
Hasegawa 2019 [38] (RCS, 3-armed)	0/2 extractions	22/85 extractions	17/57 extractions		
Hasegawa 2021 [39] (RCS, 3-armed)	1/5 patients (2/15 extractions)	12/40 patients (20/71 extractions)	12/27 patients (17/50 extractions)		
Mozzati 2013 [28] (RCT, 2-armed)	0/334 patients (0/620 extractions)	0/366 patients (0/860 extractions)			
Ristow 2021 [31] (RCT, 2-armed)	5/82 patients			11/78 patients	
Scoletta 2013 [34] (HCT, 2-armed)		1/63 patients			5/64 patients

Abbreviations: for study design, RCS = retrospective cohort study, RCT = randomized controlled trial, HCT = historical controlled trial; for surgical techniques, A = primary healing (with mucoperiosteal flap), B = secondary healing with wound closure (without flap), C = secondary healing with wound open (without suture), D = primary healing with mucosal flap, E = secondary healing with wound closure with mucoperiosteal flap.

**Table 6:** Standard antibiotic schedules reported in included primary studies.

Study ID		Antibiotic	Dose and Frequency	Route	Duration of Antibiotic Schedule
Asaka 2017 [32]	Preferred	Amoxicillin	250 mg q 8 h	Not reported	7 days, starting from the morning of the surgery
	Alternative	Clindamycin	150 mg q 6 h	Not reported	
Bodem 2015 [35]	Preferred	Ampicillin/sulbactam	1.5 g tid	Intravenous	≥6 days, starting at least 24 h before the surgery, and continuing 5 days after the surgery
	Alternative	Clindamycin	600 mg tid	Intravenous	
Montefusco 2008 [48]	Preferred	Amoxicillin/clavulanate	1 g bid	Oral	4 days, starting from 1 day before the surgery, and continuing 3 days after the surgery
	Alternative	Levofloxacin	500 mg qd	Oral	
Mozzati 2012 [27]	Preferred	Amoxicillin/clavulanate potassium	1 tablet (1 g) q 8 h	Oral	6 days, starting from the evening before the surgery
	Alternative	Erythromycin	1 tablet (600 mg) q 8 h	Oral	
Mozzati 2013 [28]	Preferred	Amoxicillin/clavulanatic acid	1 tablet q 12 h	Oral	6 days, starting from the evening before the surgery
	Alternative	Erythromycin	1 tablet q 8 h	Oral	
Ottesen 2022 [29]	Preferred	Amoxicillin/clavulanatic acid	1000/250 mg for the first time before the surgery; 500/125 mg tid after the surgery	Oral	10 days, starting from 1 h before the surgery
	Alternative	Erythromycin	600 mg for the first time before the surgery; 300 mg tid after the surgery	Oral	
Poxleitner 2020 [30]	Preferred	Penicillin	10,000,000 IU qd	Intravenous	2 days, starting from 1 day before the surgery, and continuing 1 day after the surgery
	Alternative	Clindamycin	600 mg tid	Intravenous	
Ristow 2021 [31]	Preferred	Sultamicillin	375 mg (frequency not reported)	Oral	≥7 days, starting on the week before the surgery, and continuing 1 week after surgery
	Alternative	Clindamycin	600 mg (frequency not reported)	Oral	
Sanchis 2014 [36]	Preferred	Amoxicillin/clavulanatic acid	875/125 mg mg q 8 h	Not reported	17 days, starting from 2 days before the surgery, and continuing 15 days after surgery
	Alternative	Clindamycin	300 mg q 8 h	Not reported	
Scoletta 2013 [34]	Preferred	Amoxicillin/clavulanate potassium	1 tablet (1 g) q 8 h	Oral	6 days, starting from the evening before the surgery
	Alternative	Erythromycin	1 tablet (600 mg) q 8 h	Oral	

Alternative antibiotics used in case of potential allergy to the preferred antibiotics.

## Data Availability

All data generated or analyzed during this study are included in this published article and its Supplementary Materials.

## Supplementary Materials

The following supporting information can be downloaded at: https://www.mdpi.com/article/10.3390/jcm12010239/s1, Document: Supplementary Material (71 pages in total). Contents as follows: 1. Summary of Searches (p. 4). 2. PRISMA 2020 flow diagram (p. 5). 3. Search Strategies (pp. 6–12). 4. Characteristics and Risk-of-Bias Assessment of Included Reviews (5 studies with 6 reports) (pp. 13–22). 5. Characteristics and Risk-of-Bias Assessment of Included Primary Studies (15 studies with 21 reports) (pp. 23–52). 6. Characteristics of Excluded Studies (46 studies with 57 reports) (pp. 53–58). 7. Analysis (Forest plots) (pp. 59–66). 8. Summary of Findings Tables (SoF tables) (pp. 67–68). 9. Appendix (Risk of bias assessment tools) (pp. 69–71).
