# Supplementary material for: Management of Tooth Extraction in Patients Taking Antiresorptive Drugs: An Evidence Mapping Review and Meta-Analysis
Source: J Clin Med. 2022 Dec 28;12(1):239. doi: 10.3390/jcm12010239 (PMC9821631; doi:10.3390/jcm12010239)
Supplement: Supplementary file 1 [file jcm-12-00239-s001.zip › jcm-2109397-supplementary.pdf]

Supplementary Material

This document is a supplementary material for a manuscript entitled *Management of tooth extraction in patients taking antiresorptive drugs: an evidence mapping review and meta-analysis*.

Contents

Summary of Searches 4
2022-05-25 (first search) 4
2022-11-30 (last search) 4
PRISMA 2020 flow diagram 5
Flow diagram (first search on 5 May 2022) 5
Flow diagram (last search on 30 November 2022) 5
Search Strategies 6
Ovid Medline and Epub Ahead of Print, In-Process, In-Data-Review & Other Non-Indexed Citations, Daily and Versions: 1946 to November 29, 2022 6
Ovid EMBase: 1974 to 2022 November 29 7
The Cochrane Library: 30 November 2022 8
Scopus via Elsevier: 2022-11-30 9
Web of Science Core Collection (WOSCC) via Clarivate: 1900 to 2022-11-27 10
Inspec via Clarivate: 1969 to 2022-11-27 10
Korean Science Citation Index-Korean Journal Database (KCI-KJD) via Clarivate: 1980 to 2022-11-18 10
Science Electronic Library Online Citation Index (SciELO) via Clarivate: 2002 to 2022-11-28 11
WHO Global Index Medicus (GIM): 2022-11-30 11
WHO International Clinical Trials Registry Platform (ICTRP): Version 3.6 12
ClinicalTrials.gov via United States (U.S.) National Library of Medicine (NIH): 2022-11-30 12
Characteristics and Risk-of-Bias Assessment of Included Reviews (5 studies with 6 reports) 13
Beth-Tasdogan 2022 13
Risk of Bias (ROBIS) 14
References 14
Cabras 2021 15
Risk of Bias (ROBIS) 16
References 16
Del Fabbro 2015 17
Risk of Bias (ROBIS) 18
References 18
Ottesen 2020 19
Risk of Bias (ROBIS) 20
References 20
Sacco 2021 21
Risk of Bias (ROBIS) 22
References 22
Characteristics and Risk-of-Bias Assessment of Included Primary Studies (15 studies with 21 reports) 23
Asaka 2017 23
Risk of Bias (ROBINS-I) 24
References 24
Bodem 2015 25

Risk of Bias (ROBINS-I) ... 26
References ... 26
Hasegawa 2017 ... 27
Risk of Bias (ROBINS-I) ... 27
References ... 28
Hasegawa 2019 ... 29
Risk of Bias (ROBINS-I) ... 30
References ... 30
Hasegawa 2021 ... 31
Risk of Bias (ROBINS-I) ... 32
References ... 32
Kang 2020 ... 33
Risk of Bias (ROBINS-I) ... 34
References ... 34
Mauceri 2020 ... 35
Risk of Bias (ROBINS-I) ... 36
References ... 36
Montefusco 2008 ... 37
Risk of Bias (ROBINS-I) ... 38
References ... 38
Mozzati 2012 ... 39
Risk of Bias (RoB 2) ... 40
References ... 40
Mozzati 2013 ... 41
Risk of Bias (RoB 2) ... 42
References ... 42
Ottesen 2022 ... 43
Risk of Bias (RoB 2) ... 44
References ... 44
Poxleitner 2020 ... 45
Risk of Bias (RoB 2) ... 46
References ... 46
Ristow 2021 ... 47
Risk of Bias (RoB 2) ... 48
References ... 48
Sanchis 2014 ... 49
Risk of Bias (ROBINS-I) ... 50
References ... 50
Scoletta 2013 ... 51
Risk of Bias (ROBINS-I) ... 52
References ... 52
Characteristics of Excluded Studies (46 studies with 57 reports) ... 53
Primary studies excluded due to focused clinical questions (10 studies with 12 reports) ... 53
Primary studies excluded due to study design (30 studies with 39 reports) ... 55
Primary studies ongoing or awaiting classification (4 studies with 4 reports) ... 58
Secondary studies excluded due to focused clinical questions (2 studies with 2 reports) ... 58
Analysis (Forest plots) ... 59
Comparison 1: Drug holiday versus drug continuation ... 59

1.1 Prevalence of delayed healing ... 59
1.2 Incidence proportion of MRONJ ... 59
1.3 Mortality (MRONJ-related) ... 60
1.4 Mortality (all-cause) ... 61
Comparison 2: APC versus control ... 62
2.1 PRF versus control ... 62
2.2 PRGF versus control ... 63
2.3 PRP versus control ... 63
Comparison 3: Different surgical techniques ... 64
3.1 Secondary healing versus primary healing ... 64
3.2 Primary healing with mucoperiosteal flap versus mucosal flap ... 65
3.3 Secondary healing with mucoperiosteal flap versus no flap ... 66
Comparison 4: Antibacterial prophylaxis versus control ... 66
4.1 Incidence proportion of MRONJ ... 66
Summary of findings tables (SoF tables) ... 67
Appendix (Risk of bias assessment tools) ... 69
Appendix 1: ROBIS tool (Risk of Bias in Systematic Reviews) ... 69
Signaling questions ... 69
References ... 69
Appendix 2: RoB 2 tool (Revised Cochrane Risk-of-Bias tool for randomized trials) ... 69
Signaling questions ... 69
References ... 70
Appendix 3: ROBINS-I tool (Risk of Bias in Nonrandomized Studies of Interventions) ... 70
Signaling questions ... 70
References ... 71

Summary of Searches

2022-05-25 (first search)

Databases/Registers	Version/Issue	Date of search	Records retrieved	Methods for screening
Medline via Ovid	1946 to May 24, 2022	2022-05-25	1156	EndNote Desktop
EMBase via Ovid	1974 to 2022 May 24	2022-05-25	1880	EndNote Desktop
Cochrane Library	-	2022-05-25	81	Online
Scopus	-	2022-05-25	1560	EndNote Desktop
WOSCC via Clarivate	Update to 2022-05-23	2022-05-25	977	EndNote Desktop
Inspec via Clarivate	Update to 2022-05-22	2022-05-25	12	EndNote Desktop
KCI-KJD via Clarivate	Update to 2022-05-18	2022-05-25	42	EndNote Desktop
SciELO via Clarivate	Update to 2022-05-21	2022-05-25	25	EndNote Desktop
GIM	-	2022-05-25	100	EndNote Desktop
ICTRP	Version 3.6	2022-05-25	71	EndNote Desktop
ClinicalTrials.gov	-	2022-05-25	32	EndNote Desktop

Total 5904 records identified from nine databases and two registers:

81 records identified and screened online: 81 records from the Cochrane Library;

5855 records identified and imported into EndNote Desktop: 5752 records from the other eight databases, and 103 records from two registers (ICTRP and ClinicalTrials.gov); **3095 records screened on EndNote Desktop** after **2760 duplicate records removed on EndNote Desktop**.

2022-11-30 (last search)

Databases/Registers	Version/Issue	Date of search	Records retrieved	Methods for screening
Medline via Ovid	1946 to November 29, 2022	2022-11-30	1192	EndNote Desktop
EMBase via Ovid	1974 to 2022 November 29	2022-11-30	1946	EndNote Desktop
Cochrane Library	-	2022-11-30	83	Online
Scopus	-	2022-11-30	1625	EndNote Desktop
WOSCC via Clarivate	Update to 2022-11-27	2022-11-30	1024	EndNote Desktop
Inspec via Clarivate	Update to 2022-11-27	2022-11-30	13	EndNote Desktop
KCI-KJD via Clarivate	Update to 2022-11-18	2022-11-30	44	EndNote Desktop
SciELO via Clarivate	Update to 2022-11-28	2022-11-30	30	EndNote Desktop
GIM	-	2022-11-30	109	EndNote Desktop
ICTRP	Version 3.6	2022-11-30	76	EndNote Desktop
ClinicalTrials.gov	-	2022-11-30	32	EndNote Desktop

Total 6174 records identified from nine databases and two registers:

83 records identified and screened online: 83 records **(2 new records)** from the Cochrane Library;

6091 records identified and imported into EndNote Desktop: 5983 records from the other eight databases, and 108 records from two registers (ICTRP and ClinicalTrials.gov); **3221 records (126 new records) screened on EndNote Desktop** after **2870 duplicate records removed on EndNote Desktop**.

PRISMA 2020 flow diagram

Flow diagram (first search on 5 May 2022)

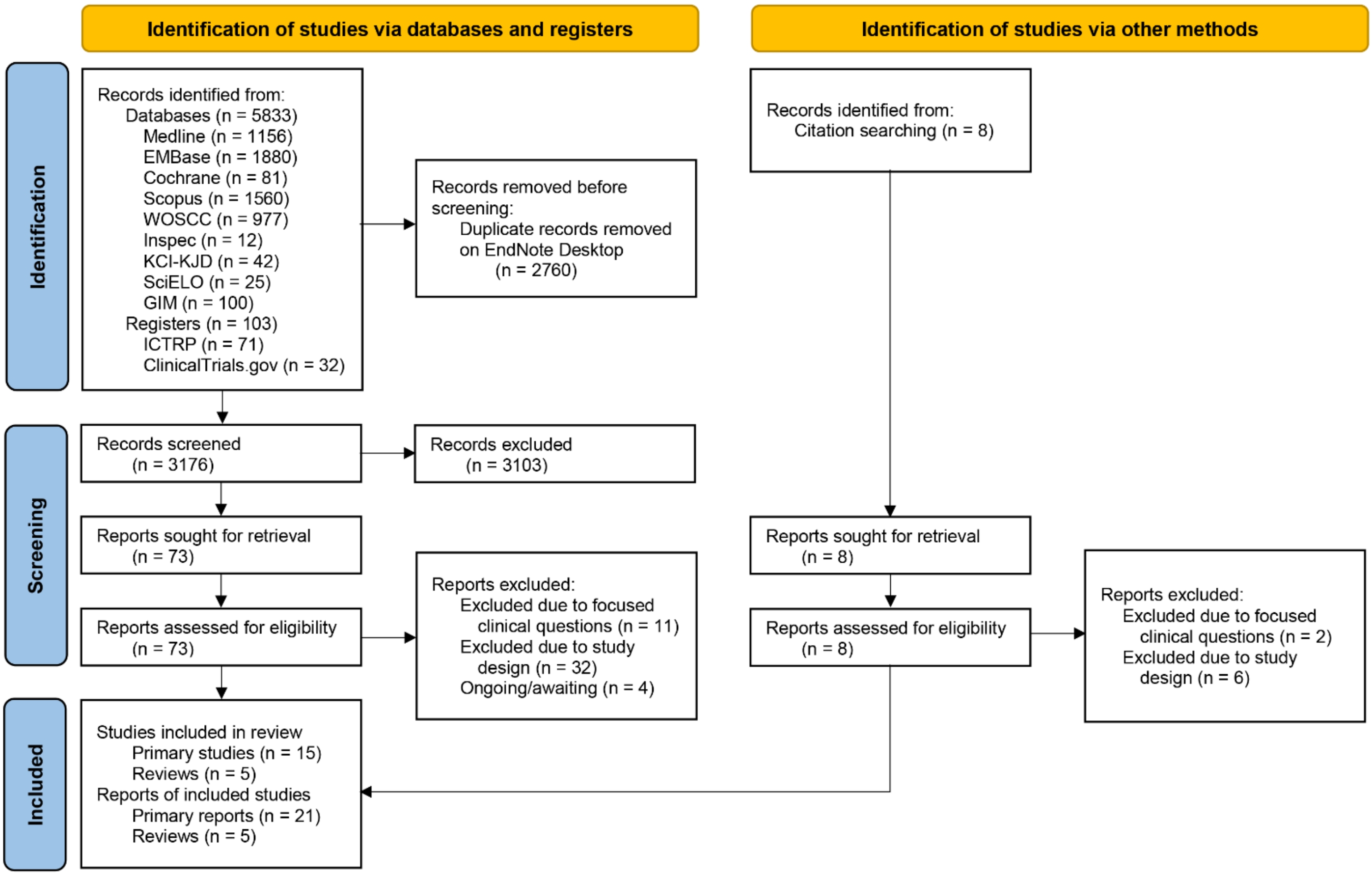

Flow diagram (last search on 30 November 2022)

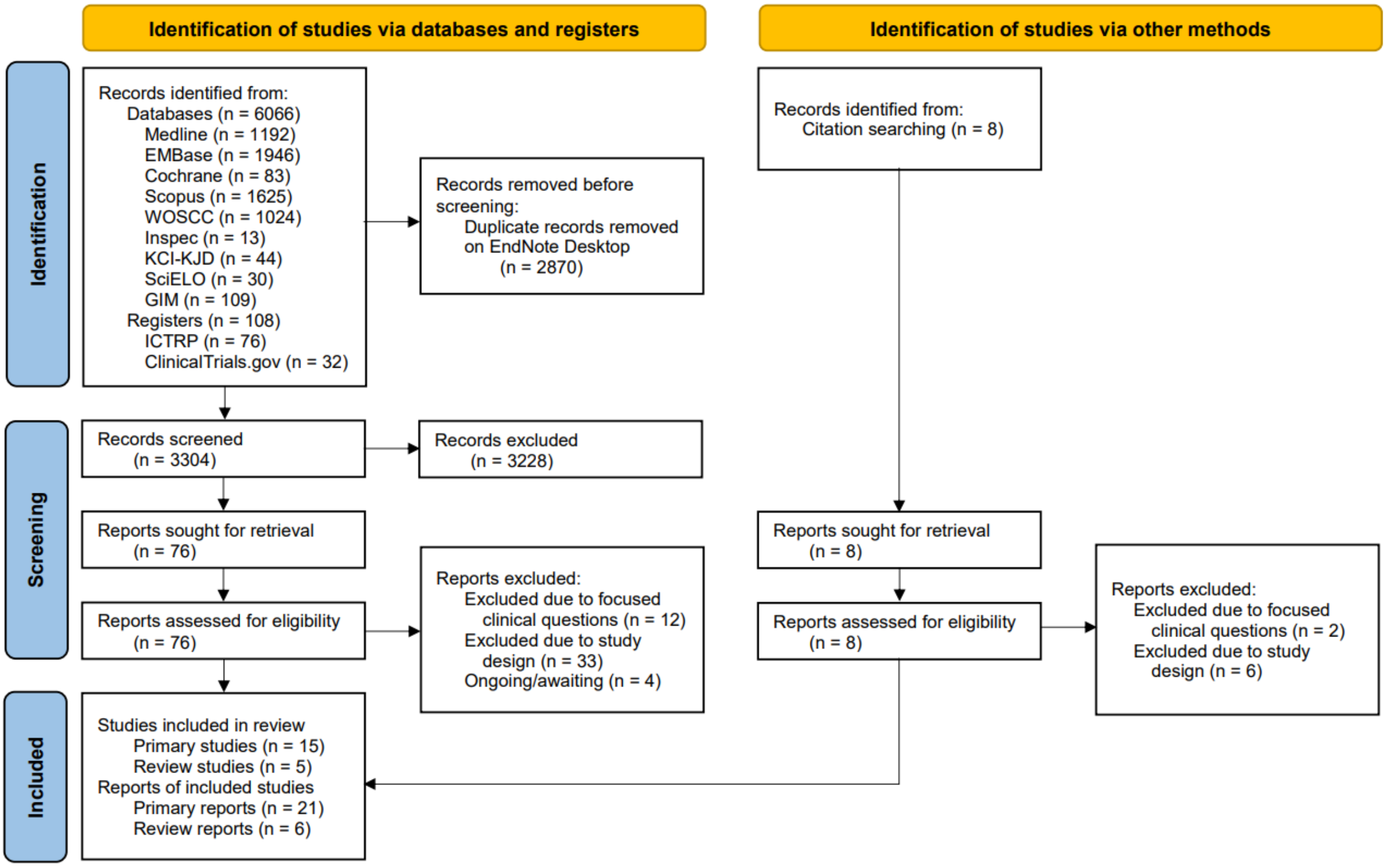

Search Strategies

Ovid Medline and Epub Ahead of Print, In-Process, In-Data-Review & Other Non-Indexed Citations, Daily and Versions: 1946 to November 29, 2022

Website: https://ovidsp.ovid.com/

# ▲	Searches	Results
1	exp Jaw/	113573
2	exp Jaw Diseases/	104628
3	(jaw* or mandib* or maxill* or alveolar or dentoalveolar).mp.	364468
4	or/1-3	414960
5	exp Osteonecrosis/	17298
6	exp Bone Diseases, Infectious/	40622
7	(osteonecro* or "bone necrosis" or osteochemonecro*).mp.	17019
8	or/5-7	62713
9	exp Bone Density Conservation Agents/	143451
10	exp Diphosphonates/	27923
11	exp Angiogenesis Inhibitors/	65791
12	exp Antineoplastic Agents/	1223307
13	(agent* or anti-resorptive or antiresorptive or drug* or medication*).mp.	7649549
14	(bisphosphon* or aminobisphosphon* or diphosphon*).mp.	30670
15	(alendron* or Binosto or Fosamax or Fosavance).mp.	6100
16	(clodron* or Bonefos or Clasteon or Clastoban or Ostac).mp.	2960
17	(etidron* or Didronel).mp.	3327
18	(ibandron* or Boniva or Bondenza or Bonviva or Bondronat or Iasibon).mp.	1266
19	(minodron* or Bonteo or Onobis or Recalbon or "ONO 5920" or ONO5920 or "YH 529" or YH529 or "YM 529" or YM529).mp.	191
20	(neridron* or Nerixia).mp.	143
21	(pamidron* or Aredia or Pamidria or Pamidonat or Pamifos or Pamisol).mp.	3286
22	(risedron* or Actonel or Atelvia).mp.	2138
23	(tiludron* or Skelid).mp.	174
24	(zoledron* or Aclasta or Reclast or Zomera or Zometa).mp.	6210
25	(denosumab or Xgeva or Prolia).mp.	4146
26	(aflibercept or Eylea or Zaltrap).mp.	3153
27	(axitinib or Inlyta).mp.	1387
28	(bevacizumab or Abevmy or Alymsys or Avastin or Aybintio or Bambevi or Equidacent or Mvasi or Onbevzi or Oyavas or Zirabev).mp.	21980
29	(cabozantinib or Cabometyx or Cometriq or bms907351 or "bms 907351" or xl184 or "xl 184").mp.	1457
30	(dabrafenib or Tafinlar).mp.	1623
31	(dasatinib or Sprycel).mp.	4371
32	(erlotinib or Tarceva).mp.	7808
33	(everolimus or Afinitor or Certican or Votubia or Zortress or rad001 or "rad 001" or "sdzrad" or "sdz rad").mp.	8771
34	(imatinib or Gleevec or Glivec).mp.	17382
35	(ipilimumab or Yervoy).mp.	5148
36	(nivolumab or Opdivo).mp.	8797
37	(pazopanib or Votrient).mp.	2214

38	(rapamycin or sirolimus or Rapamune).mp.	49812
39	(regorafenib or Stivarga).mp.	1745
40	(rituximab or Blitzima or Mabthera or Riabni or Ritemvia or Rituxan or Rixathon or Riximyo or Ruxience or Truxima).mp.	29490
41	(sorafenib or Nexavar).mp.	11185
42	(sunitinib or Sutent or su11248 or "su 11248").mp.	7229
43	(temsirolimus or Torisel).mp.	1648
44	(trametinib or Mekinist).mp.	1935
45	or/9-44	7944586
46	4 and 8 and 45	4899
47	(phossy jaw* or bisphossy jaw*).mp.	24
48	(ARONJ or BRONJ or BONJ or MRONJ).mp.	1555
49	exp "Bisphosphonate-Associated Osteonecrosis of the Jaw"/	1633
50	or/46-49	4926
51	(extract* or remov* or exodont*).mp.	1759239
52	exp Tooth Extraction/	21182
53	or/51-52	1759239
**54**	**50 and 53**	1192

Ovid EMBase: 1974 to 2022 November 29

Website: https://ovidsp.ovid.com/

# ▲	Searches	Results
1	exp Jaw/	21597
2	exp Jaw Diseases/	116695
3	(jaw* or mandib* or maxill* or alveolar or dentoalveolar).mp.	395870
4	or/1-3	430520
5	exp Osteonecrosis/	43332
6	exp Bone Diseases, Infectious/	11526
7	(osteonecro* or "bone necrosis" or osteochemonecro*).mp.	27969
8	or/5-7	57166
9	exp Bone Density Conservation Agents/	4035
10	exp Diphosphonates/	74945
11	exp Angiogenesis Inhibitors/	351910
12	exp Antineoplastic Agents/	2629705
13	(agent* or anti-resorptive or antiresorptive or drug* or medication*).mp.	13708213
14	(bisphosphon* or aminobisphosphon* or diphosphon*).mp.	53341
15	(alendron* or Binosto or Fosamax or Fosavance).mp.	18728
16	(clodron* or Bonefos or Clasteon or Clastoban or Ostac).mp.	7718
17	(etidron* or Didronel).mp.	8935
18	(ibandron* or Boniva or Bondenza or Bonviva or Bondronat or Iasibon).mp.	5958
19	(minodron* or Bonteo or Onobis or Recalbon or "ONO 5920" or ONO5920 or "YH 529" or YH529 or "YM 529" or YM529).mp.	505
20	(neridron* or Nerixia).mp.	535
21	(pamidron* or Aredia or Pamidria or Pamidonat or Pamifos or Pamisol).mp.	11520
22	(risedron* or Actonel or Atelvia).mp.	8757
23	(tiludron* or Skelid).mp.	925

24	(zoledron* or Aclasta or Reclast or Zomera or Zometa).mp.	19777
25	(denosumab or Xgeva or Prolia).mp.	12455
26	(aflibercept or Eylea or Zaltrap).mp.	8567
27	(axitinib or Inlyta).mp.	6758
28	(bevacizumab or Abevmy or Alymsys or Avastin or Aybintio or Bambevi or Equidacent or Mvasi or Onbevzi or Oyavas or Zirabev).mp.	71940
29	(cabozantinib or Cabometyx or Cometriq or bms907351 or "bms 907351" or xl184 or "xl 184").mp.	6430
30	(dabrafenib or Tafinlar).mp.	6617
31	(dasatinib or Sprycel).mp.	17175
32	(erlotinib or Tarceva).mp.	31814
33	(everolimus or Afinitor or Certican or Votubia or Zortress or rad001 or "rad 001" or "sdzrad" or "sdz rad").mp.	35612
34	(imatinib or Gleevec or Glivec).mp.	49177
35	(ipilimumab or Yervoy).mp.	22572
36	(nivolumab or Opdivo).mp.	33688
37	(pazopanib or Votrient).mp.	10242
38	(rapamycin or sirolimus or Rapamune).mp.	128538
39	(regorafenib or Stivarga).mp.	6271
40	(rituximab or Blitzima or Mabthera or Riabni or Ritemvia or Rituxan or Rixathon or Riximyo or Ruxience or Truxima).mp.	107278
41	(sorafenib or Nexavar).mp.	36838
42	(sunitinib or Sutent or su11248 or "su 11248").mp.	27153
43	(temsirolimus or Torisel).mp.	9102
44	(trametinib or Mekinist).mp.	8201
45	or/9-44	14064764
46	4 and 8 and 45	8479
47	(phossy jaw* or bisphossy jaw*).mp.	27
48	(ARONJ or BRONJ or BONJ or MRONJ).mp.	1865
49	exp "Bisphosphonate-Associated Osteonecrosis of the Jaw"/	170
50	or/46-49	8532
51	(extract* or remov* or exodont*).mp.	2389680
52	exp Tooth Extraction/	25711
53	or/51-52	2389680
**54**	**50 and 53**	1946

The Cochrane Library: 30 November 2022

Website: https://www.cochranelibrary.com/

ID	Searches	Hits
#1	MeSH descriptor: [Jaw] explode all trees	3668
#2	MeSH descriptor: [Jaw Diseases] explode all trees	2593
#3	jaw* or mandib* or maxill* or alveolar or dentoalveolar	27930
#4	#1 or #2 or #3	29096
#5	MeSH descriptor: [Osteonecrosis] explode all trees	297
#6	MeSH descriptor: [Bone Diseases, Infectious] explode all trees	426
#7	osteonecro* or "bone necrosis" or osteochemonecro*	1132
#8	#5 or #6 or #7	1634
#9	MeSH descriptor: [Bone Density Conservation Agents] explode all trees	1699

#10	MeSH descriptor: [Diphosphonates] explode all trees	2698
#11	MeSH descriptor: [Angiogenesis Inhibitors] explode all trees	1389
#12	MeSH descriptor: [Antineoplastic Agents] explode all trees	13345
#13	(agent* or anti-resorptive or antiresorptive or drug* or medication*) or (bisphosphon* or aminobisphosphon* or diphosphon*) or (alendron* or Binosto or Fosamax or Fosavance) or (clodron* or Bonefos or Clasteon or Clastoban or Ostac) or (etidron* or Didronel) or (ibandron* or Boniva or Bondenza or Bonviva or Bondronat or Iasibon) or (minodron* or Bonteo or Onobis or Recalbon or "ONO 5920" or ONO5920 or "YH 529" or YH529 or "YM 529" or YM529) or (neridron* or Nerixia) or (pamidron* or Aredia or Pamidria or Pamidonat or Pamifos or Pamisol) or (risedron* or Actonel or Atelvia) or (tiludron* or Skelid) or (zoledron* or Aclasta or Reclast or Zomera or Zometa) or (denosumab or Xgeva or Prolia) or (aflibercept or Eylea or Zaltrap) or (axitinib or Inlyta) or (bevacizumab or Abevmy or Alymsys or Avastin or Aybintio or Bambevi or Equidacent or Mvasi or Onbevzi or Oyavas or Zirabev) or (cabozantinib or Cabometyx or Cometriq or bms907351 or "bms 907351" or xl184 or "xl 184") or (dabrafenib or Tafinlar) or (dasatinib or Sprycel) or (erlotinib or Tarceva) or (everolimus or Afinitor or Certican or Votubia or Zortress or rad001 or "rad 001" or "sdzrad" or "sdz rad") or (imatinib or Gleevec or Glivec) or (ipilimumab or Yervoy) or (nivolumab or Opdivo) or (pazopanib or Votrient) or (rapamycin or sirolimus or Rapamune) or (regorafenib or Stivarga) or (rituximab or Blitzima or Mabthera or Riabni or Ritemvia or Rituxan or Rixathon or Riximyo or Ruxience or Truxima) or (sorafenib or Nexavar) or (sunitinib or Sutent or su11248 or "su 11248") or (temsirolimus or Torisel) or (trametinib or Mekinist)	801427
#14	#9 or #10 or #11 or #12 or #13	801618
#15	#4 and #8 and #14	402
#16	(phossy jaw*) or (bisphossy jaw*) or ARONJ or BRONJ or BONJ or MRONJ	59
#17	MeSH descriptor: [Bisphosphonate-Associated Osteonecrosis of the Jaw] explode all trees	33
#18	#15 or #16 or #17	403
#19	MeSH descriptor: [Tooth Extraction] explode all trees	1963
#20	extract* or remov* or exodont*	89699
#21	#19 or #20	89699
**#22**	**#18 and #21**	**83**

Scopus via Elsevier: 2022-11-30
Website: https://www.scopus.com/
Search:
TITLE-ABS-KEY(((jaw* or mandib* or maxill* or alveolar or dentoalveolar) and (osteonecro* or "bone necrosis" or osteochemonecro*) and ((agent* or anti-resorptive or antiresorptive or drug* or medication*) or (bisphosphon* or aminobisphosphon* or diphosphon*) or (alendron* or Binosto or Fosamax or Fosavance) or (clodron* or Bonefos or Clasteon or Clastoban or Ostac) or (etidron* or Didronel) or (ibandron* or Boniva or Bondenza or Bonviva or Bondronat or Iasibon) or (minodron* or Bonteo or Onobis or Recalbon or "ONO 5920" or ONO5920 or "YH 529" or YH529 or "YM 529" or YM529) or (neridron* or Nerixia) or (pamidron* or Aredia or Pamidria or Pamidonat or Pamifos or Pamisol) or (risedron* or Actonel or Atelvia) or (tiludron* or Skelid) or (zoledron* or Aclasta or Reclast or Zomera or Zometa) or (denosumab or Xgeva or Prolia) or (aflibercept or Eylea or Zaltrap) or (axitinib or Inlyta) or (bevacizumab or Abevmy or Alymsys or Avastin or Aybintio or Bambevi or Equidacent or Mvasi or Onbevzi or Oyavas or Zirabev) or (cabozantinib or Cabometyx or Cometriq or bms907351 or "bms 907351" or xl184 or "xl 184") or (dabrafenib or Tafinlar) or (dasatinib or Sprycel) or (erlotinib or Tarceva) or (everolimus or Afinitor or Certican or Votubia or Zortress or rad001 or "rad 001" or "sdzrad" or "sdz rad") or (imatinib or Gleevec or Glivec) or (ipilimumab or Yervoy) or (nivolumab or Opdivo) or (pazopanib or Votrient) or (rapamycin or sirolimus or Rapamune) or (regorafenib or Stivarga) or (rituximab or Blitzima or Mabthera or Riabni or Ritemvia or Rituxan or Rixathon or Riximyo or Ruxience or Truxima) or (sorafenib or Nexavar) or (sunitinib or Sutent or su11248 or "su 11248") or (temsirolimus or Torisel) or (trametinib or Mekinist)) or ("phossy jaw*" or "bisphossy jaw*") or (ARONJ or BRONJ or

BONJ or MRONJ)) and (extract* or remov* or exodont*))
Results: 1625

Web of Science Core Collection (WOSCC) via Clarivate: 1900 to 2022-11-27
Website: https://www.webofscience.com/wos/woscc/
Search:
TS=(((jaw* or mandib* or maxill* or alveolar or dentoalveolar) and (osteonecro* or "bone necrosis" or osteochemonecro*) and ((agent* or anti-resorptive or antiresorptive or drug* or medication*) or (bisphosphon* or aminobisphosphon* or diphosphon*) or (alendron* or Binosto or Fosamax or Fosavance) or (clodron* or Bonefos or Clasteon or Clastoban or Ostac) or (etidron* or Didronel) or (ibandron* or Boniva or Bondenza or Bonviva or Bondronat or Iasibon) or (minodron* or Bonteo or Onobis or Recalbon or "ONO 5920" or ONO5920 or "YH 529" or YH529 or "YM 529" or YM529) or (neridron* or Nerixia) or (pamidron* or Aredia or Pamidria or Pamidonat or Pamifos or Pamisol) or (risedron* or Actonel or Atelvia) or (tiludron* or Skelid) or (zoledron* or Aclasta or Reclast or Zomera or Zometa) or (denosumab or Xgeva or Prolia) or (aflibercept or Eylea or Zaltrap) or (axitinib or Inlyta) or (bevacizumab or Abevmy or Alymsys or Avastin or Aybintio or Bambevi or Equidacent or Mvasi or Onbevzi or Oyavas or Zirabev) or (cabozantinib or Cabometyx or Cometriq or bms907351 or "bms 907351" or xl184 or "xl 184") or (dabrafenib or Tafinlar) or (dasatinib or Sprycel) or (erlotinib or Tarceva) or (everolimus or Afinitor or Certican or Votubia or Zortress or rad001 or "rad 001" or "sdzrad" or "sdz rad") or (imatinib or Gleevec or Glivec) or (ipilimumab or Yervoy) or (nivolumab or Opdivo) or (pazopanib or Votrient) or (rapamycin or sirolimus or Rapamune) or (regorafenib or Stivarga) or (rituximab or Blitzima or Mabthera or Riabni or Ritemvia or Rituxan or Rixathon or Riximyo or Ruxience or Truxima) or (sorafenib or Nexavar) or (sunitinib or Sutent or su11248 or "su 11248") or (temsirolimus or Torisel) or (trametinib or Mekinist)) or ("phossy jaw*" or "bisphossy jaw*") or (ARONJ or BRONJ or BONJ or MRONJ)) and (extract* or remov* or exodont*))
Results: 1024

Inspec via Clarivate: 1969 to 2022-11-27
Website: https://www.webofscience.com/wos/inspec/
Search:
TS=(((jaw* or mandib* or maxill* or alveolar or dentoalveolar) and (osteonecro* or "bone necrosis" or osteochemonecro*) and ((agent* or anti-resorptive or antiresorptive or drug* or medication*) or (bisphosphon* or aminobisphosphon* or diphosphon*) or (alendron* or Binosto or Fosamax or Fosavance) or (clodron* or Bonefos or Clasteon or Clastoban or Ostac) or (etidron* or Didronel) or (ibandron* or Boniva or Bondenza or Bonviva or Bondronat or Iasibon) or (minodron* or Bonteo or Onobis or Recalbon or "ONO 5920" or ONO5920 or "YH 529" or YH529 or "YM 529" or YM529) or (neridron* or Nerixia) or (pamidron* or Aredia or Pamidria or Pamidonat or Pamifos or Pamisol) or (risedron* or Actonel or Atelvia) or (tiludron* or Skelid) or (zoledron* or Aclasta or Reclast or Zomera or Zometa) or (denosumab or Xgeva or Prolia) or (aflibercept or Eylea or Zaltrap) or (axitinib or Inlyta) or (bevacizumab or Abevmy or Alymsys or Avastin or Aybintio or Bambevi or Equidacent or Mvasi or Onbevzi or Oyavas or Zirabev) or (cabozantinib or Cabometyx or Cometriq or bms907351 or "bms 907351" or xl184 or "xl 184") or (dabrafenib or Tafinlar) or (dasatinib or Sprycel) or (erlotinib or Tarceva) or (everolimus or Afinitor or Certican or Votubia or Zortress or rad001 or "rad 001" or "sdzrad" or "sdz rad") or (imatinib or Gleevec or Glivec) or (ipilimumab or Yervoy) or (nivolumab or Opdivo) or (pazopanib or Votrient) or (rapamycin or sirolimus or Rapamune) or (regorafenib or Stivarga) or (rituximab or Blitzima or Mabthera or Riabni or Ritemvia or Rituxan or Rixathon or Riximyo or Ruxience or Truxima) or (sorafenib or Nexavar) or (sunitinib or Sutent or su11248 or "su 11248") or (temsirolimus or Torisel) or (trametinib or Mekinist)) or ("phossy jaw*" or "bisphossy jaw*") or (ARONJ or BRONJ or BONJ or MRONJ)) and (extract* or remov* or exodont*))
Results: 13

Korean Science Citation Index-Korean Journal Database (KCI-KJD) via Clarivate: 1980 to 2022-11-18
Website: https://www.webofscience.com/wos/kjd/
Search:

TS=(((jaw* or mandib* or maxill* or alveolar or dentoalveolar) and (osteonecro* or "bone necrosis" or osteochemonecro*) and ((agent* or anti-resorptive or antiresorptive or drug* or medication*) or (bisphosphon* or aminobisphosphon* or diphosphon*) or (alendron* or Binosto or Fosamax or Fosavance) or (clodron* or Bonefos or Clasteon or Clastoban or Ostac) or (etidron* or Didronel) or (ibandron* or Boniva or Bondenza or Bonviva or Bondronat or Iasibon) or (minodron* or Bonteo or Onobis or Recalbon or "ONO 5920" or ONO5920 or "YH 529" or YH529 or "YM 529" or YM529) or (neridron* or Nerixia) or (pamidron* or Aredia or Pamidria or Pamidonat or Pamifos or Pamisol) or (risedron* or Actonel or Atelvia) or (tiludron* or Skelid) or (zoledron* or Aclasta or Reclast or Zomera or Zometa) or (denosumab or Xgeva or Prolia) or (aflibercept or Eylea or Zaltrap) or (axitinib or Inlyta) or (bevacizumab or Abevmy or Alymsys or Avastin or Aybintio or Bambevi or Equidacent or Mvasi or Onbevzi or Oyavas or Zirabev) or (cabozantinib or Cabometyx or Cometriq or bms907351 or "bms 907351" or xl184 or "xl 184") or (dabrafenib or Tafinlar) or (dasatinib or Sprycel) or (erlotinib or Tarceva) or (everolimus or Afinitor or Certican or Votubia or Zortress or rad001 or "rad 001" or "sdzrad" or "sdz rad") or (imatinib or Gleevec or Glivec) or (ipilimumab or Yervoy) or (nivolumab or Opdivo) or (pazopanib or Votrient) or (rapamycin or sirolimus or Rapamune) or (regorafenib or Stivarga) or (rituximab or Blitzima or Mabthera or Riabni or Ritemvia or Rituxan or Rixathon or Riximyo or Ruxience or Truxima) or (sorafenib or Nexavar) or (sunitinib or Sutent or su11248 or "su 11248") or (temsirolimus or Torisel) or (trametinib or Mekinist)) or ("phossy jaw*" or "bisphossy jaw*") or (ARONJ or BRONJ or BONJ or MRONJ)) and (extract* or remov* or exodont*))

Results: 44

Science Electronic Library Online Citation Index (SciELO) via Clarivate: 2002 to 2022-11-28

Website: https://www.webofscience.com/wos/scielo/

Search:

TS=(((jaw* or mandib* or maxill* or alveolar or dentoalveolar) and (osteonecro* or "bone necrosis" or osteochemonecro*) and ((agent* or anti-resorptive or antiresorptive or drug* or medication*) or (bisphosphon* or aminobisphosphon* or diphosphon*) or (alendron* or Binosto or Fosamax or Fosavance) or (clodron* or Bonefos or Clasteon or Clastoban or Ostac) or (etidron* or Didronel) or (ibandron* or Boniva or Bondenza or Bonviva or Bondronat or Iasibon) or (minodron* or Bonteo or Onobis or Recalbon or "ONO 5920" or ONO5920 or "YH 529" or YH529 or "YM 529" or YM529) or (neridron* or Nerixia) or (pamidron* or Aredia or Pamidria or Pamidonat or Pamifos or Pamisol) or (risedron* or Actonel or Atelvia) or (tiludron* or Skelid) or (zoledron* or Aclasta or Reclast or Zomera or Zometa) or (denosumab or Xgeva or Prolia) or (aflibercept or Eylea or Zaltrap) or (axitinib or Inlyta) or (bevacizumab or Abevmy or Alymsys or Avastin or Aybintio or Bambevi or Equidacent or Mvasi or Onbevzi or Oyavas or Zirabev) or (cabozantinib or Cabometyx or Cometriq or bms907351 or "bms 907351" or xl184 or "xl 184") or (dabrafenib or Tafinlar) or (dasatinib or Sprycel) or (erlotinib or Tarceva) or (everolimus or Afinitor or Certican or Votubia or Zortress or rad001 or "rad 001" or "sdzrad" or "sdz rad") or (imatinib or Gleevec or Glivec) or (ipilimumab or Yervoy) or (nivolumab or Opdivo) or (pazopanib or Votrient) or (rapamycin or sirolimus or Rapamune) or (regorafenib or Stivarga) or (rituximab or Blitzima or Mabthera or Riabni or Ritemvia or Rituxan or Rixathon or Riximyo or Ruxience or Truxima) or (sorafenib or Nexavar) or (sunitinib or Sutent or su11248 or "su 11248") or (temsirolimus or Torisel) or (trametinib or Mekinist)) or ("phossy jaw*" or "bisphossy jaw*") or (ARONJ or BRONJ or BONJ or MRONJ)) and (extract* or remov* or exodont*))

Results: 30

WHO Global Index Medicus (GIM): 2022-11-30

Website: https://www.globalindexmedicus.net/

Search:

(((mh:("Jaw" or "Jaw Diseases") or tw:(jaw* or mandib* or maxill* or alveolar or dentoalveolar)) and (mh:("Osteonecrosis" or "Bone Diseases, Infectious") or tw:(osteonecro* or "bone necrosis" or osteochemonecro*)) and (mh:("Bone Density Conservation Agents" or "Diphosphonates" or "Angiogenesis Inhibitors" or "Antineoplastic Agents") or tw:((agent* or anti-resorptive or antiresorptive or drug* or medication*) or (bisphosphon* or aminobisphosphon* or diphosphon*) or (alendron* or Binosto or Fosamax or Fosavance) or (clodron* or Bonefos or Clasteon or Clastoban or Ostac) or (etidron* or Didronel) or (ibandron* or Boniva or Bondenza or Bonviva or Bondronat or Iasibon) or (minodron* or Bonteo or Onobis or Recalbon

or "ONO 5920" or ONO5920 or "YH 529" or YH529 or "YM 529" or YM529) or (neridron* or Nerixia) or (pamidron* or Aredia or Pamidria or Pamidonat or Pamifos or Pamisol) or (risedron* or Actonel or Atelvia) or (tiludron* or Skelid) or (zoledron* or Aclasta or Reclast or Zomera or Zometa) or (denosumab or Xgeva or Prolia) or (aflibercept or Eylea or Zaltrap) or (axitinib or Inlyta) or (bevacizumab or Abevmy or Alymsys or Avastin or Aybintio or Bambevi or Equidacent or Mvasi or Onbevzi or Oyavas or Zirabev) or (cabozantinib or Cabometyx or Cometriq or bms907351 or "bms 907351" or xl184 or "xl 184") or (dabrafenib or Tafinlar) or (dasatinib or Sprycel) or (erlotinib or Tarceva) or (everolimus or Afinitor or Certican or Votubia or Zortress or rad001 or "rad 001" or "sdzrad" or "sdz rad") or (imatinib or Gleevec or Glivec) or (ipilimumab or Yervoy) or (nivolumab or Opdivo) or (pazopanib or Votrient) or (rapamycin or sirolimus or Rapamune) or (regorafenib or Stivarga) or (rituximab or Blitzima or Mabthera or Riabni or Ritemvia or Rituxan or Rixathon or Riximyo or Ruxience or Truxima) or (sorafenib or Nexavar) or (sunitinib or Sutent or su11248 or "su 11248") or (temsirolimus or Torisel) or (trametinib or Mekinist)))) or tw:((phossy jaw*) or (bisphossy jaw*) or ARONJ or BRONJ or BONJ or MRONJ) or mh:("Bisphosphonate-Associated Osteonecrosis of the Jaw")) and (mh:("Tooth Extraction") or tw:(extract* or remov* or exodont*))

Results: 109

4 from IMSEAR- the Index Medicus for the South-East Asia Region
53 from LILACS- the Latin America and the Caribbean Literature on Health Sciences
52 from WPRIM- the Western Pacific Region Index Medicus

WHO International Clinical Trials Registry Platform (ICTRP): Version 3.6
Website: https://trialsearch.who.int/
Search:
(jaw* or mandib* or maxill* or alveolar or dentoalveolar) and (osteonecro* or "bone necrosis" or osteochemonecro*)
Results: 76

ClinicalTrials.gov via United States (U.S.) National Library of Medicine (NIH): 2022-11-30
Website: https://clinicaltrials.gov/
Search:
Condition or disease: jaw and osteonecrosis
Results: 32

Characteristics and Risk-of-Bias Assessment of Included Reviews (5 studies with 6 reports)

Beth-Tasdogan 2022

**Methods**	**Study design**	Systematic review with meta-analysis
	**Study period**	Last search on 16 June 2021
	**Risk of bias assessment**	RoB1 tool (Cochrane Collaboration's tool for assessing Risk of Bias in randomized trial)
	**Registration**	Cochrane protocol (https://doi.org/10.1002/14651858.CD012432)
	**Funding source**	Internal sources: (1) Institute of Pharmacology of Natural Products & Clinical Pharmacology, and Institute of Epidemiology and Medical Biometry, Ulm University, Ulm, Germany; and (2) Oral Medicine, Diagnosis, and Periodontology Department, Faculty of Dentistry, Cairo University, Egypt.<br>External sources: (1) National Institute for Health Research (NIHR), UK; and (2) Cochrane Oral Health Global Alliance, Other.
**PICO equivalent**	**Patients/population**	Quote: "To assess preventive strategies, we included participants who were treated with known risk medications and who had not yet developed MRONJ before assignment to the experimental or control group."<br>"To assess interventions to treat MRONJ, we included people who had developed clinically apparent MRONJ. Case definition included exposure to risk drug and the presence of necrotic bone or fistulae that probes to bone."
	**Interventions**	Quote: "Any intervention (before or after commencement of antiresorptive or antiangiogenic drug therapy) that aims at prevention of MRONJ."<br>"Any intervention (non-surgical, surgical, or a combination of both) that aims to treat clinically manifest MRONJ."
	**Comparators**	Quote: "any single or combined experimental intervention versus control. The control arm consisted of participants receiving no treatment, placebo, or an active control (e.g. standard care)"
	**Outcomes**	(1) Prophylaxis of MRONJ: incidence of MRONJ, quality of life (QoL), time-to-event, rate of complications, and side effects of the intervention.<br>(2) Treatment of MRONJ: healing of MRONJ, QoL, recurrence, rate of complications, and side effects of the intervention.
**Search**	**Number of databases**	4 databases for published reports: (1) Cochrane Oral Health's Trials Register; (2) the Cochrane Central Register of Controlled Trials (CENTRAL); (3) Medline Ovid; and (4) Embase Ovid.<br>2 other sources for unpublished reports: (1) the US National Institutes of Health Trials Registry (ClinicalTrials.gov); and (2) the World Health Organization International Clinical Trials Registry Platform (ICTRP)
	**Search strategies**	Appendix 1 to 6 in the manuscript
**Results**	**Included studies**	13 randomized controlled trials (5 for pxrophylaxis of MRONJ and 8 for treatment of MRONJ)
	**Numerical results**	**Intervention:** dental extraction with PRGF<br>**Control:** dental extraction without PRGF<br>Incidence proportion of MRONJ<br>RR 0.08 (95% CI 0.00 to 1.51) from one RCT (Mozzati 2012)
		**Intervention:** sub-periosteal wound closure<br>**Control:** epi-periosteal wound closure<br>Incidence proportion of MRONJ<br>RR 0.09 (95% CI 0.00 to 1.56) from one RCT (Ristow 2021)
**Finding**	**GRADE**	Very low
	**Conclusion**	Quote: "There is insufficient evidence to either claim or refute a benefit of either of the interventions tested for prophylaxis of MRONJ (i.e. PRGF inserted into the postextraction alveolus during dental extractions, and wound closure by primary or secondary intention after dental extractions)."

Risk of Bias (ROBIS)

**Domain**	**Signalling questions**	**Response**	**Reviewers' judgement**	**Description**
**Concerns regarding specification of study eligibility criteria**	1.1	Y	Low	Comment: low risk of bias regarding specification of study eligibility criteria
	1.2	Y		
	1.3	Y		
	1.4	Y		
	1.5	Y		
**Concerns regarding methods used to identify and select studies**	2.1	Y	Low	Comment: low risk of bias regarding methods used to identify and select studies
	2.2	Y		
	2.3	Y		
	2.4	Y		
	2.5	Y		
**Concerns regarding methods used to collect data and appraise studies**	3.1	Y	Low	Comment: low risk of bias regarding methods used to collect data and appraise studies
	3.2	Y		
	3.3	Y		
	3.4	Y		
	3.5	Y		
**Concerns regarding the synthesis**	4.1	Y	High	Comment: the findings were not robust due to limited included studies.
	4.2	Y		
	4.3	Y		
	4.4	Y		
	4.5	N		
	4.6	Y		
**Bias of bias in the review**	A	Y	Low	
	B	Y		
	C	Y		

Abbreviations: Y = yes, PY = probably yes, N = no, PN = probably no, NI = no information, NA = not applicable.

References

[1] Beth-Tasdogan NH, Mayer B, Hussein H, Zolk O, Peter JU. Interventions for managing medication-related osteonecrosis of the jaw. Cochrane Database Syst Rev. 2022, 12;7(7):CD012432. [DOI: 10.1002/14651858.CD012432.pub3. PubMed: 35866376]

[2] Beth-Tasdogan NH, Mayer B, Hussein H, Zolk O. Interventions for managing medication-related osteonecrosis of the jaw. Cochrane Database Syst Rev. 2017, 10(10):CD012432. [DOI: 10.1002/14651858.CD012432.pub2; PubMed: 28983908]

Cabras 2021

**Methods**	**Study design**	Systematic review without meta-analysis
	**Study period**	February 2020 to May 2020
	**Risk of bias assessment**	QUIPS tool
	**Registration**	PROSPERO (CRD42020180061)
	**Funding source**	Not reported
**PICO equivalent**	**Patients/population**	Quote: "human patients treated with bisphosphonates/antiangiogenics/ antiresorptive agents undergoing dental extraction"
	**Interventions**	Quote: "any systemic antibiotic"
	**Comparators**	Quote: "no treatment, placebo or a different type of antibiotic"
	**Outcomes**	Quote: "subsequent development of MRONJ"
**Search**	**Number of databases**	2 databases for published reports: (1) Medline/PubMed; and (2) Scopus
	**Search strategies**	- "bisphosphonate osteonecrosis jaw and tooth extraction",<br>- "bisphosphonate osteonecrosis jaw and tooth extraction and antibiotics",<br>- "antiangiogenic and osteonecrosis and tooth extraction",<br>- "antiangiogenic and osteonecrosis and tooth extraction and antibiotics",<br>- "bevacizumab and osteonecrosis and tooth extraction",<br>- "bevacizumab and osteonecrosis and tooth extraction and antibiotics",<br>- "denosumab and osteonecrosis and tooth extraction",<br>- "denosumab and osteonecrosis and tooth extraction and antibiotics",<br>- "sunitinib and osteonecrosis and tooth extraction",<br>- "sunitinib and osteonecrosis and tooth extraction and antibiotics".
**Results**	**Included studies**	17 studies: 9 prospective studies, 4 retrospective studies, 3 case series, and 1 cohort study.
	**Numerical results**	None
**Finding**	**GRADE**	Not reported
	**Conclusion**	Quote: "In conclusion, empirical data acquired from case-series, prospective and retrospective studies suffering from a moderate/high risk of bias suggest that 2-3 g of amoxicillin daily, either alone or in combination with CP, for 6-7 days is the most-commonly deployed antibiotic treatment to minimize risk of MRONJ in patients under oral and intravenous bisphosphonates in need of dental extraction, with soft tissue closure techniques potentially providing further reduction of MRONJ risk. With only a small case-series of 19 patients under denosumab found, there is insufficient data to know if the aforesaid antibiotic protocol can be applied to patients exposed to the new generation of antiresorptive, bone-modifying agents."

Risk of Bias (ROBIS)

Domain	Signalling questions	Response	Reviewers' judgement	Description
**Concerns regarding specification of study eligibility criteria**	1.1	Y	High	Comment: non-English studies excluded
	1.2	Y		
	1.3	Y		
	1.4	Y		
	1.5	N		
**Concerns regarding methods used to identify and select studies**	2.1	N	High	Comment: only two databases (Medline and Scopus) searched for published reports
	2.2	Y		
	2.3	N		
	2.4	Y		
	2.5	Y		
**Concerns regarding methods used to collect data and appraise studies**	3.1	NI	Unclear	Comment: no information in process of data collection and risk of bias assessment
	3.2	Y		
	3.3	Y		
	3.4	PY		
	3.5	NI		
**Concerns regarding the synthesis**	4.1	PN	High	Comment: meta-analysis could have been undertaken but not.
	4.2	PN		
	4.3	PN		
	4.4	NI		
	4.5	NI		
	4.6	NI		
**Bias of bias in the review**	A	N	High	
	B	Y		
	C	Y		

Abbreviations: Y = yes, PY = probably yes, N = no, PN = probably no, NI = no information, NA = not applicable.

References

Cabras M, Gambino A, Broccoletti R, Sciascia S, Arduino P G. Lack of evidence in reducing risk of MRONJ after teeth extractions with systemic antibiotics. J Oral Sci, 2021, 63(3): 217-226. [DOI: 10.2334/josnusd.21-0016; PubMed: 34193777]

Del Fabbro 2015

**Methods**	**Study design**	Systematic review with meta-analysis
	**Study period**	January 2014 to September 2020
	**Risk of bias assessment**	Methodological parameters customized by review authors
	**Registration**	Not reported
	**Funding source**	Not reported
**PICO equivalent**	**Patients/population**	Not reported
	**Interventions**	APC, including PRF, PRGF and PRP
	**Comparators**	Not reported
	**Outcomes**	Quote: "For being included, studies had to report clinical results of oral surgery procedures in patients under bisphosphonate therapy, in which autologous platelet concentrate was used for improving clinical outcome. Both articles reporting on the treatment of an existing condition of BRONJ (such as surgical resection of the necrotic tissue), and studies reporting on the incidence/ onset of BRONJ in patients undergoing oral surgery procedures (such as tooth extraction, dental implant placement) were considered."
**Search**	**Number of databases**	3 databases for published reports: (1) Medline; (2) Scopus; and (3) Cochrane Central Register of Controlled Trials (CENTRAL)
	**Search strategies**	Quote: "The search terms used were: 'bisphosphonate*', 'BRONJ', 'osteonecrosis', 'maxilla', 'mandible', 'platelet-rich plasma', 'platelet concentrates', 'platelet growth factors', 'platelet-rich fibrin', 'PRP', 'PRGF', 'PRF', 'oral surgery', 'extraction socket', 'tooth extraction'. They were used alone or in combination using Boolean operators OR and AND. Furthermore, a hand search of issues from 2000 up to the last issue available on 15th January 2014, including the 'Early view' (or equivalent) section was undertaken on the following journals: British Journal of Oral and Maxillofacial Surgery, International Journal of Oral and Maxillofacial Surgery, Journal of Oral and Maxillofacial Surgery, Oral Oncology, Oral Surgery, Oral Medicine, Oral Pathology, Oral Radiology and Endodontology. The reference list of the retrieved reviews and of the included studies was also searched for possible additional eligible studies not identified by the electronic search."
**Results**	**Included studies**	18 studies.
	**Numerical results**	**Intervention:** dental extraction with PRGF<br>**Control:** dental extraction without PRGF<br>Incidence proportion of BRONJ<br>OR 0.08 (95% CI 0.00 to 1.47) from one RCT (Mozzati 2012)
**Finding**	**GRADE**	Not reported
	**Conclusion**	Quote: "In conclusion, the results of this review, though based on low-evidence level studies, suggest that the use of platelet concentrates as an adjunct to oral surgery procedures may have a beneficial effect for preventing the postsurgical occurrence or recurrence of BRONJ in patients under bisphosphonate therapy. We hope that this finding might stimulate researchers to performing further case–control studies and possibly randomised studies with large sample sizes in order to confirm the beneficial effects of platelet concentrates in the prevention and management of BRONJ."

Risk of Bias (ROBIS)

Domain	Signalling questions	Response	Reviewers' judgement	Description
**Concerns regarding specification of study eligibility criteria**	1.1	NI	Unclear	Comment: no protocol registration reported
	1.2	Y		
	1.3	Y		
	1.4	Y		
	1.5	Y		
**Concerns regarding methods used to identify and select studies**	2.1	N	High	Comment: only three databases (Medline, Scopus and CENTRAL) searched for published reports
	2.2	Y		
	2.3	PN		
	2.4	Y		
	2.5	Y		
**Concerns regarding methods used to collect data and appraise studies**	3.1	Y	High	Comment: high risk of bias in study appraisal
	3.2	Y		
	3.3	Y		
	3.4	N		
	3.5	Y		
**Concerns regarding the synthesis**	4.1	PN	High	Comment: the findings were not robust due to limited included studies.
	4.2	NI		
	4.3	PN		
	4.4	NI		
	4.5	N		
	4.6	NI		
**Bias of bias in the review**	A	N	High	
	B	Y		
	C	Y		

Abbreviations: Y = yes, PY = probably yes, N = no, PN = probably no, NI = no information, NA = not applicable.

References

Del Fabbro M, Gallesio G, Mozzati M. Autologous platelet concentrates for bisphosphonate-related osteonecrosis of the jaw treatment and prevention. A systematic review of the literature. Eur J Cancer, 2015, 51(1): 62-74. [DOI: 10.1016/j.ejca.2014.10.015; PubMed: 25466505]

Ottesen 2020

**Methods**	**Study design**	Systematic review without meta-analysis
	**Study period**	April 2018 to April 2019
	**Risk of bias assessment**	Newcastle–Ottawa Scale (NOS)
	**Registration**	PROSPERO (CRD42018103124)
	**Funding source**	None
**PICO equivalent**	**Patients/population**	Quote: "Adults with malignant bone disease undergoing high-dose AR therapy."
	**Interventions**	Quote: "Discontinuation (i.e., drug holiday) of high-dose AR therapy at the time of tooth extraction or dentoalveolar surgery."
	**Comparators**	Quote: "Continuation (i.e., no drug holiday) of high-dose AR therapy at the time of (prior to and/or after) tooth extraction or dentoalveolar surgery."
	**Outcomes**	Quote: "Primary outcome is development of MRONJ (þ/-) and thereafter divided into the 4 stages of MRONJ defined by AAOMS."
**Search**	**Number of databases**	3 databases for published reports: (1) Medline/PubMed; (2) Embase; and (3) Cochrane Central Register of Controlled Trials (CENTRAL).
	**Search strategies**	(((((((malignant bone disease) OR (breast neoplasms OR breast tumor OR breast tumors OR breast cancer)) OR (prostate cancer OR metastatic prostate cancer OR prostatic neoplasms OR prostatic cancer)) OR (myelomatosis OR multiple myelomas OR multiple myeloma OR myelomatosis))) AND ((((((antiresorptive drug holiday) OR (antiresorptive agents OR antiresorptive agent OR antiresorptive drugs OR antiresorptive drug)) OR (diphosphonates OR bisphosphonates OR bisphosphonate)) OR bone density conservation agents) OR (alendronate OR zometa OR fosamax OR pamifos OR xgeva OR zoledronic acid OR denosumab[all])) OR (discontinue OR break OR suspension OR interruption OR cessation OR time out))) AND (tooth extraction OR tooth extractions OR extraction OR extractions OR oral surgical procedures OR alveolectomy)) AND (bisphosphonate-associated osteonecrosis ofthe jaw OR osteonecrosis OR jaw OR jaws OR ONJ OR medication related osteonecrosis of the jaw OR osteonecrosis of the jaw OR dead jaw bone OR bisphosphonate-related osteonecrosis of the jaw)
**Results**	**Included studies**	14 studies: 3 prospective studies, and 11 retrospective studies.
	**Numerical results**	None
**Finding**	**GRADE**	Not reported
	**Conclusion**	Quote: "The efficacy of a high-dose AR drug holiday remains uncertain."

Risk of Bias (ROBIS)

Domain	Signalling questions	Response	Reviewers' judgement	Description
**Concerns regarding specification of study eligibility criteria**	1.1	Y	Low	Comment: low risk of bias regarding specification of study eligibility criteria
	1.2	Y		
	1.3	Y		
	1.4	Y		
	1.5	Y		
**Concerns regarding methods used to identify and select studies**	2.1	N	High	Comment: only three databases (Medline, Embase and Central) searched for published reports, with restriction in English.
	2.2	Y		
	2.3	Y		
	2.4	N		
	2.5	Y		
**Concerns regarding methods used to collect data and appraise studies**	3.1	NI	Unclear	Comment: no information in process of data collection and risk of bias assessment
	3.2	Y		
	3.3	Y		
	3.4	PY		
	3.5	NI		
**Concerns regarding the synthesis**	4.1	PN	High	Comment: meta-analysis could have been undertaken but not.
	4.2	PN		
	4.3	PN		
	4.4	NI		
	4.5	NI		
	4.6	NI		
**Bias of bias in the review**	A	N	High	
	B	Y		
	C	Y		

Abbreviations: Y = yes, PY = probably yes, N = no, PN = probably no, NI = no information, NA = not applicable.

References

Ottesen C, Schiodt M, Gotfredsen K. Efficacy of a high-dose antiresorptive drug holiday to reduce the risk of medication-related osteonecrosis of the jaw (MRONJ): A systematic review. Heliyon, 2020, 6(4): e03795. [DOI: 10.1016/j.heliyon.2020.e03795; PubMed: 32373730]

Sacco 2021

**Methods**	**Study design**	Umbrella review (overview of reviews)
	**Study period**	April 2018 to April 2019
	**Risk of bias assessment**	GRADE-CERQual
	**Registration**	INPLASY (PLASY202160061)
	**Funding source**	None
**PICO equivalent**	**Patients/population**	Quote: "any (no limits of age) patients with MRONJ"
	**Interventions**	Quote: "any types"
	**Comparators**	Quote: "any types"
	**Outcomes**	Quote: "state of knowledge based on the type of studies included in the reviews"
**Search**	**Number of databases**	4 databases for published reports: (1) Medline; (2) PubMed; (3) Embase; and (4) CINAHL.<br>3 other sources for unpublished reports: (1) PROSPERO; (2) INPLASY; and (3) OFS
	**Search strategies**	1. Osteonecrosis [MeSH Terms] ORAvascular osteonecrosis of the jaw [MeSH Terms] OR Osteonecrosis of the jaw [MeSH Terms] OR MRONJ [MeSH Terms] OR ONJ [MeSH Terms] OR BONJ [MeSH Terms] OR ARONJ [MeSH Terms] OR BRONJ Patients [MeSH Terms] OR Any patients [MeSH Terms] OR Oncology [MeSH Terms] OR Osteoporosis [MeSH Terms] OR Non-oncologic patients;<br>2. Systematic review [MeSH Terms] OR Review [MeSH Terms] OR Meta-analysis;<br>3. 1 and 2 and 3.
**Results**	**Included studies**	25 studies: 4 systematic reviews with meta-analysis, and 21 systematic reviews without meta-analysis.
	**Numerical results**	None
**Finding**	**GRADE**	Very low (GRADE-CERQual)
	**Conclusion**	Quote: "Through this umbrella review, it has become clear that there is limited high strength evidence to support many of the current recommendations surrounding medication-related osteonecrosis of the jaw. The low quality systematic reviews and meta-analyses highlighted by this study show no insightful therapeutic recommendations, preventive strategies, risk reduction or standards that can be applied for this debilitating disease."

Risk of Bias (ROBIS)

Domain	Signalling questions	Response	Reviewers' judgement	Description
**Concerns regarding specification of study eligibility criteria**	1.1	Y	Low	Comment: low risk of bias regarding specification of study eligibility criteria
	1.2	Y		
	1.3	Y		
	1.4	Y		
	1.5	Y		
**Concerns regarding methods used to identify and select studies**	2.1	Y	Low	Comment: low risk of bias regarding methods used to identify and select studies
	2.2	Y		
	2.3	Y		
	2.4	Y		
	2.5	Y		
**Concerns regarding methods used to collect data and appraise studies**	3.1	Y	High	Comment: GRADE-CERQual tool was used to assess not the risk of bias but the confidence of findings in reviews
	3.2	PY		
	3.3	PY		
	3.4	N		
	3.5	PY		
**Concerns regarding the synthesis**	4.1	PY	High	Comment: risk of bias in reviews not addressed completely
	4.2	PY		
	4.3	NI		
	4.4	NI		
	4.5	NI		
	4.6	N		
**Bias of bias in the review**	A	N	High	
	B	Y		
	C	Y		

Abbreviations: Y = yes, PY = probably yes, N = no, PN = probably no, NI = no information, NA = not applicable.

References

Sacco R, Calasans-Maia M D, Woolley J, Akintola O, de Almeida Barros Mourão C F, Moraschini V, Kushnerev E, Acocella A, Obisesan O, Yates J. 18 years of medication-related osteonecrosis of the jaw (Mronj) research: Where are we now?—an umbrella review. Appl Sci, 2021, 11(19): 8818. [DOI: 10.3390/app11198818]

Characteristics and Risk-of-Bias Assessment of Included Primary Studies (15 studies with 21 reports)

Asaka 2017

**Methods**	**Study design**	Historical controlled trial with one prospective and one retrospective group
	**Study period**	July 2013 to March 2015 (prospective);<br>July 2006 to August 2010 (retrospective)
	**Location**	Sapporo, Japan
	**Number of centers**	1
	**Setting**	Department of Oral Medicine, Hokkaido University Hospital
	**Funding source**	KAKENHI Grant-in Aid for Scientific Research (B) (26861695)
**Participants**	**Inclusion criteria**	Patients undergoing oral bisphosphonate therapy for osteoporosis or glucocorticoid-induced osteoporosis for over 1 year and requiring tooth extraction
	**Exclusion criteria**	Patients who had received high-dose steroid therapy (daily 50 mg and more), with active local infections, with previous history of irradiation to the maxillofacial area, with poor general condition, and exhibiting neoplastic involvement of the jaw
	**Number allocated**	**102 patients:** 9 males/93 females, median age 69 ranged from 24 to 88<br>**Systemic conditions:** osteoporosis (68 patients), rheumatoid arthritis (19 patients), systemic lupus erythematosus (6 patients), and other autoimmune diseases (9 patients)<br>**Drugs:** alendronate (53 patients), risedronate (49 patients), minodronate (12 patients), and etidronate (5 patients) (There is some overlapping.)
	**Number withdrawn**	0
	**Number evaluated**	102 patients (218 extractions)
**Interventions**	**Comparison 1**	**APC (Autologous platelet concentrates) versus control**<br>**Group 1.1 (prospective): 29 patients (52 extractions)**<br>Use of **PRF (platelet-rich fibrin)** laid directly over the bone to fill the tooth socket;<br>Standard antibiotics (either amoxicillin 250 mg every 8 hours or clindamycin 150 mg every 6 hours) for 1 week, starting from the morning of the surgery.<br>**Group 1.2 (retrospective): 73 patients (166 extractions)**<br>No use of PRF;<br>Nonstandard antibiotics (only 28 patients received)
	**Comparison 2**	**Drug holiday versus drug continuation**<br>**Group 2.1: 76 patients (25 prospective and 51 retrospective)**<br>Drug holiday for 3 months before extraction<br>**Group 2.2: 26 patients (4 prospective and 22 retrospective)**<br>Drug continuation
	**Other**	Quote: “An alveolar nerve block infiltration and local anesthesia was administered using 2% lidocaine with 1:80,000 epinephrine, depending on the dental site. Delicate tooth extraction and curettage was performed with or without the elevation of full-thickness flaps.”<br>“No other material (such as oxidized cellulose or collagen sponge) was inserted into the socket in either group.”
	**Operators**	Experienced oral surgeons, number of whom was not reported
**Outcomes**	**Outcomes**	Incidence of delayed healing at 1 weeks, 2 weeks and 4 weeks (1 month)<br>Incidence of MRONJ at 8 weeks (2 months) and 12 weeks (3 months)
**Notes**	**Sample size calculation**	Not reported
	**Baseline comparability**	Quote: “Gender, teeth extracted site, extracted teeth type, age, systemic pathology (osteoporosis or glucocorticoid-induced osteoporosis), type of BP, other risk factors (steroids, immunosuppressant, and diabetes), duration of BP therapy, and period of discontinuation of oral BP are listed in **Table 1**. There were no significant differences between the two groups with regard to any of these factors except for duration of BP therapy.”

Risk of Bias (ROBINS-I)

Domain	Signalling questions	Response	Reviewers' judgement	Description
**Bias due to confounding**	1.1	Y	Serious	Comment: use of antibiotics as a confounding factor was recorded but excluded from analysis.
	1.2	N		
	1.3	NA		
	1.4	N		
	1.5	NA		
	1.6	N		
	1.7	NA		
	1.8	NA		
**Bias in selection of participants into the study**	2.1	N	Low	Comment: low risk of bias in selection of participants into the historical controlled trial
	2.2	NA		
	2.3	NA		
	2.4	Y		
	2.5	NA		
**Bias in classification of interventions**	3.1	Y	Low	Comment: low risk of bias in classification of interventions
	3.2	Y		
	3.3	N		
**Bias due to deviations from intended interventions (effect of assignment to intervention)**	4.1	NI	No Information	Comment: no information about deviation from intended interventions
	4.2	NA		
**Bias due to missing data**	5.1	Y	Low	Comment: no withdrawal
	5.2	N		
	5.3	N		
	5.4	NA		
	5.5	NA		
**Bias in measurement of outcomes**	6.1	Y	Serious	Comment: no blinding of outcome assessment
	6.2	Y		
	6.3	Y		
	6.4	N		
**Bias in selection of the reported result**	7.1	PN	Moderate	Comment: no preregistered protocol available, nor indication of selection of reported result
	7.2	PN		
	7.3	PN		
**Overall bias**	-	-	Serious	

Abbreviations: Y = yes, PY = probably yes, N = no, PN = probably no, NI = no information, NA = not applicable.

References

Asaka T, Ohga N, Yamazaki Y, Sato J, Satoh C, Kitagawa Y. Platelet-rich fibrin may reduce the risk of delayed recovery in tooth-extracted patients undergoing oral bisphosphonate therapy: a trial study. Clinical Oral Investigations 2017;21(7):2165-2172. [CENTRAL: CN-01642243; DOI: 10.1007/s00784-016-2004-z; EMBASE: 623758644; PubMed: 27837344]

Bodem 2015

**Methods**	**Study design**	Prospective cohort study
	**Study period**	Not reported
	**Location**	Germany
	**Number of centers**	1
	**Setting**	Not reported
	**Funding source**	None
**Participants**	**Inclusion criteria**	Patients with either ongoing or completed therapy with intravenous bisphosphonates due to a malignant disease
	**Exclusion criteria**	Radiation therapy in the medical history; clinical or radiological evidence of BRONJ Stage 0-III according to the AAOMS.
	**Number allocated**	**61 patients:** 19 males/42 females, mean age 65.65 ± 12.69 ranged from 34 to 87<br>**Systemic conditions:** malignant diseases, including breast cancer, multiple myeloma, prostatic cancer, renal cell carcinoma, urothelial carcinoma and rectal carcinoma.<br>**Drugs:** zoledronic acid (38 patients), ibandronate (17 patients), and pamidronate (6 patients)
	**Number withdrawn**	0
	**Number evaluated**	61 patients (184 extractions)
**Interventions**	**Comparison 1**	**Drug holiday versus drug continuation**<br>**Group 1.1: 17 patients**<br>Drug holiday (bisphosphonates paused/completed) for an average of 17.6 ± 15.9 months (ranged from 1 to 63 months) before extraction<br>**Group 1.2: 44 patients**<br>Drug continuation (bisphosphonates ongoing)
	**Comparison 2**	**Gastric feeding tube versus control**<br>**Group 2.1: 26 patients**<br>Accepting a gastric feeding tube for an average of 2.1 ± 2.7 days (ranged from 0 to 11 days)<br>**Group 2.2: 35 patients**<br>Refusing a gastric feeding tube
	**Other**	Quote: "All patients were treated using a standardized surgical extraction protocol under local or general anaesthesia depending on the number of extracted teeth, the general health status of the patient and patient's compliance. Surgical tooth extraction was performed as follows: (I) elevation of a mucosal flap by epiperiosteal preparation with bilateral release incisions, if necessary; (II) extraction of tooth; and (III) tension-free closure of the alveolar socket. If tooth extraction was not possible using forceps only, an additional osteotomy was performed including removal of facial and/or lingual/palatine bone if necessary."<br>"The perioperative adjuvant treatment included intravenous antibiotic prophylaxis (1.5 g of ampicillin-sulbactam 3 times per day) starting at least 24 h before surgical treatment and continued postoperatively for a recommended period of 5 days. Patients with known allergy to penicillin were given 600mg clindamycin, 3 times per day. Furthermore, for optimizing oral hygiene, we recommended a gastric feeding tube to the patients and a mouth rinse with antimicrobiological solution (chlorhexidine 0.12%) three times a day."
	**Operators**	Not reported
**Outcomes**	**Outcomes**	Incidence of MRONJ at 12 weeks (3 months)
**Notes**	**Sample size calculation**	Not reported
	**Baseline comparability**	Not reported

Risk of Bias (ROBINS-I)

**Domain**	**Signalling questions**	**Response**	**Reviewers' judgement**	**Description**
**Bias due to confounding**	1.1	Y	Serious	Comment: baseline confounding factors not controlled
	1.2	N		
	1.3	NA		
	1.4	N		
	1.5	NA		
	1.6	N		
	1.7	NA		
	1.8	NA		
**Bias in selection of participants into the study**	2.1	N	Low	Comment: low risk of bias in selection of participants into the prospective cohort study
	2.2	NA		
	2.3	NA		
	2.4	Y		
	2.5	NA		
**Bias in classification of interventions**	3.1	Y	Low	Comment: low risk of bias in classification of interventions
	3.2	Y		
	3.3	N		
**Bias due to deviations from intended interventions (effect of assignment to intervention)**	4.1	NI	No information	Comment: no information about deviation from intended interventions
	4.2	NA		
**Bias due to missing data**	5.1	Y	Low	Comment: no withdrawal
	5.2	N		
	5.3	N		
	5.4	NA		
	5.5	NA		
**Bias in measurement of outcomes**	6.1	Y	Serious	Comment: no information about blinding of outcome assessment
	6.2	NI		
	6.3	Y		
	6.4	N		
**Bias in selection of the reported result**	7.1	PN	Moderate	Comment: no preregistered protocol available, nor indication of selection of reported result
	7.2	PN		
	7.3	PN		
**Overall bias**	-	-	Serious	

Abbreviations: Y = yes, PY = probably yes, N = no, PN = probably no, NI = no information, NA = not applicable.

References

Bodem JP, Kargus S, Eckstein S, Saure D, Engel M, Hoffmann J, Freudlsperger C. Incidence of bisphosphonate-related osteonecrosis of the jaw in high-risk patients undergoing surgical tooth extraction. Journal of Cranio-Maxillo-Facial Surgery 2015; 43(4):510-514. [DOI: 10.1016/j.jcms.2015.02.018; EMBASE: 603508329; PubMed: 25841311]

Hasegawa 2017

**Methods**	**Study design**	Multicenter retrospective cohort study
	**Study period**	January 2008 to December 2015
	**Location**	Japan
	**Number of centers**	9
	**Setting**	Nine institutions belonging to the Japanese Study Group of Co-operative Dentistry with Medicine (JCDM): (1) Department of Oral and Maxillofacial Surgery, Kobe University Graduate School of Medicine; (2) Department of Clinical Oral Oncology, Nagasaki University Graduate School of Biomedical Sciences; (3) Department of Oral and Maxillofacial Surgery, Nara Medical University; (4) Department of Oral and Maxillofacial Surgery, Kakogawa Central City Hospital; (5) Department of Oral and Maxillofacial Surgery, Shin-Suma General Hospital; (6) Department of Dentistry and Oral Surgery, Shinshu University School of Medicine; (7) Department of Oral and Maxillofacial Surgery, Kobe Central Hospital; (8) Department of Dentistry and Oral Surgery, Kansai Medical University; and (9) Nagoya City University Graduate School of Medical Sciences.
	**Funding source**	None
**Participants**	**Inclusion criteria**	(1) Patients receiving oral bisphosphonate therapy; (2) tooth extraction
	**Exclusion criteria**	Patients receiving intravenous BPs, denosumab, antiangiogenic agents, or intramuscular agents
	**Number allocated**	**1175 patients (2458 extractions):** 161 males/1014 females, mean age 70.7 ± 11.7 ranged from 23 to 102<br>**Systemic conditions:** osteoporosis (943 patients), rheumatism (110 patients), diabetes mellitus (102 patients), cancer (61 patients), renal insufficiency including dialysis (6 patients) and others (321 patients) (There is some overlapping.)<br>**Drugs:** alendronate (742 patients), risedronate (334 patients), minodronate (129 patients), others (10 patients) and unknown (11 patients) (There is some overlapping.)
	**Number withdrawn**	0
	**Number evaluated**	1175 patients (2457 extractions in Comparison 1, and 2430 extractions in Comparison 2)
**Interventions**	**Comparison 1**	**Drug holiday versus drug continuation**<br>**Group 1.1: 1818 extractions**<br>Drug holiday for more than 2 months before extraction<br>**Group 1.2: 639 extractions**<br>Drug continuation
	**Comparison 2**	**Secondary healing with wound open versus secondary healing with wound closure versus primary healing with wound complete closure**<br>**Group 2.1: 855 extractions**<br>Secondary healing with wound open<br>**Group 2.2: 1470 extractions**<br>Secondary healing with wound closed with suture<br>**Group 2.3: 105 extractions**<br>Primary healing with wound completely closed with relaxation incision or removal of bone
	**Other**	Not applicable
	**Operators**	Not reported
**Outcomes**	**Outcomes**	Incidence of MRONJ (average duration until diagnosis was 9.5 ± 4.2 weeks)
**Notes**	**Sample size calculation**	Not reported
	**Baseline comparability**	“Table 1 Characteristics and demographics of patients receiving oral BP” and “Table 2 Characteristics and incidence rates of MRONJ” in the manuscript

Risk of Bias (ROBINS-I)

**Domain**	**Signalling questions**	**Response**	**Reviewers' judgement**	**Description**

**Bias due to confounding**	1.1	Y	Moderate	Comment: multivariate logistic regression analysis with 95% confidence intervals.
	1.2	N		
	1.3	NA		
	1.4	Y		
	1.5	Y		
	1.6	N		
	1.7	NA		
	1.8	NA		
**Bias in selection of participants into the study**	2.1	N	Low	Comment: low risk of bias in selection of participants into the retrospective cohort study
	2.2	NA		
	2.3	NA		
	2.4	Y		
	2.5	NA		
**Bias in classification of interventions**	3.1	Y	Moderate	Comment: potential slight recall bias in the retrospective cohort study
	3.2	N		
	3.3	N		
**Bias due to deviations from intended interventions (effect of assignment to intervention)**	4.1	NI	No Information	Comment: no information about deviation from intended interventions
	4.2	NA		
**Bias due to missing data**	5.1	Y	Low	Comment: 1 extraction (0.04%) excluded from analysis in Comparison 1, and 28 extractions (1.14%) in Comparison 2
	5.2	Y		
	5.3	N		
	5.4	NI		
	5.5	Y		
**Bias in measurement of outcomes**	6.1	Y	Serious	Comment: no information about blinding of outcome assessment
	6.2	NI		
	6.3	Y		
	6.4	N		
**Bias in selection of the reported result**	7.1	PN	Moderate	Comment: no preregistered protocol available, nor indication of selection of reported result
	7.2	PN		
	7.3	PN		
**Overall bias**	-	-	Serious	

Abbreviations: Y = yes, PY = probably yes, N = no, PN = probably no, NI = no information, NA = not applicable.

References

[1] Hasegawa T, Kawakita A, Ueda N, Funahara R, Tachibana A, Kobayashi M, Kondou E, Takeda D, Kojima Y, Sato S, Yanamoto S, Komatsubara H, Umeda M, Kirita T, Kurita H, Shibuya Y, Komori T, Japanese Study Group of Cooperative Dentistry with Medicine (JCDM). A multicenter retrospective study of the risk factors associated with medication-related osteonecrosis of the jaw after tooth extraction in patients receiving oral bisphosphonate therapy: can primary wound closure and a drug holiday really prevent MRONJ? Osteoporosis International 2017;28(8):2465-2473. [DOI: 10.1007/s00198-017-4063-7; EMBASE: 615772946; PubMed: 28451732]

[2] Hasegawa T, Ri S, Umeda M, Komatsubara H, Kobayashi M, Shigeta T, Yoshitomi I, Ikeda H, Shibuya Y, Asahina I, Komori T. The observational study of delayed wound healing after tooth extraction in patients receiving oral bisphosphonate therapy. Journal of Cranio-Maxillo-Facial Surgery 2013;41(7):558-563. [DOI: 10.1016/j.jcms.2012.11.023; EMBASE: 52396994; PubMed: 23332469]

Hasegawa 2019

**Methods**	**Study design**	Multicenter retrospective cohort study
	**Study period**	January 2008 to December 2016
	**Location**	Japan
	**Number of centers**	10
	**Setting**	Ten institutions belonging to the Japanese Study Group of Co-operative Dentistry with Medicine (JCDM): (1) Department of Oral and Maxillofacial Surgery, Kobe University Graduate School of Medicine; (2) Department of Clinical Oral Oncology, Nagasaki University Graduate School of Biomedical Sciences; (3) Department of Dentistry and Oral Surgery, Shinshu University School of Medicine; (4) Department of Oral and Maxillofacial Surgery, Wakayama Medical University; (5) Department of Oral and Maxillofacial Surgery, Nagoya City University Graduate School of Medical Sciences; (6) Department of Dentistry and Oral Surgery, Kansai Medical University; (7) Department of Oral and Maxillofacial Surgery, Nara Medical University; (8) Department of Oral and Maxillofacial Surgery, Kakogawa Central City Hospital; (9) Department of Oral and Maxillofacial Surgery, Osaka City University Graduate School of Medicine; and (10) Department of Oral and Maxillofacial Surgery, Shin-Suma General Hospital.
	**Funding source**	Not reported
**Participants**	**Inclusion criteria**	Patients receiving high-dose BMA (intravenous BP or Dmab) for cancer; (2) tooth extraction
	**Exclusion criteria**	Patients receiving intravenous BP or Dmab therapy for osteoporosis
	**Number allocated**	**85 patients (163 extractions):** 34 males/51 females, mean age 64.5 ± 11.5 ranged from 39 to 90<br>**Systemic conditions:** cancer (all 85 patients)<br>**Drugs:** zoledronate (52 patients), alendronate (1 patient), risedronate (1 patient), and denosumab (39 patients) (There is some overlapping.)
	**Number withdrawn**	0
	**Number evaluated**	85 patients (163 extractions in Comparison 1, and 161 extractions in Comparison 2)
**Interventions**	**Comparison 1**	**Drug holiday versus drug continuation**<br>**Group 1.1: 58 extractions**<br>Drug holiday for more than 2 months before extraction<br>**Group 1.2: 105 extractions**<br>Drug continuation
	**Comparison 2**	**Secondary healing with wound open versus secondary healing with wound closure versus primary healing with wound complete closure**<br>**Group 2.1: 59 extractions**<br>Secondary healing with wound open<br>**Group 2.2: 85 extractions**<br>Secondary healing with wound closed with suture<br>**Group 2.3: 17 extractions**<br>Primary healing with wound completely closed with relaxation incision or removal of bone
	**Other**	Not applicable
	**Operators**	Not reported
**Outcomes**	**Outcomes**	Incidence of MRONJ (follow-up duration not reported)
**Notes**	**Sample size calculation**	Not reported
	**Baseline comparability**	"Table 2 Characteristics of teeth according to the presence of MRONJ" in the manuscript

Risk of Bias (ROBINS-I)

Domain	Signalling questions	Response	Reviewers' judgement	Description
**Bias due to confounding**	1.1	Y	Moderate	Comment: multivariate logistic regression analysis with 95% confidence intervals.
	1.2	N		
	1.3	NA		
	1.4	Y		
	1.5	Y		
	1.6	N		
	1.7	NA		
	1.8	NA		
**Bias in selection of participants into the study**	2.1	N	Low	Comment: low risk of bias in selection of participants into the retrospective cohort study
	2.2	NA		
	2.3	NA		
	2.4	Y		
	2.5	NA		
**Bias in classification of interventions**	3.1	Y	Moderate	Comment: potential slight recall bias in the retrospective cohort study
	3.2	N		
	3.3	N		
**Bias due to deviations from intended interventions (effect of assignment to intervention)**	4.1	NI	No Information	Comment: no information about deviation from intended interventions
	4.2	NA		
**Bias due to missing data**	5.1	Y	Low	Comment: no withdrawal in Comparison 1, and 2 extractions (1.23%) excluded from analysis in Comparison 2
	5.2	Y		
	5.3	N		
	5.4	NI		
	5.5	Y		
**Bias in measurement of outcomes**	6.1	Y	Serious	Comment: no information about blinding of outcome assessment
	6.2	NI		
	6.3	Y		
	6.4	N		
**Bias in selection of the reported result**	7.1	PN	Moderate	Comment: no preregistered protocol available, nor indication of selection of reported result
	7.2	PN		
	7.3	PN		
**Overall bias**	-	-	Serious	

Abbreviations: Y = yes, PY = probably yes, N = no, PN = probably no, NI = no information, NA = not applicable.

References

Hasegawa T, Hayashida S, Kondo E, Takeda Y, Miyamoto H, Kawaoka Y, Ueda N, Iwata E, Nakahara H, Kobayashi M, Soutome S, Yamada SI, Tojyo I, Kojima Y, Umeda M, Fujita S, Kurita H, Shibuya Y, Kirita T, Komori T; Japanese Study Group of Co-operative Dentistry with Medicine (JCDM). Medication-related osteonecrosis of the jaw after tooth extraction in cancer patients: a multicenter retrospective study. Osteoporosis International 2019;30(1):231-239. [DOI: 10.1007/s00198-018-4746-8; EMBASE: 624773912; PubMed: 30406309]

Hasegawa 2021

**Methods**	**Study design**	Multicenter retrospective cohort study
	**Study period**	January 2008 to December 2019
	**Location**	Japan
	**Number of centers**	10
	**Setting**	Ten institutions belonging to the Japanese Study Group of Co-operative Dentistry with Medicine (JCDM)
	**Funding source**	Not reported
**Participants**	**Inclusion criteria**	(1) Patients with cancer and receiving an oncologic (120 mg) dose of denosumab once a month; (2) tooth extraction
	**Exclusion criteria**	Patients receiving denosumab for osteoporosis
	**Number allocated**	**72 patients (136 extractions):** 31 males/41 females, mean age 65.2 ± 11.8 ranged from 41 to 85<br>**Systemic conditions:** breast cancer (20 patients), prostate cancer (14 patients), lung cancer (8 patients), multiple myeloma (5 patients), other cancers (5 patients), and unknown (20 patients) (There is some overlapping.)<br>**Drugs:** denosumab (all 72 patients)
	**Number withdrawn**	0
	**Number evaluated**	72 patients (136 extractions)
**Interventions**	**Comparison 1**	**Drug holiday versus drug continuation**<br>**Group 1.1: 72 extractions**<br>Drug holiday for more than 1 month (30 days) before extraction<br>**Group 1.2: 64 extractions**<br>Drug continuation
	**Comparison 2**	**Secondary healing with wound open versus secondary healing with wound closure versus primary healing with wound complete closure**<br>**Group 2.1: 27 patients (50 extractions)**<br>Secondary healing with wound open<br>**Group 2.2: 40 patients (71 extractions)**<br>Secondary healing with wound closed with suture<br>**Group 2.3: 5 patients (15 extractions)**<br>Primary healing with wound completely closed with relaxation incision or removal of bone
	**Other**	Not applicable
	**Operators**	Not reported
**Outcomes**	**Outcomes**	Incidence of MRONJ (follow-up duration not reported)
**Notes**	**Sample size calculation**	Not reported
	**Baseline comparability**	"Table 1 Characteristics of patients according to whether or not denosumab-related osteonecrosis of the jaw was present" and "Table 2 Characteristics of extracted teeth according to whether or not denosumab-related osteonecrosis of the jaw was present" in the manuscript

Risk of Bias (ROBINS-I)

Domain	Signalling questions	Response	Reviewers' judgement	Description
**Bias due to confounding**	1.1	Y	Moderate	Comment: multivariate logistic regression analysis with 95% confidence intervals.
	1.2	N		
	1.3	NA		
	1.4	Y		
	1.5	Y		
	1.6	N		
	1.7	NA		
	1.8	NA		
**Bias in selection of participants into the study**	2.1	N	Low	Comment: low risk of bias in selection of participants into the historical controlled trial
	2.2	NA		
	2.3	NA		
	2.4	Y		
	2.5	NA		
**Bias in classification of interventions**	3.1	Y	Moderate	Comment: potential slight recall bias in the retrospective cohort study
	3.2	N		
	3.3	N		
**Bias due to deviations from intended interventions (effect of assignment to intervention)**	4.1	NI	No Information	Comment: no information about deviation from intended interventions
	4.2	NA		
**Bias due to missing data**	5.1	Y	Low	Comment: no withdrawal
	5.2	N		
	5.3	N		
	5.4	NA		
	5.5	NA		
**Bias in measurement of outcomes**	6.1	Y	Serious	Comment: no information about blinding of outcome assessment
	6.2	NI		
	6.3	Y		
	6.4	N		
**Bias in selection of the reported result**	7.1	PN	Moderate	Comment: no preregistered protocol available, nor indication of selection of reported result
	7.2	PN		
	7.3	PN		
**Overall bias**	-	-	Serious	

Abbreviations: Y = yes, PY = probably yes, N = no, PN = probably no, NI = no information, NA = not applicable.

References

Hasegawa T, Ueda N, Yamada SI, Kato S, Iwata E, Hayashida S, Kojima Y, Shinohara M, Tojo I, Nakahara H, Yamaguchi T, Kirita T, Kurita H, Shibuya Y, Soutome S, Akashi M; Japanese Study Group of Co-operative Dentistry with Medicine (JCDM). Denosumab-related osteonecrosis of the jaw after tooth extraction and the effects of a short drug holiday in cancer patients: a multicenter retrospective study. Osteoporosis International 2021;32(11):2323-2333. [DOI: 10.1007/s00198-021-05995-3; EMBASE: 2011519833; PubMed: 33997909]

Kang 2020

**Methods**	**Study design**	Retrospective cohort study
	**Study period**	March 2008 to December 2017
	**Location**	Goyang, Korea
	**Number of centers**	1
	**Setting**	Department of Oral and Maxillofacial Surgery, National Health Insurance Service Ilsan Hospital
	**Funding source**	The Clinical Research Fund of the National Health Insurance Service Hospital (NHIMC2018CR016)
**Participants**	**Inclusion criteria**	Quote: "Only cases in which BP was formally prescribed by our hospital and we could access complete prescription records were included. All the patients included in the study received BP medication prior to tooth extraction."
	**Exclusion criteria**	Quote: (1) "Records that lacked information concerning the medication regimen and its duration, even if they were reported by the patient, were excluded from the study." (2) "None of the patients had undergone radiation therapy." (3) "Patients whose preoperative symptoms indicated the presence of MRONJ as defined by the American Association of Oral and Maxillofacial Surgeons guidelines were excluded." (4) "The study also excluded patients with an unclear diagnosis of MRONJ before tooth extraction who exhibited osteonecrosis during the procedure."
	**Number allocated**	**465 patients (1323 extractions):** 45 males/420 females, mean age 63.7 ± 10.5 for males and 69.3 ± 8.8 for females<br>**Systemic conditions:** osteoporosis (458 patients), cancer (6 patients) and unknown (1 patient)<br>**Drugs:** alendronate (439 patients), and ibandronate (56 patients) (There is some overlapping.)
	**Number withdrawn**	0
	**Number evaluated**	465 patients (1323 extractions)
**Interventions**	**Comparison**	**Drug holiday versus drug continuation**<br>**Group 1: 286 patients (786 extractions)**<br>Drug holiday for an average of 39.0 ± 35.5 months before extraction<br>**Group 2: 179 patients (537 extractions)**<br>Drug continuation
	**Other**	Quote: "Tooth extraction was performed under local anesthesia. Procedures consisted of the simple removal of the maxillary or mandibular teeth, surgical extractions requiring curettage, and surgical extractions requiring bone removal and root separation. After the extraction procedures, filling material (i.e., bone graft material such as collagen or fibrin) was not applied to the extraction socket. All the patients received oral prophylactic antibiotics 1 hour before the procedure. Patients exhibiting postoperative symptoms indicative of MRONJ were assumed to have developed the condition after the extractions."
	**Operators**	Not reported
**Outcomes**	**Outcomes**	Incidence of MRONJ (follow-up duration not reported)
**Notes**	**Sample size calculation**	Not reported
	**Baseline comparability**	"Table 1. Characteristics and incidence of post-extraction MRONJ according to BP discontinuation (n = 465 patients)" in the manuscript

Risk of Bias (ROBINS-I)

Domain	Signalling questions	Response	Reviewers' judgement	Description
**Bias due to confounding**	1.1	Y	Serious	Comment: no appropriate analysis.
	1.2	N		
	1.3	NA		
	1.4	N		
	1.5	NA		
	1.6	N		
	1.7	NA		
	1.8	NA		
**Bias in selection of participants into the study**	2.1	N	Low	Comment: low risk of bias in selection of participants into the retrospective cohort study
	2.2	NA		
	2.3	NA		
	2.4	Y		
	2.5	NA		
**Bias in classification of interventions**	3.1	Y	Moderate	Comment: potential slight recall bias in the retrospective cohort study
	3.2	N		
	3.3	N		
**Bias due to deviations from intended interventions (effect of assignment to intervention)**	4.1	NI	No Information	Comment: no information about deviation from intended interventions
	4.2	NA		
**Bias due to missing data**	5.1	Y	Low	Comment: no withdrawal
	5.2	N		
	5.3	N		
	5.4	NA		
	5.5	NA		
**Bias in measurement of outcomes**	6.1	Y	Serious	Comment: no information about blinding of outcome assessment
	6.2	NI		
	6.3	Y		
	6.4	N		
**Bias in selection of the reported result**	7.1	PN	Moderate	Comment: no preregistered protocol available, nor indication of selection of reported result
	7.2	PN		
	7.3	PN		
**Overall bias**	-	-	Serious	

Abbreviations: Y = yes, PY = probably yes, N = no, PN = probably no, NI = no information, NA = not applicable.

References

Kang SH, Park SJ, Kim MK. The effect of bisphosphonate discontinuation on the incidence of postoperative medication-related osteonecrosis of the jaw after tooth extraction. Journal of the Korean Association of Oral and Maxillofacial Surgeons 2020;46(1):78-83. [DOI: 10.5125/jkaoms.2020.46.1.78; EMBASE: 2005647602; PubMed: 32158685]

Mauceri 2020

**Methods**	**Study design**	Historical controlled trial with one prospective and one retrospective group
	**Study period**	August 2015 to April 2016 (prospective)
	**Location**	Palermo, Italy
	**Number of centers**	1
	**Setting**	Unit of Oral Medicine, University Hospital "Policlinico Paolo Giaccone"
	**Funding source**	None
**Participants**	**Inclusion criteria**	Quote: "Patients were eligible for the study if they: (I) were aged >18 years; (II) had treatment with BPs because of the underlying disease (OST or ONC); (III) required extraction due to infective-inflammatory dental disease; (IV) had a follow-up period of at least 24 months after dental extraction; (V) had an absence, at baseline, of clinic-radiological signs of medication-related ONJ, according to the Italian Societies of Maxillo-Facial Surgery and of Oral Pathology and Medicine (SICMF and SIPMO, respectively)."
	**Exclusion criteria**	Quote: "Patients were excluded from the study if they: (I) had clinical or radiological signs of ONJ in the surgical area; (II) had a previous history of irradiation to the head and neck area; (III) had neoplastic involvement of the jaws; (IV) were in poor general condition; (V) were pregnant or breast-feeding women."
	**Number allocated**	**20 patients (prospective)**<br>**Systemic conditions:** cancers (6 patients), and metabolic bone disease (14 patients)<br>**Drugs:** zoledronic acid (6 patients), alendronic acid (6 patients), clodronic acid (4 patients), ibandronic acid (2 patients) and risedronic acid (2 patients).<br>**905 patients (retrospective from electronic search of literature)**
	**Number withdrawn**	0
	**Number evaluated**	20 patients (63 extractions, prospective) and 905 patients (retrospective)
**Interventions**	**Comparison 1**	**APC (Autologous platelet concentrates) versus control**<br>**Group 1 (prospective): 20 patients (63 extractions)**<br>(1) Chlorhexidine 0.2% mouthwash 30mL swished up to 60 s;<br>(2) Local anesthesia, achieved using 3%mepivacaine hydrochloride without adrenaline;<br>(3) Elevation of a full-thickness mucoperiosteal flap;<br>(4) Tooth luxation and avulsion, gently performed with elevators and forceps;<br>(5) If necessary, subsequent osteoplasty by means of an ultrasonic surgical device;<br>(6) Debridement of the post-extraction socket with miller surgical curette and irrigation of the sockets with rifamycin sodium;<br>(7) Application of autologous **PRP (platelet-rich plasma)**;<br>(8) Tension-free soft tissue closure.<br>**Group 2 (retrospective from electronic search of literature): 905 patients**<br>(1) A well described surgical protocol (with an average of at least 2 teeth extracted);<br>(2) No use of PRP.
	**Comparison 2**	**Drug holiday versus drug continuation**<br>**Group 1: 4 patients (all from the prospective group)**<br>Drug holiday (bisphosphonates paused/completed) for an average of 7 ± 6.5 months before extraction<br>**Group 2: 16 patients (all from the prospective group)**<br>Drug continuation (bisphosphonates ongoing)
	**Other**	Not applicable
	**Operators**	Not reported
**Outcomes**	**Outcomes**	Incidence of delayed healing at 1 month<br>Incidence of MRONJ at 3 months, 6 months, 12 months, 18 months and 24 months (over 12 months in the retrospective group)
**Notes**	**Sample size calculation**	Not reported
	**Baseline comparability**	Not reported

Risk of Bias (ROBINS-I)

Domain	Signalling questions	Response	Reviewers' judgement	Description
**Bias due to confounding**	1.1	Y	Critical	Comment: confounding inherently not controllable because the control group was retrospective from electronic search of literature
	1.2	N		
	1.3	NA		
	1.4	N		
	1.5	NA		
	1.6	NI		
	1.7	NA		
	1.8	NA		
**Bias in selection of participants into the study**	2.1	PY	Critical	Comment: historical controlled trial with the control group retrospective from electronic search of published literature
	2.2	PY		
	2.3	PY		
	2.4	Y		
	2.5	N		
**Bias in classification of interventions**	3.1	Y	Low	Comment: low risk of bias in classification of interventions
	3.2	Y		
	3.3	N		
**Bias due to deviations from intended interventions (effect of assignment to intervention)**	4.1	NI	No information	Comment: no information about deviation from intended interventions
	4.2	NA		
**Bias due to missing data**	5.1	Y	Low	Comment: no withdrawal
	5.2	N		
	5.3	N		
	5.4	NA		
	5.5	NA		
**Bias in measurement of outcomes**	6.1	Y	Serious	Comment: no blinding of outcome assessment
	6.2	Y		
	6.3	Y		
	6.4	N		
**Bias in selection of the reported result**	7.1	PN	Moderate	Comment: no preregistered protocol available, nor indication of selection of reported result
	7.2	PN		
	7.3	PN		
**Overall bias**	-	-	Critical	

Abbreviations: Y = yes, PY = probably yes, N = no, PN = probably no, NI = no information, NA = not applicable.

References

Mauceri R, Panzarella V, Pizzo G, Oteri G, Cervino G, Mazzola G, Di Fede O, Campisi G. Platelet-Rich Plasma (PRP) in Dental Extraction of Patients at Risk of Bisphosphonate-Related Osteonecrosis of the Jaws: A Two-Year Longitudinal Study. Applied Sciences 2020;10(13):4487. [DOI: 10.3390/app10134487]

Montefusco 2008

**Methods**	**Study design**	Multicentre retrospective cohort study, cluster controlled
	**Study period**	Till September 2006
	**Location**	Italy
	**Number of centers**	2
	**Setting**	The Istituto Nazionale Tumori in Milan, and the Ospedale San Giovanni Battista in Turin
	**Funding source**	Not reported
**Participants**	**Inclusion criteria**	Patients with multiple myeloma treated with bisphosphonates
	**Exclusion criteria**	Not reported
	**Number allocated**	**24 patients with tooth extractions** (from 178 patients with or without dental procedures: 93 males/85 females, median age 61 ranged from 25 to 97)<br>**Systemic conditions:** multiple myeloma (all 24 patients)<br>**Drugs:** pamidronate and zoledronate (There is some overlapping.)
	**Number withdrawn**	0
	**Number evaluated**	24 patients
**Interventions**	**Comparison**	**Antibiotic prophylaxis versus control**<br>**Group 1: 14 patients**<br>The patients who had systematically received antibiotic prophylaxis before any dental procedure during bisphosphonate treatment.<br>Quote: "Antibiotic prophylaxis consisted in amoxicillin-clavulanate 1 gr bid p.o. or, much less frequently, in case of intolerance or allergy, levofloxacin 500 mg/day p.o., both from 1 day before to 3 days after any dental procedure. This scheme was derived from the institutional approach for prevention of bacteremia and infective endocarditis in case of dental procedures. No additional precautions were planned."<br>**Group 2: 10 patients**<br>The patients who did not receive any prophylaxis.
	**Other**	Not applicable
	**Operators**	Not reported
**Outcomes**	**Outcomes**	Incidence of MRONJ (follow-up duration not reported)
**Notes**	**Sample size calculation**	Not reported
	**Baseline comparability**	"Table I. Overall patient characteristics" and "Table II. Overall patient characteristics according to the exposure variable categories" in the manuscript

Risk of Bias (ROBINS-I)

**Domain**	**Signalling questions**	**Response**	**Reviewers' judgement**	**Description**
**Bias due to confounding**	1.1	Y	Serious	Comment: multivariate conditional logistic regression analysis was performed, but only p values were reported.
	1.2	N		
	1.3	NA		
	1.4	Y		
	1.5	N		
	1.6	N		
	1.7	NA		
	1.8	NA		
**Bias in selection of participants into the study**	2.1	N	Low	Comment: low risk of bias in selection of participants into the retrospective cohort study
	2.2	NA		
	2.3	NA		
	2.4	Y		
	2.5	NA		
**Bias in classification of interventions**	3.1	Y	Moderate	Comment: potential slight recall bias in the retrospective cohort study
	3.2	N		
	3.3	N		
**Bias due to deviations from intended interventions (effect of assignment to intervention)**	4.1	NI	No Information	Comment: no information about deviation from intended interventions
	4.2	NA		
**Bias due to missing data**	5.1	Y	Low	Comment: no withdrawal
	5.2	N		
	5.3	N		
	5.4	NA		
	5.5	NA		
**Bias in measurement of outcomes**	6.1	Y	Serious	Comment: no information about blinding of outcome assessment
	6.2	NI		
	6.3	Y		
	6.4	N		
**Bias in selection of the reported result**	7.1	PN	Moderate	Comment: no preregistered protocol available, nor indication of selection of reported result
	7.2	PN		
	7.3	PN		
**Overall bias**	-	-	Serious	

Abbreviations: Y = yes, PY = probably yes, N = no, PN = probably no, NI = no information, NA = not applicable.

References

Montefusco V, Gay F, Spina F, Miceli R, Maniezzo M, Teresa Ambrosini M, Farina L, Piva S, Palumbo A, Boccadoro M, Corradini P. Antibiotic prophylaxis before dental procedures may reduce the incidence of osteonecrosis of the jaw in patients with multiple myeloma treated with bisphosphonates. Leukemia and Lymphoma 2008;49(11):2156-2162. [DOI: 10.1080/10428190802483778; EMBASE: 352754765; PubMed: 19021059]

Mozzati 2012

**Methods**	**Study design**	Randomized controlled trial with two parallel groups
	**Study period**	January 2005 to December 2009
	**Location**	Turin, Italy
	**Number of centers**	1
	**Setting**	Oral Surgery Department, the Dental School of the University of Torino
	**Funding source**	Not reported
**Participants**	**Inclusion criteria**	(1) Current IV bisphosphonate therapy and (2) the necessity for removal of strongly compromised dental elements.
	**Exclusion criteria**	(1) Any previous history of irradiation to the maxillofacial area and (2) dental extractions before the study period
	**Number allocated**	**176 patients:** 75 males/101 females, age ranged from 44 to 83<br>**Systemic conditions:** prostatic carcinoma (60 patients), breast carcinoma (51 patients), multiple myeloma (57 patients), lung carcinoma (5 patients), and ovarian carcinoma (3 patients)<br>**Drugs:** zoledronic acid (all 176 patients)
	**Number withdrawn**	0
	**Number evaluated**	176 patients (542 extractions)
**Interventions**	**Comparison**	**APC (Autologous platelet concentrates) versus control**<br>**Group 1: 91 patients (275 extractions)**<br>Use of **PRGF (plasma rich in growth factors)** fraction inserted into the alveolus, and a membrane comprised of a plasma fraction poor in growth factors placed between the bone tissue and the mucosal flap<br>**Group 2: 85 patients (267 extractions)**<br>No use of PRGF.
	**Other**	Quote: "A professional oral hygiene session was given to each patient one week before surgery. All patients were administered the antibiotics amoxicillin/clavulanate potassium, at a dosage of 1-g tablet every 8 h for a total of 6 days, starting from the evening before the surgical appointment or erythromycin, at a dosage of 600-mg tablets every 8 h for 6 days, when an allergy to penicillin was declared."<br>"An alveolar troncular nerve block was administered to both groups via local or regional anesthesia (3% mepivacaine with 1:100,000 epinephrine), depending on the dental site. To prevent interference with the healing process, no intraligamentous or intrapapillary infiltrations were made. Surgical extractions were carried out via intrasulcular incisions and detachment of full thickness flaps to allow wound healing via primary intention to leave the post-extraction alveolus in contact with the oral cavity bacteria. To ensure nontraumatic avulsion, the dental extraction was followed by delicate curettage and osteoplastic procedures on the more fragile bone septum and cortical bone areas."<br>"Suturing was done in all cases with resorbable material (VycrilÒ 4/0, Ethicon, Inc., Somerville, New Jersey, US) using a simple detached technique ensuring a hermetic closure at the wound margins to enable healing via primary intention. Written oral hygiene and postoperative instructions were then given to all of the patients."
	**Operators**	Not reported
**Outcomes**	**Outcomes**	Incidence of delayed healing at 3 days, 7 days, 14 days, 21 days and 30 days (described in Methods but not reported in Results)<br>Incidence of MRONJ at 60 days, 90 days, 120 days, 12 months, 18 months, and 24-60 months
**Notes**	**Sample size calculation**	Not reported
	**Baseline comparability**	Principal patient characteristics was shown in Table 2 of this study, which was comparable.

Risk of Bias (RoB 2)

**Domain**	**Signalling questions**	**Response**	**Reviewers' judgement**	**Description**
**Bias arising from the randomization process**	1.1	Y	Some concerns	Quote: "The randomized group distribution was set up specifically to obtain groups that were homogenous for gender, age, smoking habits, systemic pathology based on the computerized clinical file we used in the first visit."
	1.2	NI		
	1.3	N		
**Bias due to deviations from the intended interventions (effect of assignment to intervention)**	2.1	NI	Some concerns	Comment: no information about deviation from intended interventions
	2.2	Y		
	2.3	NI		
	2.4	NA		
	2.5	NA		
	2.6	Y		
	2.7	NA		
**Bias due to missing data**	3.1	Y	Low	Comment: no withdrawal
	3.2	NA		
	3.3	NA		
	3.4	NA		
**Bias in measurement of outcomes**	4.1	N	High	Comment: no information about blinding of outcome assessment
	4.2	N		
	4.3	NI		
	4.4	Y		
	4.5	Y		
**Bias in selection of the reported result**	5.1	NI	Some concerns	Comment: no preregistered protocol available, nor indication of selection of reported result
	5.2	PN		
	5.3	PN		
**Overall bias**	-	-	High	

Abbreviations: Y = yes, PY = probably yes, N = no, PN = probably no, NI = no information, NA = not applicable.

References

[1] Mozzati M, Arata V, Gallesio G. Tooth extraction in patients on zoledronic acid therapy. Oral Oncology 2012;48(9):817-821. [CENTRAL: CN-00832646; DOI: 10.1016/j.oraloncology.2012.03.009; EMBASE: 51948154; PubMed: 22483860]

[2] Mozzati M, Arata V, Gallesio G, Carossa S. A dental extraction protocol with plasma rich in growth factors (PRGF) in patients on intravenous bisphosphonate therapy: a case-control study. Joint Bone Spine 2011;78(6):648-649. [CENTRAL: CN-00868286; DOI: 10.1016/j.jbspin.2011.04.017; EMBASE: 560071045; PubMed: 21703903]

Mozzati 2013

**Methods**	**Study design**	Randomized controlled trial with two parallel groups
	**Study period**	January 2005 to April 2011
	**Location**	Turin, Italy
	**Number of centers**	1
	**Setting**	Oral Surgery Department, the Dental School of the University of Torino
	**Funding source**	Not reported
**Participants**	**Inclusion criteria**	(1) Current oral bisphosphonate therapy, (2) treatment with oral bisphosphonates for more than 24 months, and (3) the necessity for the removal of compromised dental elements
	**Exclusion criteria**	(1) Any previous history of irradiation to the maxillofacial area and (2) dental extractions before the study period
	**Number allocated**	**700 patients:** 23 males/677 females, age ranged from 52 to 79<br>**Systemic conditions:** osteoporosis, rheumatoid arthritis, and Paget's disease<br>**Drugs:** alendronate (all 700 patients)
	**Number withdrawn**	0
	**Number evaluated**	700 patients (1480 extractions)
**Interventions**	**Comparison**	**Primary healing with mucoperiosteal flap versus secondary healing with no flap**<br>**Group 1: 334 patients (620 extractions)**<br>Healing by primary intention with full-thickness flap<br>Quote: "In the first group (Protocol A), the surgical extractions were carried out via intrasulcular incisions and detachment of full-thickness flaps to allow wound healing via primary intention and to leave the post-extraction alveolus in contact with the oral cavity bacteria. To ensure nontraumatic avulsion, the dental extraction was followed by delicate curettage and osteoplastic procedures on the more fragile bone septum and cortical bone areas."<br>**Group 2: 366 patients (860 extractions)**<br>Healing by secondary intention without flap<br>Quote: "In the second group (Protocol B), the extractions were carried out without detachment of full-thickness flaps; sockets were filled with absorbable gelatine sponge haemostatic to allow wound healing via secondary intention."
	**Other**	Quote: "A professional oral hygiene session was provided for each patient 1 week before surgery. All patients were administered with antibiotics amoxicillin and clavulanic acid at a dosage of one tablet every 12 h for a total of 6 days, starting from the evening before the surgical appointment. When an allergy to penicillin was declared, the patients were administered with erythromycin, at a dosage of one tablet every 8 h for a total of 6 days."<br>"An alveolar troncular nerve block was administered to both groups via local or regional anesthesia (3 % mepivacaine with 1:100,000 epinephrine), depending on the dental site. To prevent interference with the healing process, no intraligamentous or intrapapillary infiltrations were made."<br>"Suturing was done in all cases with a resorbable material (Vycril® 4/0, Ethicon, Inc., Somerville, New Jersey, US). Written oral hygiene instructions regarding the correct maintenance of the surgical extraction sites were subsequently given to all of the patients."
	**Operators**	Three surgeons with the same surgical experience
**Outcomes**	**Outcomes**	Incidence of delayed healing at 3 days, 7 days, 14 days, 21 days and 30 days<br>Incidence of MRONJ at 60 days, 90 days, and 12-72 months
**Notes**	**Sample size calculation**	Not reported
	**Baseline comparability**	Principal patient characteristics was shown in Table 2 of this study, which was comparable.

Risk of Bias (RoB 2)

Domain	Signalling questions	Response	Reviewers' judgement	Description
**Bias arising from the randomization process**	1.1	Y	Some concerns	Quote: "The randomized group distribution was set up specifically to obtain groups that were homogenous for gender, age, smoking habits, and health status based on the computerized clinical file we used in the first visit."
	1.2	NI		
	1.3	N		
**Bias due to deviations from the intended interventions (effect of assignment to intervention)**	2.1	NI	Some concerns	Comment: no information about deviation from intended interventions
	2.2	Y		
	2.3	NI		
	2.4	NA		
	2.5	NA		
	2.6	Y		
	2.7	NA		
**Bias due to missing data**	3.1	Y	Low	Comment: no withdrawal
	3.2	NA		
	3.3	NA		
	3.4	NA		
**Bias in measurement of outcomes**	4.1	N	High	Comment: no information about blinding of outcome assessment
	4.2	N		
	4.3	NI		
	4.4	Y		
	4.5	Y		
**Bias in selection of the reported result**	5.1	NI	Some concerns	Comment: no preregistered protocol available, nor indication of selection of reported result
	5.2	PN		
	5.3	PN		
**Overall bias**	-	-	High	

Abbreviations: Y = yes, PY = probably yes, N = no, PN = probably no, NI = no information, NA = not applicable.

References

[1] Mozzati M, Arata V, Gallesio G. Tooth extraction in osteoporotic patients taking oral bisphosphonates. Osteoporosis International 2013;24(5):1707-1712. [CENTRAL: CN-00873847; DOI: 10.1007/s00198-012-2239-8; EMBASE: 52379364; PubMed: 23288026]

[2] Mozzati M, Arata V, Gallesio G, Carossa S. Tooth extraction and oral bisphosphonates: comparison of different surgical protocols. Joint Bone Spine 2011;78(6):647-648. [CENTRAL: CN-00972451; DOI: 10.1016/j.jbspin.2011.04.018; EMBASE: 51488851; PubMed: 21703902]

Ottesen 2022

**Methods**	**Study design**	Single-blind randomized controlled trial with two parallel groups
	**Study period**	July 2018 to November 2019
	**Location**	Copenhagen, Denmark
	**Number of centers**	1
	**Setting**	Department of Oral & Maxillofacial Surgery, Copenhagen University Hospital
	**Funding source**	The Research Foundation of the Danish Dental Association
**Participants**	**Inclusion criteria**	(1) Malignant disease (breast cancer, prostate cancer, multiple myeloma, or bone metastases); (2) high-dose antiresorptives for at least 1 month; (3) need of tooth extraction; (4) ECOG (Eastern Cooperative Oncology Group) score ≤ 2; (5) >18 years old; and (6) informed consent.
	**Exclusion criteria**	(1) Radiation therapy to the jaws; (2) existing or previous MRONJ; (3) unable to cooperate; or (4) withdrawal of informed consent before surgical tooth extraction.
	**Number allocated**	**23 patients:** 11 males/12 females, age ranged from 56 to 78<br>**Systemic conditions:** breast cancer (11 patients), prostate cancer (4 patients), and multiple myeloma (8 patients)<br>**Drugs:** bisphosphonates (Zometa or Pamifos) (10 patients) and denosumab (13 patients)
	**Number withdrawn**	3 patients (due to death)
	**Number evaluated**	23 patients (31 extractions) (intention-to-treat analysis)
**Interventions**	**Comparison**	**Drug holiday versus drug continuation**<br>**Group 1: 13 patients**<br>Drug holiday for 4 months (1 month before extraction and 3 months after extraction)<br>**Group 2: 10 patients**<br>Drug continuation
	**Other**	Quote: "The surgical tooth extraction was according to the Danish Standard Operation Procedure. In brief, antibiotic prophylaxis was initiated 1 hour preoperatively with amoxicillin 1000 mg and clavulanic acid 250 mg tablets or clindamycin 600 mg in case of penicillin allergy. The antibiotic prophylaxis was continued for 10 days postoperatively (500 mg amoxicillin and 125 mg clavulanic tablets three times daily or clindamycin tablets 300 mg three times daily if penicillin allergy). The oral cavity, including the surgical site, was rinsed with chlorhexidine 0.12% preoperatively. All patients had surgery under local anesthesia (Lidocaine 10 mg/mL, epinephrine 5 mg/mL). Surgical extractions were carried out using an intrasulcular incision with 2 facial releasing incisions. After low-trauma tooth extraction, sharp bone edges were smoothened, and tension-free mucosal wound closure was obtained after flap mobilization. The extraction site was cleaned with sterile saline to remove debris. Single sutures were used, either Ethicon Vicryl 4-0 suture or Prolene 5-0 suture. All surgical tooth extractions were performed by the same maxillofacial surgeon (M.S.). Oral and written postoperative instructions were given to the patient by the clinical investigator (C.O.). The patients were prescribed chlorhexidine 0.12% mouth rinse twice daily until suture removal. Sutures were removed approximately 10 days after tooth extraction."
	**Operators**	One surgeon, blinded to the allocation
**Outcomes**	**Outcomes**	Incidence of MRONJ at 3 months and 6 months<br>Mortality (MRONJ-related and all-cause) at 3 months and 6 months<br>Quality of life (EQ-5D-5L and EQ-VAS) (ineligible for meta-analysis)<br>Incidence of complications (skeletal pain and/or fracture) (ineligible for meta-analysis)
**Notes**	**Sample size calculation**	A feasibility study without a large enough sample size
	**Baseline comparability**	Baseline patient characteristics was shown in Table II and allocation shown in Table III of this study, which was likely to be comparable.

Risk of Bias (RoB 2)

Domain	Signalling questions	Response	Reviewers' judgement	Description
**Bias arising from the randomization process**	1.1	Y	Low	Quote: "Block randomization was performed to equal the sample size for the 2 treatment modalities. Concealed envelopes were used as a randomization procedure at the inclusion day."
	1.2	Y		
	1.3	NI		
**Bias due to deviations from the intended interventions (effect of assignment to intervention)**	2.1	Y	Some concerns	Quote: "However, the patients themselves, as well as the patient's oncologist doctor and nurse, who were in charge of drug delivery, were not blinded."<br>Comment: no information about deviation from intended interventions
	2.2	Y		
	2.3	NI		
	2.4	NA		
	2.5	NA		
	2.6	Y		
	2.7	NA		
**Bias due to missing data**	3.1	N	Low	Comment: Three withdrawals due to death were included into intention-to-treat analysis, and results would be little changed under sensitivity analysis.
	3.2	Y		
	3.3	NA		
	3.4	NA		
**Bias in measurement of outcomes**	4.1	N	Low	Quote: "The clinical investigator (C.O.), the surgeon (M.S.), and the outcome assessor (C.O.) were blinded to the group allocation of the patients."
	4.2	N		
	4.3	N		
	4.4	NA		
	4.5	NA		
**Bias in selection of the reported result**	5.1	Y	Low	Comment: A protocol showed no selection of reported result.
	5.2	N		
	5.3	N		
**Overall bias**	-	-	Some concerns	

Abbreviations: Y = yes, PY = probably yes, N = no, PN = probably no, NI = no information, NA = not applicable.

References

[1] Ottesen C, Schiodt M, Jensen SS, Kofod T, Gotfredsen K. Tooth extractions in patients with cancer receiving high-dose antiresorptive medication: a randomized clinical feasibility trial of drug holiday versus drug continuation. Oral Surgery, Oral Medicine, Oral Pathology, Oral Radiology 2022;133(2):165-173. [CENTRAL: CN-02300591; DOI: 10.1016/j.oooo.2021.06.003; EMBASE: 635641234; PubMed: 34275774]

[2] NCT04540601. Antiresorptive Drug Continuation Compared With Drug Holiday in Cancer Patients Needing Tooth Extraction (DrugHoliday). https://clinicaltrials.gov/ct2/show/NCT04540601 (First Submitted August 24, 2020). [CENTRAL: CN-02163445; ICTRP: https://trialsearch.who.int/?TrialID=NCT04540601]

Poxleitner 2020

**Methods**	**Study design**	Randomized controlled trial with two parallel groups
	**Study period**	2017 to 2019
	**Location**	Freiburg, Germany
	**Number of centers**	1
	**Setting**	Department of Oral and Maxillofacial Surgery, University Medical Center Freiburg
	**Funding source**	The University of Freiburg Faculty of Medicine Research Committee in the program "Clinical Studies"
**Participants**	**Inclusion criteria**	(1) Diagnosis of osteoporosis, (2) current or previous antiresorptive therapy (bisphosphonates or denosumab) for osteoporosis, and (3) indication for extraction of one or more teeth with a hopeless prognosis
	**Exclusion criteria**	Presented with a history of irradiation to the maxillofacial region or neoplastic involvement/disease of the maxillofacial region
	**Number allocated**	**77 patients:** 1 males/76 females, median age 78 ranged from 44 to 88<br>**Systemic conditions:** osteoporosis (all 77 patients)<br>**Drugs:** alendronic acid (28 patients), ibandronic acid (9 patients), pamidronic acid (1 patient), risedronic acid (8 patients), zoledronic acid (7 patients), and denosumab (24 patients)
	**Number withdrawn**	0
	**Number evaluated**	77 patients
**Interventions**	**Comparison**	**APC (Autologous platelet concentrates) versus control**<br>**Group 1: 38 patients**<br>Insertion of a **PRF (platelet-rich fibrin)** clot into the extraction socket without subsequent primary closure.<br>Quote: "After gentle curetting, the extraction socket was filled with the PRF plug and a cross suture (Vicryl 4-0) was used to stabilize the plug in the extraction socket."<br>**Group 2: 39 patients**<br>Primary closure of the extraction socket with a mucoperiosteal flap.<br>Quote: "In this group, a mucoperiosteal flap was reflected with care to prevent traumatization of the soft tissue. An incision of the buccal periosteum was performed to allow a tension-free closure of the extraction socket. With a first set of stiches (Vicryl 3-0), the flap was re-approximated, aiming at complete mucosal coverage of the bone. This was followed by a layer of running sutures (Vicryl 4-0)."
	**Other**	Quote: "All patients received perioperative intravenous antibiotic therapy (penicillin 10, 000, 000 IU once daily or clindamycin 600 mg three times daily in case of penicillin allergy), initiated 1 day before surgery and continued until 1 day after surgery."<br>"Prior to the extraction, all patients rinsed their mouth with chlorhexidine solution. All interventions were performed by one experienced surgeon. After local anesthesia with 4% articaine with 1:200,000 epinephrine, a sulcular incision was performed. Teeth were then removed in the most atraumatic manner possible with subsequent gentle curettage of the extraction socket and rounding of sharp bony edges."<br>"Postoperatively, patients were instructed to consume a soft diet, to apply daily mouth rinses with chlorhexidine solution, and to refrain from wearing dentures until complete mucosal healing was achieved."
	**Operators**	One experienced surgeon
**Outcomes**	**Outcomes**	Incidence of MRONJ at 90 days (3 months)<br>Incidence of complications (ineligible for meta-analysis)
**Notes**	**Sample size calculation**	Not reported
	**Baseline comparability**	Quote: "There were no statistically significant differences between the two groups ($p > 0.05$) considering age and gender, duration and type of antiresorptive therapy, and the teeth to be extracted (number, location, type) (Tables 1a and b)."

Risk of Bias (RoB 2)

**Domain**	**Signalling questions**	**Response**	**Reviewers' judgement**	**Description**
**Bias arising from the randomization process**	1.1	Y	Some concerns	Quote: “Assignment to one of the two groups was performed via block randomization with randomly selected block sizes.”
	1.2	NI		
	1.3	N		
**Bias due to deviations from the intended interventions (effect of assignment to intervention)**	2.1	NI	Some concerns	Comment: no information about deviation from intended interventions
	2.2	Y		
	2.3	NI		
	2.4	NA		
	2.5	NA		
	2.6	Y		
	2.7	NA		
**Bias due to missing data**	3.1	Y	Low	Comment: no withdrawal
	3.2	NA		
	3.3	NA		
	3.4	NA		
**Bias in measurement of outcomes**	4.1	N	High	Comment: no information about blinding of outcome assessment
	4.2	N		
	4.3	NI		
	4.4	Y		
	4.5	Y		
**Bias in selection of the reported result**	5.1	NI	Some concerns	Comment: no preregistered protocol available, nor indication of selection of reported result
	5.2	PN		
	5.3	PN		
**Overall bias**	-	-	High	

Abbreviations: Y = yes, PY = probably yes, N = no, PN = probably no, NI = no information, NA = not applicable.

References

Poxleitner P, Steybe D, Kroneberg P, Ermer MA, Yalcin-Ülker GM, Schmelzeisen R, Voss PJ. Tooth extractions in patients under antiresorptive therapy for osteoporosis: Primary closure of the extraction socket with a mucoperiosteal flap versus application of platelet-rich fibrin for the prevention of antiresorptive agent-related osteonecrosis of the jaw. Journal of Cranio-Maxillo-Facial Surgery 2020;48(4):444-451. [DOI: 10.1016/j.jcms.2020.02.006; EMBASE: 2005098500; PubMed: 32122726]

Ristow 2021

**Methods**	**Study design**	Double-blind randomized controlled trial with two parallel groups
	**Study period**	April 2016 to April 2018
	**Location**	Heidelberg, Germany
	**Number of centers**	1
	**Setting**	Department of Cranio-, Oral-, and Maxillofacial Surgery, University of Heidelberg
	**Funding source**	DFG-German Research foundation (Grant number RI2813/2-1, 2016)
**Participants**	**Inclusion criteria**	Quote: "Inclusion criteria comprised clinical and radiological indications for tooth extractions because of either (a) symptomatic teeth/roots with acute dental infections, that is, dental pain and/or infection signs (redness, swelling, purulent discharge from the tooth socket) and/or painful tooth mobility, or (b) asymptomatic non-restorable teeth/roots (residual roots, fractured teeth, teeth destroyed by deep caries, non-painful tooth mobility) and an ongoing or previous history of antiresorptive treatment, stratified either as cancer patients (malignant disease with bone metastasis or multiple myeloma, with monthly high-dose antiresorptive therapy delivered intravenously [bisphosphonate] or subcutaneously [denosumab]) or as osteoporosis patients (weekly low-dose antiresorptive therapy administered orally [bisphosphonate] or half-yearly subcutaneously [denosumab])."
	**Exclusion criteria**	Quote: "Exclusion criteria were (a) patients with malignant disease without metastasis and adjuvant antiresorptive treatment, (b) fistula to the teeth, (c) exposed bone or existing diagnosis of ONJ at the extraction site, (d) impacted wisdom teeth, (e) history of head and neck radiation, (f) known malignant or metastatic bone disease of the maxillofacial region, and (g) patients younger than 18."
	**Number allocated**	**160 patients:** 43 males/117 females, mean age 68.1 ± 9.8<br>**Systemic conditions:** osteoporosis (73 patients), breast cancer (46 patients), prostate cancer (12 patients), multiple myeloma (23 patients), and other cancers (6 patients)<br>**Drugs:** bisphosphonates (130 patients) and denosumab (46 patients) (There is some overlapping.)
	**Number withdrawn**	28 patients (6 due to death, and 22 due to other reasons)
	**Number evaluated**	160 patients (475 extractions) (intention-to-treat analysis)
**Interventions**	**Comparison**	**Primary healing with mucoperiosteal flap versus primary healing with mucosal flap**<br>**Group 1: 82 patients**<br>Healing by primary intention with a sub-periosteally prepared (SPP) muco-periosteal flap<br>**Group 2: 78 patients**<br>Healing by primary intention with an epi-periosteally prepared (EPP) mucosa flap
	**Other**	Quote: "All surgical tooth extractions were performed following the standardized protocol and preventive measures for patients undergoing/after antiresorptive treatment (Schiegnitz et al., 2018): (a) adjunctive antibiotic therapy, (b) in an atraumatic manner, succeeded by (c) alveoplasty, and (d) thorough primary wound closure."<br>"In detail, all patients were pretreated with oral antibiotics (sultamicillin PD 375 mg 1–0–1) starting on the week before the surgical intervention and lasting for 1 week after surgery. In patients who reported a history of hypersensitivity to penicillin or a penicillin allergy, clindamycin (600 mg) was used instead. Additionally, all patients used an antimicrobial mouth wash (0.2% chlorhexidine solution; GlaxoSmithKline Consumer Healthcare GmbH & Co. KG) three times a day, starting 2 days before surgery and lasting for at least 5 days after surgery."
	**Operators**	Five experienced surgeon
**Outcomes**	**Outcomes**	Incidence of MRONJ at 2 months<br>Mortality (all-cause) at 1 month, 2 months, 4 months, and 6 months<br>"Secondary endpoints" (ineligible for meta-analysis)

**Notes**	**Sample size calculation**	No formal sample size calculation
	**Baseline comparability**	Quote: "Baseline characteristics were balanced across groups (Table 1) (p < .05)."

Risk of Bias (RoB 2)

**Domain**	**Signalling questions**	**Response**	**Reviewers' judgement**	**Description**
**Bias arising from the randomization process**	1.1	Y	Low	Quote: "An independent statistical consultant was employed to computer generate the randomization sequence via a centralized web-based tool (www.randomizer.at) prior to the start of the study. This generated a pseudorandom code with permuted blocks of randomly variable size. The sequence was known only to the programmer until the database lock. No one directly involved in the project had access to the allocation codes. Eligibility and enrollment were performed by an independent physician (not involved in the surgical procedure) during the specialized consultation hours. Subsequently, the allocation of the patients to the treatment groups was performed by the surgeons by means of sealed envelopes immediately before surgery."
	1.2	Y		
	1.3	N		
**Bias due to deviations from the intended interventions (effect of assignment to intervention)**	2.1	N	Some concerns	Quote: "To minimize detection bias, patients were not informed about their allocation."<br><br>Comment: However, 8 patients switched from their intended interventions, but no reasons were reported.
	2.2	Y		
	2.3	PY		
	2.4	N		
	2.5	NA		
	2.6	Y		
	2.7	NA		
**Bias due to missing data**	3.1	N	High	Comment: 28 withdrawals (17.5%) from 160 participants
	3.2	N		
	3.3	Y		
	3.4	PY		
**Bias in measurement of outcomes**	4.1	N	Low	Quote: "The physicians and research nurses who carried out the postoperative follow-ups and assessed the outcomes and the statistician were all blinded to treatment allocation during the entire study."
	4.2	N		
	4.3	N		
	4.4	NA		
	4.5	NA		
**Bias in selection of the reported result**	5.1	Y	Low	Comment: A protocol showed no selection of reported result.
	5.2	N		
	5.3	N		
**Overall bias**	-	-	High	

Abbreviations: Y = yes, PY = probably yes, N = no, PN = probably no, NI = no information, NA = not applicable.

References

[1] Ristow O, Rückschloß T, Moratin J, Müller M, Kühle R, Dominik H, Pilz M, Shavlokhova V, Otto S, Hoffmann J, Freudlsperger C. Wound closure and alveoplasty after preventive tooth extractions in patients with antiresorptive intake-A randomized pilot trial. Oral Diseases 2021;27(3):532-546. [CENTRAL: CN-02161387; DOI: 10.1111/odi.13556; EMBASE: 2005808565; PubMed: 32875698]

[2] DRKS00010106. Comparison of two different mucosal closure techniques after tooth extraction in high risk patients with antiresortive medication intake - a prospective, randomized, blinded feasebility study. https://www.drks.de/DRKS00010106 (First Submitted March 30, 2016). [CENTRAL: CN-01852890; ICTRP: https://trialsearch.who.int/?TrialID=DRKS00010106]

Sanchis 2014

**Methods**	**Study design**	Prospective cohort study
	**Study period**	January 2009 to February 2011
	**Location**	Valencia, Spain
	**Number of centers**	1
	**Setting**	Department of Stomatology and Maxillofacial Surgery, Valencia University General Hospital
	**Funding source**	Not reported
**Participants**	**Inclusion criteria**	Quote: "These patients had been treated or were receiving treatment with intravenous zoledronic acid (Zometa, Novartis)."
	**Exclusion criteria**	Not reported
	**Number allocated**	**36 patients:** 16 males/20 females, mean age 63.81 ± 11.4<br>**Drugs:** zoledronic acid (all 36 patients)
	**Number withdrawn**	2 patients (1 due to death, and 1 due to other reasons)
	**Number evaluated**	34 patients (62 extractions)<br>**Systemic conditions:** multiple myeloma (18 patients), breast cancer (10 patients), prostate cancer (4 patients), bladder cancer (1 patient), and Crohn's disease (1 patient)
**Interventions**	**Comparison**	**Drug holiday versus drug continuation**<br>**Group 1: 23 patients**<br>Drug holiday (zoledronic acid suspended for an average of 5.6 months before extraction)<br>**Group 2: 11 patients**<br>Drug continuation (zoledronic acid ongoing)
	**Other**	Quote: "a preventive protocol consisting of the following: antibiotic prophylaxis (amoxicillin with clavulanic acid 875/125 mg every 8 hours) from 2 days before extraction to 15 days after tooth removal. In the case of allergy to penicillin, clindamycin was administered 300 mg every 8 hours. Tooth extraction was carried out causing as little trauma as possible, with maximum preservation of soft tissues, irrigating the surgical field several times with 0.12% chlorhexidine solution, smoothing the bone margins, and suturing with double-zero silk in most cases. The patients were instructed to perform rinses with 0.12% chlorhexidine solution three to four times a day at home."
	**Operators**	One surgeons
**Outcomes**	**Outcomes**	Incidence of MRONJ at 4 months
**Notes**	**Sample size calculation**	Not reported
	**Baseline comparability**	Not reported

Risk of Bias (ROBINS-I)

**Domain**	**Signalling questions**	**Response**	**Reviewers' judgement**	**Description**
**Bias due to confounding**	1.1	Y	Serious	Comment: baseline confounding factors not controlled
	1.2	N		
	1.3	NA		
	1.4	N		
	1.5	NA		
	1.6	N		
	1.7	NA		
	1.8	NA		
**Bias in selection of participants into the study**	2.1	N	Low	Comment: low risk of bias in selection of participants into the historical controlled trial
	2.2	NA		
	2.3	NA		
	2.4	Y		
	2.5	NA		
**Bias in classification of interventions**	3.1	Y	Low	Comment: low risk of bias in classification of interventions
	3.2	Y		
	3.3	N		
**Bias due to deviations from intended interventions (effect of assignment to intervention)**	4.1	NI	No information	Comment: no information about deviation from intended interventions
	4.2	NA		
**Bias due to missing data**	5.1	N	Moderate	Comment: 2 withdrawals (5.6%) from 36 participants
	5.2	N		
	5.3	N		
	5.4	NI		
	5.5	PN		
**Bias in measurement of outcomes**	6.1	Y	Serious	Comment: no information about blinding of outcome assessment
	6.2	NI		
	6.3	Y		
	6.4	N		
**Bias in selection of the reported result**	7.1	PN	Moderate	Comment: no preregistered protocol available, nor indication of selection of reported result
	7.2	PN		
	7.3	PN		
**Overall bias**	-	-	Serious	

Abbreviations: Y = yes, PY = probably yes, N = no, PN = probably no, NI = no information, NA = not applicable.

References

Sanchis JM, Bagán JV, Murillo J, Díaz JM, Asensio L. Risk of developing BRONJ among patients exposed to intravenous bisphosphonates following tooth extraction. Quintessence International 2014;45(9):769-777. [DOI: 10.3290/j.qi.a32243; EMBASE: 611356233; PubMed: 25019117]

Scoletta 2013

**Methods**	**Study design**	Historical controlled trial with two prospective groups
	**Study period**	July 2007 to June 2009 (first prospective, Group 1);<br>March 2010 to September 2011 (last prospective, Group 2);
	**Location**	Turin, Italy
	**Number of centers**	1
	**Setting**	Oral Surgery Unit, Department of Clinical Physiopathology, Lingotto Dental School
	**Funding source**	Not reported
**Participants**	**Inclusion criteria**	(1) Patients at least 18 years old who used intravenous BPs for at least 2 months, (2) the ability to complete the clinical trial, (3) no clinical signs of BRONJ during the first visit, and (4) a follow-up of at least 4 months.
	**Exclusion criteria**	(1) Tooth extraction in the 3 months before the study, (2) pregnant or breast-feeding women, and (3) confirmed or suspected hypersensitivity to any medication used.
	**Number allocated**	**127 patients:** 38 males/89 females<br>**Group 1:** 64 patients, mean age 64.81 ± 10.98<br>**Systemic conditions in Group 1:** osteoporosis (2 patients), rheumatoid arthritis (1 patients), breast cancer (32 patients), prostate cancer (4 patients), multiple myeloma (21 patients), lung cancer (1 patient), ovarian cancer (1 patient), rhinopharynx cancer (1 patient), and Paget's disease (1 patient)<br>**Drugs in Group 1:** pamidronate (7 patients) and zoledronic acid (62 patients) (There is some overlapping.)<br>**Group 2:** 63 patients, mean age 65.82 ± 8.82<br>**Systemic conditions in Group 2:** osteoporosis (6 patients), breast cancer (30 patients), prostate cancer (5 patients), multiple myeloma (20 patients), lymphoma (1 patient), and lung cancer (1 patient)<br>**Drugs in Group 2:** pamidronate (4 patients), ibandronate (5 patients), and zoledronic acid (54 patients)
	**Number withdrawn**	0
	**Number evaluated**	127 patients (218 extractions)
**Interventions**	**Comparison**	**Secondary healing with mucoperiosteal flap versus secondary healing with no flap**<br>**Group 1 (first prospective): 64 patients (220 extractions)**<br>Healing by secondary intention with a vestibular split-thickness flap;<br>The flap was sutured with interrupted sutures using Vicryl 4-0.<br>**Group 2 (last prospective): 63 patients (202 extractions)**<br>Healing by secondary intention without a vestibular split-thickness flap;<br>A Vicryl 4-0 cross-suturing technique was used for maintaining the stability of the PRGF.
	**Other**	Quote: "Patients underwent dental panoramic radiography. Two weeks before tooth extraction, each patient underwent an initial treatment consisting of root scaling and oral hygiene instruction. The evening before surgery, systemic antibiotic therapy with amoxicillin/clavulanate potassium (1-g tablets every 8 hr for 6 days) was commenced or, alternatively, erythromycin (600-mg tablets every 8 hr for 6 days) was used if there was an allergy to penicillin."<br>"Dental nerve anesthesia was achieved using 3% mepivacaine hydrochloride and epinephrine 1:100,000. Tooth luxation and avulsion were gently performed using appropriate hand instruments. An ultrasonic surgical apparatus was used for cleaning the postextraction alveolar sockets and for minimal osteoplasty of the alveolar ridge to avoid sharp surfaces that might delay postoperative healing. Extraction sockets were then filled with scaffold-like autologous PRGF and sealed with autologous fibrin (both formulations obtained from the patient)."<br>"Patients were given standard postoperative instructions and instructed not to brush the teeth in the treated area but to gently clean the wound using a gauze impregnated with 3% hydrogen peroxide 3 times daily for 2 weeks. A cold semiliquid diet for the first day was suggested. Normal oral hygiene procedures were re-established after 3 days."
	**Operators**	The same three experienced surgeons

**Outcomes**	**Outcomes**	Incidence of MRONJ at 4-12 months
**Notes**	**Sample size calculation**	Not reported
	**Baseline comparability**	Baseline patient characteristics was shown in Table 3 of this study, which was likely to be comparable.

Risk of Bias (ROBINS-I)

Domain	Signalling questions	Response	Reviewers' judgement	Description
**Bias due to confounding**	1.1	Y	Serious	Comment: baseline confounding factors not controlled
	1.2	N		
	1.3	NA		
	1.4	N		
	1.5	NA		
	1.6	N		
	1.7	NA		
	1.8	NA		
**Bias in selection of participants into the study**	2.1	Y	Serious	Comment: Only participants with follow-up of at least 4 months were included in the study.
	2.2	Y		
	2.3	Y		
	2.4	Y		
	2.5	N		
**Bias in classification of interventions**	3.1	Y	Low	Comment: low risk of bias in classification of interventions
	3.2	Y		
	3.3	N		
**Bias due to deviations from intended interventions (effect of assignment to intervention)**	4.1	NI	No information	Comment: no information about deviation from intended interventions
	4.2	NA		
**Bias due to missing data**	5.1	Y	Low	Comment: no withdrawal
	5.2	N		
	5.3	N		
	5.4	NA		
	5.5	NA		
**Bias in measurement of outcomes**	6.1	Y	Serious	Comment: no blinding of outcome assessment
	6.2	Y		
	6.3	Y		
	6.4	N		
**Bias in selection of the reported result**	7.1	PN	Moderate	Comment: no preregistered protocol available, nor indication of selection of reported result
	7.2	PN		
	7.3	PN		
**Overall bias**	-	-	Serious	

Abbreviations: Y = yes, PY = probably yes, N = no, PN = probably no, NI = no information, NA = not applicable.

References

[1] Scoletta M, Arata V, Arduino PG, Lerda E, Chiecchio A, Gallesio G, Scully C, Mozzati M. Tooth extractions in intravenous bisphosphonate-treated patients: a refined protocol. Journal of Oral and Maxillofacial Surgery 2013;71(6):994-999. [DOI: 10.1016/j.joms.2013.01.006; EMBASE: 52454477; PubMed: 23434159]

[2] Scoletta M, Arduino PG, Pol R, Arata V, Silvestri S, Chiecchio A, Mozzati M. Initial experience on the outcome of teeth extractions in intravenous bisphosphonate-treated patients: a cautionary report. Journal of Oral and Maxillofacial Surgery 2011;69(2):456-462. [DOI: 10.1016/j.joms.2010.07.026; EMBASE: 51177140; PubMed: 21129835]

Characteristics of Excluded Studies (46 studies with 57 reports)

Primary studies excluded due to focused clinical questions (10 studies with 12 reports)

Study ID	References	Reason for exclusion
**Furuya 2017**	**[1]** Furuya T, Maeda S, Momohara S, Taniguchi A, Yamanaka H. Dental treatments, tooth extractions, and osteonecrosis of the jaw in Japanese patients with rheumatoid arthritis: results from the IORRA cohort study. Journal of Bone and Mineral Metabolism 2017;35(3): 344-350. [DOI: 10.1007/s00774-016-0763-x; EMBASE: 611051833 ; PubMed: 27372662]<br>**[2]** Furuya T, Momohara S, Taniguchi A, Yamanaka H. Dental Treatments, Tooth Extraction, and Osteonecrosis at Jaw in Japanese Patients with Rheumatoid Arthritis: Results from the IORRA Cohort Study. Arthritis and Rheumatology 2015;67(S10):Abstract Number 384. [DOI: 10.1002/art.39448; EMBASE: 72094275]<br>**[3]** Furuya T, Momohara S, Taniguchi A, Yamanaka H. Dental history and complications in Japanese patients with rheumatoid arthritis: Results from the IORRA cohort study. Annals of the Rheumatic Diseases 2015;74(Suppl 2):1205. [DOI: 10.1136/annrheu mdis-2015-eular.1315; EMBASE: 72154319]	Prospective cohort study, aimed to evaluate the risk factors of MRONJ
**Guo 2021**	**[4]** Guo YX, Wang DC, Liu XJ, Wang EB, An JG, Peng X, Guo CB. Evaluation of the preliminary clinical effect of flap-raising combined with cortical-perforation technique in tooth extraction cases of patients with potential risk of medication-related osteonecrosis of the jaw. Chinese Journal of Stomatology 2021;56(5):452-457. [DOI: 10.3760/cma.j.cn112144-20210104-00003; EMBASE: 634895173; PubMed: 33904280]	Retrospective cohort study, comparing different durations of bisphosphonate administration
**Huang 2015**	**[5]** Huang YF, Chang CT, Muo CH, Tsai CH, Shen YF, Wu CZ. Impact of bisphosphonate-related osteonecrosis of the jaw on osteo-porotic patients after dental extraction: a population-based cohort study. PLoS One 2015;10(4):e0120756. [DOI: 10.1371/journal.pone. 0120756; EMBASE: 603863824; PubMed: 25880208]	Prospective cohort study, aimed to evaluate osteoporosis as a risk factor of MRONJ
**Lesclous 2020**	**[6]** Lesclous P, Cloitre A, Catros S, Devoize L, Louvet B, Châtel C, Foissac F, Roux C. Alendronate or Zoledronic acid do not impair wound healing after tooth extraction in postmenopausal women with osteoporosis. Bone 2020;137:115412. [DOI: 10.1016/j.bone.2020. 115412; EMBASE: 631768194; PubMed: 32404281]	Prospective cohort study, aimed to evaluate bisphosphonates as a risk factor of MRONJ
**Migliorati 2013**	**[7]** Migliorati CA, Saunders D, Conlon MS, Ingstad HK, Vaagen P, Palazzolo MJ, Herlofson BB. Assessing the association between bisphosphonate exposure and delayed mucosal healing after tooth extraction. Journal of the American Dental Association 2013;144(4): 406-414. [DOI: 10.14219/jada.archive.2013.0134; EMBASE: 36882 9769; PubMed: 23543695]	Prospective cohort study, aimed to evaluate bisphosphonates as a risk factor of MRONJ
**Mücke 2016**	**[8]** Mücke T, Deppe H, Hein J, Wolff KD, Mitchell DA, Kesting MR, Retz M, Gschwend JE, Thalgott M. Prevention of bisphosphonate-related osteonecrosis of the jaws in patients with prostate cancer treated with zoledronic acid - A prospective study over 6 years. J Craniomaxillofac Surg. 2016; 44(10):1689-1693. [CENTRAL: CN-01287925; DOI: 10.1016/j.jcms.2016. 07.026. EMBASE: 613404601; PubMed: 27555374]	Randomized controlled trial, but 143 participants (56.5%) didn’t receive tooth extraction.
**Pour 2011**	**[9]** Pour L, Fojtik Z, Adam Z, Sandecka V, Perina V, Pokorny P, Krejci M, Zahradova L. Prevention of osteonecrosis of the jaw during zolendronat use in the patients with multiple myeloma-experience from one centre. Osteoporosis International 2011;22(Suppl 4):S630-S631. [DOI: 10.1007/s00198-011-1717-8]	Historical controlled trial, but no tooth extraction in retrospective control group
**Sandhu 2020**	**[10]** Sandhu S, Salous MH, Sankar V, Margalit DN, Villa A. Osteonecrosis of the jaw and dental extractions: A single-center experience. Oral Surgery Oral Medicine Oral Pathology Oral Radiology 2020;130(5):515-521. [DOI: 10.1016/j.oooo.2020.07.001; EMBASE: 632479144; PubMed: 32723683]	Retrospective cohort study, comparing radionecrosis and medication-related osteonecrosis of the jaw
**Shudo 2018**	**[11]** Shudo A, Kishimoto H, Takaoka K, Noguchi K. Long-term oral bisphosphonates delay healing after tooth extraction: a single insti-tutional	Prospective cohort study, comparing

	prospective study. Osteoporosis International 2018;29(10): 2315-2321. [DOI: 10.1007/s00198-018-4621-7; EMBASE: 622948 659; PubMed: 29967931]	different durations of bisphosphonate administration
**Zhang 2020**	**[12]** Zhang FY, Liu L, Dong J, Zuo J, Jiao J, Yin J, Lv YL. Prospective study of tooth extraction socket healing in osteoporosis patients treated with zoledronic acid. Chinese Journal of Geriatric Dentistry 2020;18(5):269-274. [DOI: 10.19749/j.cn.cjgd.1672-2973.2020.05.004]	Prospective cohort study, comparing different doses of bisphosphonate administration

Primary studies excluded due to study design (30 studies with 39 reports)

Study ID	References	Reason for exclusion
**Agrillo 2007**	**[1]** Agrillo A, Sassano P, Rinna C, Priore P, Iannetti G. Ozone therapy in extractive surgery on patients treated with bisphosphonates. Journal of Craniofacial Surgery 2007;18(5):1068-1070. [DOI: 10.1097/SCS.0b013e3181572609; EMBASE: 47524913; PubMed: 17912084]	Case series study of 15 patients
**Campisi 2018**	**[2]** Campisi G, Panzarella V, Di Fede O, Mauceri R. Prevention of the drug-related osteonecrosis of the jaw (ONJ): Preliminary data of the PROMaF protocol. Supportive Care in Cancer 2018;26(Suppl 3):S375. [DOI: 10.1007/s00520-018-4356-1; EMBASE: 623599092]	Case series study of 25 patients
**Capodiferro 2020**	**[3]** Capodiferro S, Tempesta A, Bucci S, Maiorano E, Favia G, Limongelli L. Aminogam Gel Allows Faster Wound Healing after Oral Surgery by Formation of Mature Connective Tissue with Low Vascular Density and Reducing Inflammatory Infiltration. A Retrospective Study on 580 Cases with Histological and Confocal Laser Investigation. Applied Sciences 2020;10(3):1105. [DOI: 10.3390/app10031105]	Case control study
**Chahine 2008**	**[4]** Chahine C, Cheung MS, Head TW, Schwartz S, Glorieux FH, Rauch F. Tooth extraction socket healing in pediatric patients treated with intravenous pamidronate. The Journal of pediatrics 2008;153(5):719-720. [DOI: 10.1016/j.jpeds.2008.05.003; EMBASE: 352637322; PubMed: 18940358]<br>**[5]** Chahine C. Extraction Socket Healing in Pediatric Patients Treated with Intravenous Pamidronate [M.Sc. Dissertation]. McGill University (Canada) 2012. [ISBN: 9780494841563]	Retrospective cohort study, one-armed
**Ferlito 2010**	**[6]** Ferlito S, Liardo C, Puzzo S. Dental extractions in patient treated with intravenous bisphosphonates and risk of osteonecrosis of jaws: presentation of a preventive protocol and case series. Minerva Stomatologica 2010;59(11-12):593-601. [EMBASE: 361870853; PubMed: 21217623]	Case series study of 34 patients
**Geha 2012**	**[7]** Geha H, Peron JM. BRONJ in cancer patients: Early piezosurgery. British Journal of Oral and Maxillofacial Surgery 2012;50(Suppl 1):S10-S11. [DOI: 10.1016/j.bjoms.2012.04.172; EMBASE: 70795059]	Case series study of 6 patients
**Goia 2007**	**[8]** Goia F, Ortega C, Montemurro F, Appendino P, Vormola R, Basano L, Chiarelli A, Aglietta M. Osteonecrosis of the jaw (ONJ) prevention with piezosurgery for teeth extractions during zoledronic acid use: A preliminary experience. Annals of Oncology 2007;18(Suppl 11):xi62. [DOI: 10.1093/annonc/mdm426]	Case series study of 8 patients
**Kamimura 2019**	**[9]** Kamimura M, Taguchi A, Komatsu M, Koiwai H, Ashizawa R, Ichinose A, Takahara K, Uchiyama S, Kato H. Long waiting time before tooth extraction may increase delayed wound healing in elderly Japanese. Osteoporosis International 2019;30(3):621-628. [DOI: 10.1007/s00198-018-4775-3; EMBASE: 625080625; PubMed: 30460382]<br>**[10]** Taguchi A, Kamimura M, Uchiyama S, Kato H. Factors associated with delayed wound healing longer than 8 weeks after tooth extraction in Japanese patients >60 years of age. Journal of Bone and Mineral Research 2018; 33(Suppl 1):274. [DOI: 10.1002/jbmr.3621; EMBASE: 631812084]	Cross-sectional studies, without eligible outcomes
**Lodi 2010**	**[11]** Lodi G, Sardella A, Salis A, Demarosi F, Tarozzi M, Carrassi A. Tooth extraction in patients taking intravenous bisphosphonates: a preventive protocol and case series. Journal of Oral and Maxillofacial Surgery 2010;68(1): 107-110. [DOI: 10.1016/j.joms.2009.07.068; EMBASE: 355772642; PubMed: 20006163]	Prospective cohort study, one-armed
**Matsumoto 2017**	**[12]** Matsumoto A, Sasaki M, Schmelzeisen R, Oyama Y, Mori Y, Voss PJ. Primary wound closure after tooth extraction for prevention of medication-related osteonecrosis of the jaw in patients under denosumab. Clinical Oral Investigations 2017;21(1):127-134. [DOI: 10.1007/s00784-016-1762-y; EMBASE: 620170659; PubMed: 26924135]	Case series study of 19 patients
**Mauceri 2016**	**[13]** Mauceri R, Giancola F, Panzarella V, Tozzo P, Campisi G, Di Fede O. L-PRF application in extraction sockets of bisphosphonate-treated patients: Preliminary results. Oral Diseases 2016;22(Suppl 2):50. [DOI: 10.1111/odi.12560; EMBASE: 612592264]	Case series study of 10 patients
**Merigo 2012**	**[14]** Merigo E, Meleti M, Manfredi M, Fornaini C, Nammour S, Vescovi P. Laser-assisted protocol for dental extractions and prevention of BRONJ in	Case series study of 500 patients

	bisphosphonates therapy patients. Medicina Oral, Patologia Oral y Cirugia Bucal 2012;17(Suppl 1):S26.[DOI: 10.4317/medoral.17643525; EMBASE: 70943656]	
**Ohta 2015**	**[15]** Ohta R, Onda T, Morikawa T, Ogane S, Nomura T, Takano N, Shibahara T. Clinical review of medication-related osteonecrosis of the jaw regarding risk factors for tooth extraction. International Journal of Oral and Maxillofacial Surgery 2015;44(Suppl 1):e270. [DOI: 10.1016/j.ijom.2015.08.265; EMBASE: 72259604]	Case control study
**Parrulli 2015**	**[16]** Parrulli R, Natalini F, Pelliccioni G, Montebugnoli L, Marchetti C. Surgical protocol in patients taking oral or intramuscolar bisphosphonates. Annali di stomatologia 2015;6(Suppl.1 to n.2):9. [PubMed: PMC4794633]	Case series study of 69 patients
**Regev 2008**	**[17]** Regev E, Lustmann J, Nashef R. Atraumatic teeth extraction in bis-phosphonate-treated patients. Journal of Oral and Maxillofacial Surgery 2008;66(6):1157-1161. [DOI: 10.1016/j.joms.2008.01.059; EMBASE: 351671952; PubMed: 18486780]	Case series study of 10 patients
**Şahin 2020**	**[18]** Şahin O, Tatar B, Ekmekcioğlu C, Aliyev T, Odabaşı O. Prevention of medication related osteonecrosis of the jaw after dentoalveolar surgery: An institution's experience. Journal of Clinical and Experimental Dentistry 2020;12(8):e771-e776. [DOI: 10.4317/jced.56837; PubMed: 32913575]	Case series study of 44 patients
**Saia 2010**	**[19]** Saia G, Blandamura S, Bettini G, Tronchet A, Totola A, Bedogni G, Ferronato G, Nocini PF, Bedogni A. Occurrence of bisphosphonate-related osteonecrosis of the jaw after surgical tooth extraction. Journal of Oral and Maxillofacial Surgery 2010;68(4):797-804. [DOI: 10.1016/j.joms.2009.10.026; EMBASE: 358462307; PubMed: 20307765]	Prospective cohort study, one-armed
**Schifter 2006**	**[20]** Schifter M, Yeoh SC, Coleman HG, Cox S, Zoellner H. Dental extractions/oral surgery safely undertaken in patients on bisphosphonate therapy: A prospective trial. Journal of Oral Pathology and Medicine 2006;35(7):435. [DOI: 10.1111/j.1600-0714.2006.00463.x]	Case series study of 10 patients
**Schiodt 2017**	**[21]** Schiodt M, Ottesen C, Madsen S, Nielsen E, Sand L, Gjoedesen C. Risk of osteonecrosis of the jaws after tooth extraction of 270 teeth with alveolectomy and primary surgical closure in 111 patients on antiresorptive treatment. International Journal of Oral and Maxillofacial Surgery 2017;46(Suppl 1):113. [DOI: 10.1016/j.ijom.2017.02.399]	Case series study of 111 patients
**Schiodt 2018**	**[22]** Schiodt M, Vadhan-Raj S, Chambers MS, Nicolatou-Galitis O, Politis C, Coropciuc R, Fedele S, Jandial D, Zhang J, Ma H, Saunders DP. A multicenter case registry study on medication-related osteonecrosis of the jaw in patients with advanced cancer. Supportive Care in Cancer 2018;26(6):1905-1915. [DOI: 10.1007/s00520-017-4003-2; EMBASE: 619929765; PubMed: 29275525]<br>**[23]** NCT01666106. Osteonecrosis of the Jaw (ONJ) Case Registry. https://clinicaltrials.gov/ct2/show/NCT01666106 (First Submitted June 22, 2012). [ICTRP: https://trialsearch.who.int/?TrialID=NCT01666106]<br>**[24]** Saunders D, Vadhan-Raj S, Chambers M, Nicolatou-Galitis O, Politis C, Coropciuc R, Fedele S, Jandial D, Zhang J, Ma H, Schiodt M. Medication-related osteonecrosis of the jaw in advanced cancer patients: A multicenter case registry study. Supportive Care in Cancer 2016;24(Suppl 1):S36. [DOI: 10.1007/s00520-016-3209-z; EMBASE: 616579346]<br>**[25]** Schiodt M, Vadhan-Raj S, Chambers MS, Nicolatou-Galitis O, Politis C, Coropciuc R, Fedele S, Jandial D, Zhang J, Ma H, Saunders D. Multi-center case registry study on medication-related osteonecrosis of the jaw in advanced cancer patients. Journal of Clinical Oncology 2016;34(15 Suppl):e21663. [DOI: 10.1200/JCO.2016.34.15_suppl.e21663; EMBASE: 611755973]	Case series study of 327 patients
**Sekine 2013**	**[26]** Sekine J, Hattori M, Ueno M, Egawa M, Yoshino A, Kanno T, Nariai Y, Yanai C, Ishibashi H. Surgical management of patients receiving bisphosphonates. International Journal of Oral and Maxillofacial Surgery 2013;42(10):1191. [DOI: 10.1016/j.ijom.2013.07.079; EMBASE: 71230387]	Case series study of 666 patients
**Siew 2020**	**[27]** Siew M, Brown ZL, Perez D. Does Platelet-Rich Fibrin Prevent Medication-Related Osteonecrosis of the Jaw? Journal of Oral and Maxillofacial Surgery 2020:78(10 Suppl):e69-e70. [CENTRAL: CN-02230982; DOI: 10.1016/j.joms.2020.07.138; EMBASE: 2007975690]	Retrospective cohort study, one-armed

**Spanou 2020**	**[28]** Spanou A, Nelson K, Ermer MA, Steybe D, Poxleitner P, Voss PJ. Primary wound closure and perioperative antibiotic therapy for prevention of bisphosphonate-related osteonecrosis of the jaw after tooth extraction. Quintessence International 2020;51(3):220-228. [DOI: 10.3290/j.qi.a43949 ; EMBASE: 631345434; PubMed: 32020132]<br>**[29]** Voss PJ, Poxleitner P, Nelson K, Schmelzeisen R, Spanou A. Healing of extraction sites in patients under bisphosphonate: a clinical cohort study. International Journal of Oral and Maxillofacial Surgery 2015;44(Suppl 1):e162. [DOI: 10.1016/j.ijom.2015.08.858; EMBASE: 72259286]	Retrospective cohort study, one-armed
**Taguchi 2015**	**[30]** Taguchi A, Shiraki M, Tsukiyama M, Miyazaki T, Soen S, Ohta H, Nakamura T, Orimo H. Impact of Osteonecrosis of the Jaw on Osteoporosis Treatment in Japan: Results of a Questionnaire-Based Survey by the Adequate Treatment of Osteoporosis (A-TOP) Research Group. Calcified Tissue International 2015;97(6):542-550. [DOI: 10.1007/s00223-015-0045-y; EMBASE: 605363463; PubMed: 26210799]<br>**[31]** Taguchi A, Shiraki M, Tsukiyama M, Miyazaki T, Soen S, Ohta H, Nakamura T, Orimo H. Impact of osteonecrosis of the jaw on osteoporosis treatment in Japan: Results of a questionnaire-based survey by the adequate treatment of osteoporosis (A-TOP) research group. Journal of Bone and Mineral Research 2015;30(Suppl 1):S164. [DOI: 10.1002/jbmr.2763; EMBASE: 620769059]	Cross-sectional study, with not patients but doctors participating
**Tartaroti 2020**	**[32]** Tartaroti NC, Marques MM, Naclério-Homem MDG, Migliorati CA, Zindel Deboni MC. Antimicrobial photodynamic and photobiomodulation adjuvant therapies for prevention and treatment of medication-related osteonecrosis of the jaws: Case series and long-term follow-up. Photodiagnosis and Photodynamic Therapy 2020;29:101651. [DOI: 10.1016/j.pdpdt.2020.101651; EMBASE: 2004928733; PubMed: 31923636]	Prospective cohort study, one-armed
**UMIN000010239**	**[33]** UMIN000010239. Estimation of platelet-rich fibrin on prevention of bisphosphonate-related osteonecrosis of the jaw. https://upload.umin.ac.jp/cgi-open-bin/ctr_e/ctr_view.cgi?recptno=R000011962 (First Submitted April 1, 2013). [CENTRAL: CN-01841489; ICTRP: https://trialsearch.who.int/?TrialID=JPRN-UMIN000010239]	Prospective trial, one-armed
**UMIN000022479**	**[34]** UMIN000022479. Efficacy of platelet rich fibrin (PRF) for the prevention and the treatment of Medication Related Osteonecrosis of the Jaw (MRONJ). https://upload.umin.ac.jp/cgi-open-bin/ctr_e/ctr_view.cgi?recptno=R000025900 (First Submitted June 1, 2016). [CENTRAL: CN-01828272; ICTRP: https://trialsearch.who.int/?TrialID=JPRN-UMIN000022479]	Prospective trial, one-armed
**Vescovi 2013**	**[35]** Vescovi P, Meleti M, Merigo E, Manfredi M, Fornaini C, Guidotti R, Nammour S. Case series of 589 tooth extractions in patients under bisphosphonates therapy. Proposal of a clinical protocol supported by Nd:YAG low-level laser therapy. Medicina Oral, Patologia Oral y Cirugia Bucal 2013;18(4):e680-e685. [DOI: 10.4317/medoral.18812; EMBASE: 369379218; PubMed: 23524436]	Case series study of 217 patients
**Vescovi 2015**	**[36]** Vescovi P, Giovannacci I, Merigo E, Meleti M, Manfredi M, Fornaini C, Nammour S. Tooth extractions in high-risk patients under bisphosphonate therapy and previously affected with osteonecrosis of the jaws: surgical protocol supported by low-level laser therapy. The Journal of Craniofacial Surgery 2015;26(3):696-699. [DOI: 10.1097/SCS.0000000000001665; EMBASE: 607633235; PubMed: 25915674]<br>**[37]** Giovannacci I, Merigo E, Meleti M, Manfredi M, Vescovi P. Tooth extractions in high risk patients for bisphosphonates related osteonecrosis of the jaws. Annali di Stomatologia 2014;5(Suppl. 2):41-42. [PubMed: PMC4377686]<br>**[38]** Giovannacci I, Merigo E, Sarraj A, Simonazzi T, Vescovi P. Tooth extractions in high-risk patients previously treated for osteonecrosis. Protocol supported by low level laser therapy. Annali di Stomatologia 2014;5(Suppl.3 to n.2):28-29. [PubMed: PMC4308971]	Case series study of 36 patients
**Zhukova 2017**	**[39]** Zhukova NA, Drobyshev AY, Lezhnev DA, Yakimenko II, Shipkova TP. Prevention of jaw osteonecrosis after teeth extractions in patients with malignant tumours of various localisation. International Journal of Oral and Maxillofacial Surgery 2017;46(Suppl 1):152. [DOI: 10.1016/j.ijom.2017.02.524; EMBASE: 616680245]	Case series study of 50 patients

Primary studies ongoing or awaiting classification (4 studies with 4 reports)

Study ID	References	Reason for exclusion
**jRCTs071200006**	**[1]** jRCTs071200006. Investigation of the preventive effects of antimicrobial penetrated collargen plug(TERUPLUG) for post-extraction tooth socket for osteonecrosis of the jaw after tooth extraction in patients using high-dose antiresorptive agent. https://jrct.niph.go.jp/en-latest-detail/jRCTs071200006 (First Submitted April 20, 2020). [CENTRAL: CN-02172521; ICTRP: https://trialsearch.who.int/?TrialID=JPRN-jRCTs071200006]	Ongoing study
**NCT01526915**	**[2]** NCT01526915. Assessment of Platelet Rich Fibrin Efficiency on Healing Delay and on Jawbone Osteochemonecrosis Provoked by Bisphosphonates (OCN/PRF). https://clinicaltrials.gov/ct2/show/NCT01526915 (First Submitted January 31, 2012). [CENTRAL: CN-01591217; ICTRP: https://trialsearch.who.int/?TrialID=NCT01526915]	Study without reported results, awaiting classification
**NCT02198001**	**[3]** NCT02198001. Prospective Randomized Study: Assessment of PRF Efficacy in Prevention of Jaw Osteonecrosis After Tooth Extraction (PRF). https://clinicaltrials.gov/ct2/show/study/NCT02198001 (First Submitted July 15, 2014). [CENTRAL: CN-01547472; ICTRP: https://trialsearch.who.int/?TrialID=NCT02198001]	Study without reported results, awaiting classification
**NCT04257721**	**[4]** NCT04257721. Predictive Score For Maxillary Osteonecrosis After Invasive Oral Surgery (PREV-ONM). https://clinicaltrials.gov/ct2/show/NCT04257721 (First Submitted February 4, 2020). [ICTRP: https://trialsearch.who.int/?TrialID=NCT04257721]	Ongoing study

Secondary studies excluded due to focused clinical questions (2 studies with 2 reports)

Study ID	References	Reason for exclusion
**Govaerts 2020**	**[1]** Govaerts D, Piccart F, Ockerman A, Coropciuc R, Politis C, Jacobs R. Adjuvant therapies for MRONJ: A systematic review. Bone. 2020; 141:115676. [DOI: 10.1016/j.bone.2020.115676; PubMed: 33022455]	Systematic review, focusing on not prophylaxis but treatment of MRONJ
**Rollason 2016**	**[2]** Rollason V, Laverrière A, MacDonald LC, Walsh T, Tramèr MR, Vogt-Ferrier NB. Interventions for treating bisphosphonate-related osteonecrosis of the jaw (BRONJ). Cochrane Database Syst Rev. 2016;2(2):CD008455. [DOI: 10.1002/14651858.CD008455.pub2; PubMed: 26919630]	Systematic review, focusing on not prophylaxis but treatment of BRONJ

Analysis (Forest plots)

Comparison 1: Drug holiday versus drug continuation

1.1 Prevalence of delayed healing

Study or Subgroup	Drug holiday Events	Drug holiday Total	Drug continuation Events	Drug continuation Total	Weight	Risk Difference M-H, Random, 95% CI
**1.1.1 4-week follow-up (NRS)**						
Asaka 2017	4	76	5	26	74.8%	-0.14 [-0.30, 0.02]
Mauceri 2020	0	4	0	16	25.2%	0.00 [-0.27, 0.27]
**Subtotal (95% CI)**		**80**		**42**	**100.0%**	**-0.10 [-0.24, 0.03]**
Total events	4		5			
Heterogeneity: Tau² = 0.00; Chi² = 0.79, df = 1 (P = 0.37); I² = 0%						
Test for overall effect: Z = 1.48 (P = 0.14)						

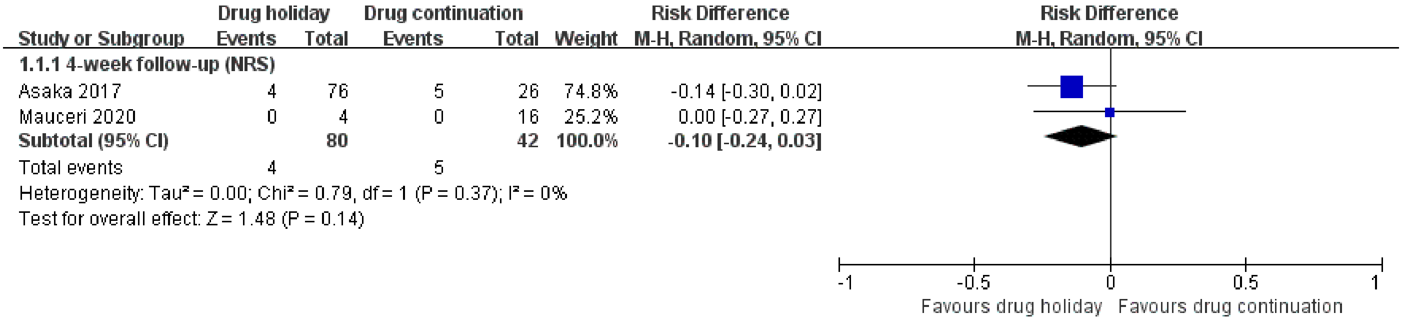

Study or Subgroup	Drug holiday Events	Drug holiday Total	Drug continuation Events	Drug continuation Total	Weight	Risk Ratio M-H, Random, 95% CI
**1.1.1 4-week follow-up (NRS)**						
Asaka 2017	4	76	5	26	100.0%	0.27 [0.08, 0.94]
Mauceri 2020	0	4	0	16		Not estimable
**Subtotal (95% CI)**		**80**		**42**	**100.0%**	**0.27 [0.08, 0.94]**
Total events	4		5			
Heterogeneity: Not applicable						
Test for overall effect: Z = 2.05 (P = 0.04)						

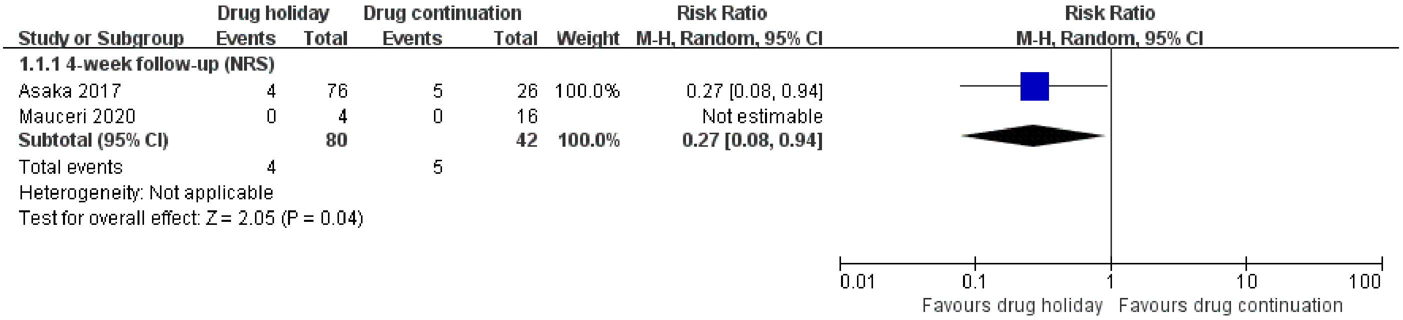

1.2 Incidence proportion of MRONJ

Study or Subgroup	Drug holiday Events	Drug holiday Total	Drug continuation Events	Drug continuation Total	Weight	Risk Difference M-H, Random, 95% CI
**1.2.1 over-3-month follow-up (RCT, high-dose BP & Dmab)**						
Ottesen 2022	4	13	0	10	100.0%	0.31 [0.03, 0.58]
**Subtotal (95% CI)**		**13**		**10**	**100.0%**	**0.31 [0.03, 0.58]**
Total events	4		0			
Heterogeneity: Not applicable						
Test for overall effect: Z = 2.20 (P = 0.03)						
**1.2.2 over-2-month follow-up (NRS, high-dose BP)**						
Bodem 2015	1	17	7	44	62.2%	-0.10 [-0.26, 0.06]
Mauceri 2020	0	4	0	16	19.9%	0.00 [-0.27, 0.27]
Sanchis 2014	6	23	2	11	17.9%	0.08 [-0.21, 0.37]
**Subtotal (95% CI)**		**44**		**71**	**100.0%**	**-0.05 [-0.17, 0.07]**
Total events	7		9			
Heterogeneity: Tau² = 0.00; Chi² = 1.39, df = 2 (P = 0.50); I² = 0%						
Test for overall effect: Z = 0.77 (P = 0.44)						
**1.2.3 over-2-month follow-up (NRS, high-dose BP & Dmab; unit of analysis: tooth extractions)**						
Hasegawa 2021	18	72	21	64	48.0%	-0.08 [-0.23, 0.07]
Hasegawa 2019	17	58	24	105	52.0%	0.06 [-0.08, 0.21]
**Subtotal (95% CI)**		**130**		**169**	**100.0%**	**-0.00 [-0.14, 0.14]**
Total events	35		45			
Heterogeneity: Tau² = 0.00; Chi² = 1.80, df = 1 (P = 0.18); I² = 44%						
Test for overall effect: Z = 0.06 (P = 0.96)						
**1.2.4 over-2-month follow-up (NRS, low-dose BP)**						
Kang 2020	0	286	1	179	93.6%	-0.01 [-0.02, 0.01]
Asaka 2017	0	76	0	26	6.4%	0.00 [-0.05, 0.05]
**Subtotal (95% CI)**		**362**		**205**	**100.0%**	**-0.01 [-0.02, 0.01]**
Total events	0		1			
Heterogeneity: Tau² = 0.00; Chi² = 0.04, df = 1 (P = 0.84); I² = 0%						
Test for overall effect: Z = 0.75 (P = 0.45)						
**1.2.5 over-2-month follow-up (NRS, low-dose BP; unit of analysis: tooth extractions)**						
Hasegawa 2017	27	1818	14	639	100.0%	-0.01 [-0.02, 0.01]
**Subtotal (95% CI)**		**1818**		**639**	**100.0%**	**-0.01 [-0.02, 0.01]**
Total events	27		14			
Heterogeneity: Not applicable						
Test for overall effect: Z = 1.09 (P = 0.27)						

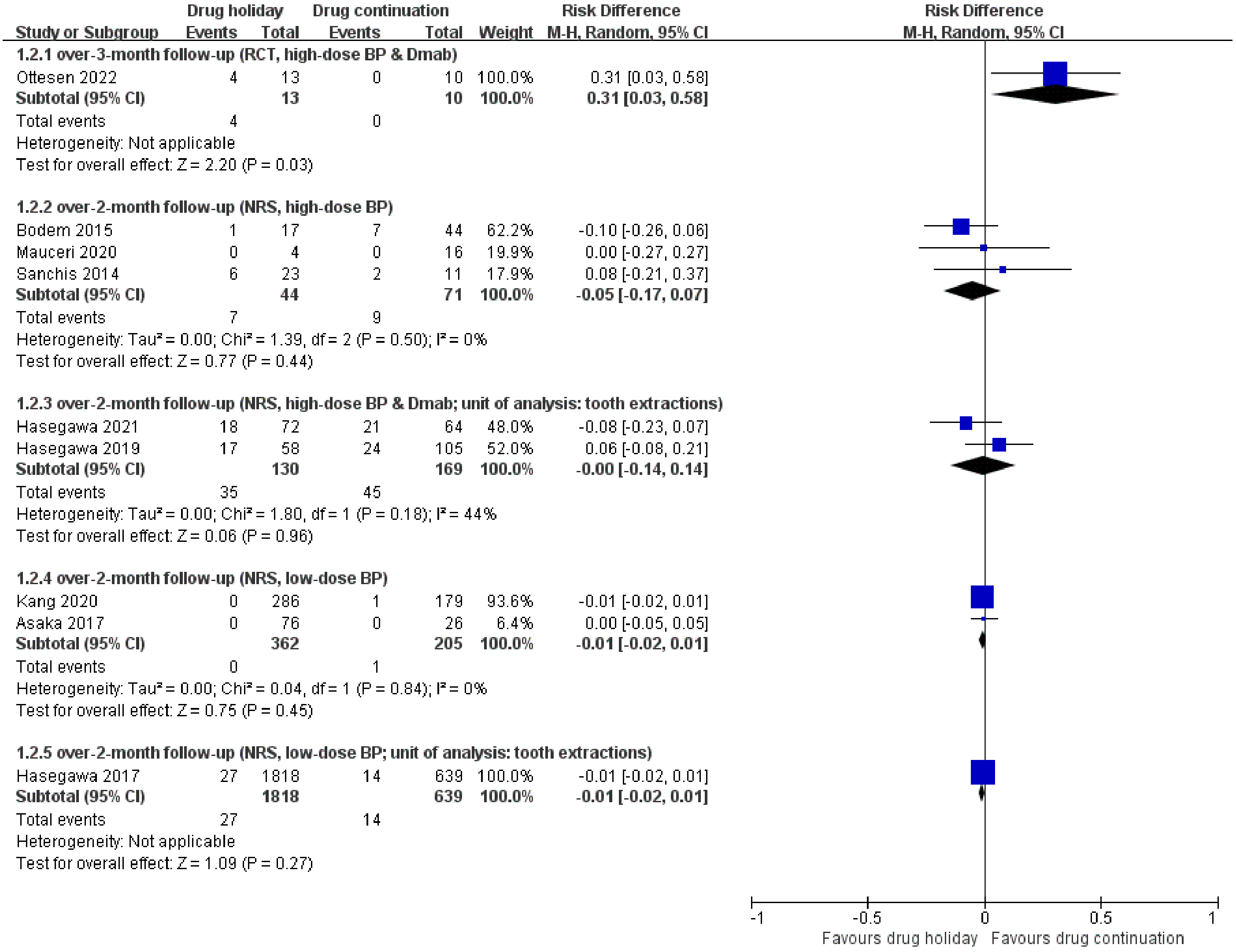

Study or Subgroup	Drug holiday Events	Drug holiday Total	Drug continuation Events	Drug continuation Total	Weight	Risk Ratio M-H, Random, 95% CI
**1.2.1 over-3-month follow-up (RCT, high-dose BP & Dmab)**						
Ottesen 2022	4	13	0	10	100.0%	7.07 [0.42, 117.81]
**Subtotal (95% CI)**		**13**		**10**	**100.0%**	**7.07 [0.42, 117.81]**
Total events	4		0			
Heterogeneity: Not applicable						
Test for overall effect: Z = 1.36 (P = 0.17)						
**1.2.2 over-2-month follow-up (NRS, high-dose BP)**						
Mauceri 2020	0	4	0	16		Not estimable
Bodem 2015	1	17	7	44	36.1%	0.37 [0.05, 2.78]
Sanchis 2014	6	23	2	11	63.9%	1.43 [0.34, 6.00]
**Subtotal (95% CI)**		**44**		**71**	**100.0%**	**0.88 [0.24, 3.23]**
Total events	7		9			
Heterogeneity: Tau² = 0.16; Chi² = 1.20, df = 1 (P = 0.27); I² = 16%						
Test for overall effect: Z = 0.19 (P = 0.85)						
**1.2.3 over-2-month follow-up (NRS, high-dose BP & Dmab; unit of analysis: tooth extractions)**						
Hasegawa 2021	18	72	21	64	50.0%	0.76 [0.45, 1.30]
Hasegawa 2019	17	58	24	105	50.0%	1.28 [0.75, 2.18]
**Subtotal (95% CI)**		**130**		**169**	**100.0%**	**0.99 [0.59, 1.65]**
Total events	35		45			
Heterogeneity: Tau² = 0.06; Chi² = 1.84, df = 1 (P = 0.18); I² = 46%						
Test for overall effect: Z = 0.04 (P = 0.96)						
**1.2.4 over-2-month follow-up (NRS, low-dose BP)**						
Asaka 2017	0	76	0	26		Not estimable
Kang 2020	0	286	1	179	100.0%	0.21 [0.01, 5.10]
**Subtotal (95% CI)**		**362**		**205**	**100.0%**	**0.21 [0.01, 5.10]**
Total events	0		1			
Heterogeneity: Not applicable						
Test for overall effect: Z = 0.96 (P = 0.34)						
**1.2.5 over-2-month follow-up (NRS, low-dose BP; unit of analysis: tooth extractions)**						
Hasegawa 2017	27	1818	14	639	100.0%	0.68 [0.36, 1.28]
**Subtotal (95% CI)**		**1818**		**639**	**100.0%**	**0.68 [0.36, 1.28]**
Total events	27		14			
Heterogeneity: Not applicable						
Test for overall effect: Z = 1.19 (P = 0.23)						

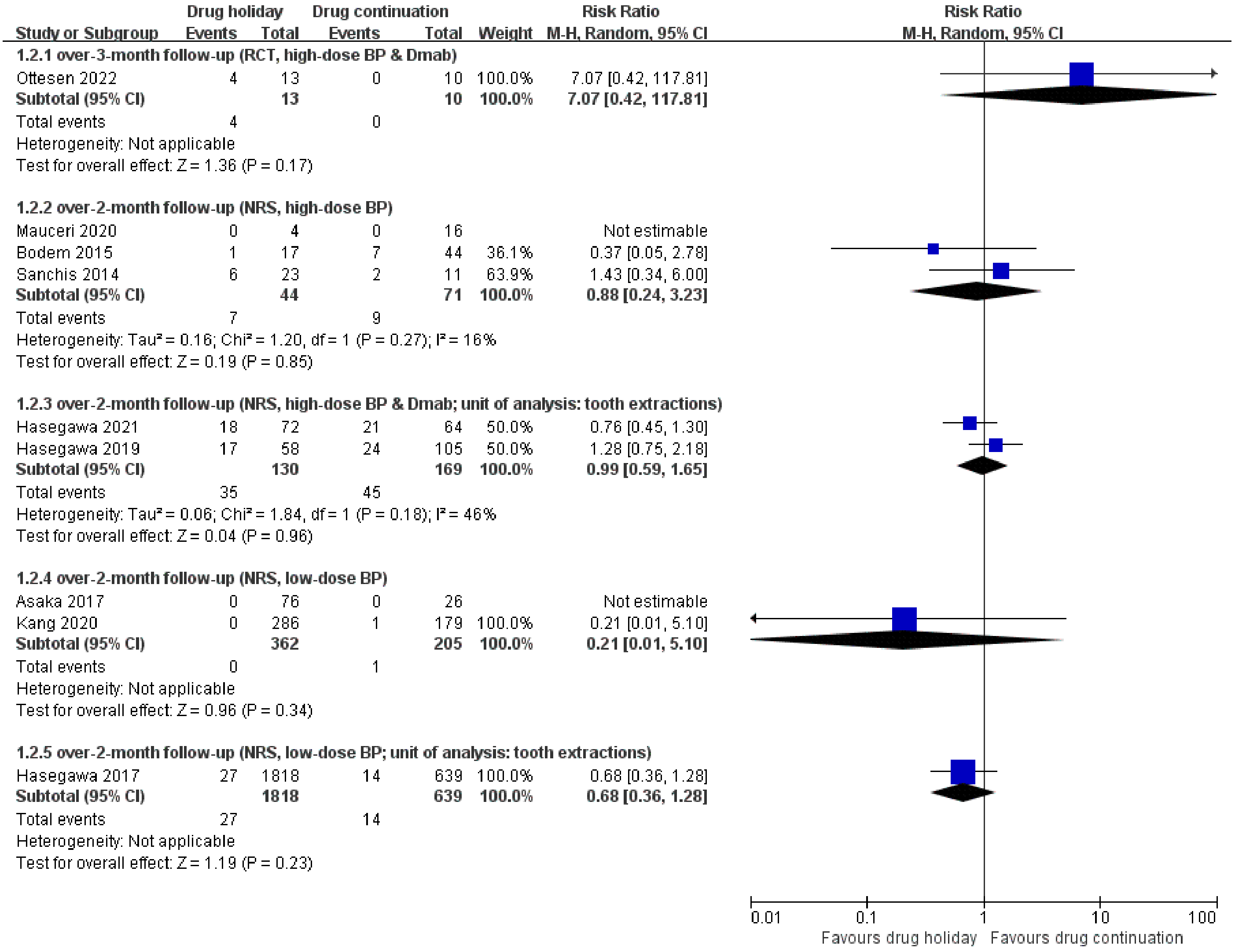

1.3 Mortality (MRONJ-related)

Study or Subgroup	Drug holiday Events	Drug holiday Total	Drug continuation Events	Drug continuation Total	Weight	Risk Difference M-H, Random, 95% CI
**1.3.1 6 month follow-up (RCT)**						
Ottesen 2022	2	13	0	10	100.0%	0.15 [-0.08, 0.39]
**Subtotal (95% CI)**		**13**		**10**	**100.0%**	**0.15 [-0.08, 0.39]**
Total events	2		0			
Heterogeneity: Not applicable						
Test for overall effect: Z = 1.28 (P = 0.20)						

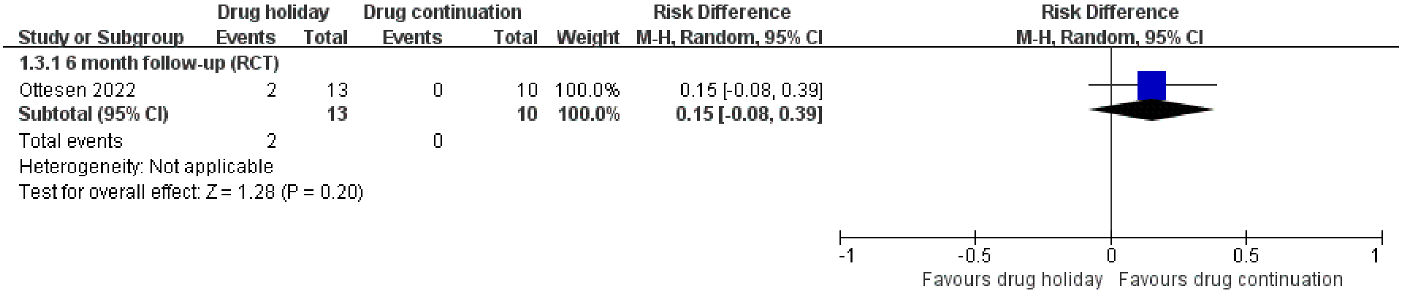

Study or Subgroup	Drug holiday Events	Drug holiday Total	Drug continuation Events	Drug continuation Total	Weight	Risk Ratio M-H, Random, 95% CI
**1.3.1 6 month follow-up (RCT)**						
Ottesen 2022	2	13	0	10	100.0%	3.93 [0.21, 73.71]
**Subtotal (95% CI)**		**13**		**10**	**100.0%**	**3.93 [0.21, 73.71]**
Total events	2		0			
Heterogeneity: Not applicable						
Test for overall effect: Z = 0.91 (P = 0.36)						

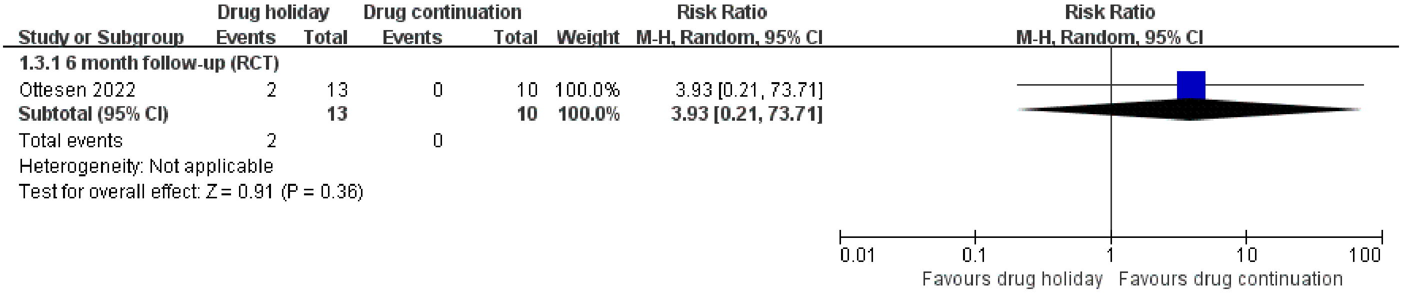

1.4 Mortality (all-cause)

Study or Subgroup	Drug holiday Events	Drug holiday Total	Drug continuation Events	Drug continuation Total	Weight	Risk Difference M-H, Random, 95% CI
**1.4.1 3 month follow-up (RCT)**						
Ottesen 2022	2	13	0	10	100.0%	0.15 [-0.08, 0.39]
**Subtotal (95% CI)**		**13**		**10**	**100.0%**	**0.15 [-0.08, 0.39]**
Total events	2		0			
Heterogeneity: Not applicable						
Test for overall effect: Z = 1.28 (P = 0.20)						
**1.4.2 6 month follow-up (RCT)**						
Ottesen 2022	3	13	0	10	100.0%	0.23 [-0.03, 0.49]
**Subtotal (95% CI)**		**13**		**10**	**100.0%**	**0.23 [-0.03, 0.49]**
Total events	3		0			
Heterogeneity: Not applicable						
Test for overall effect: Z = 1.75 (P = 0.08)						

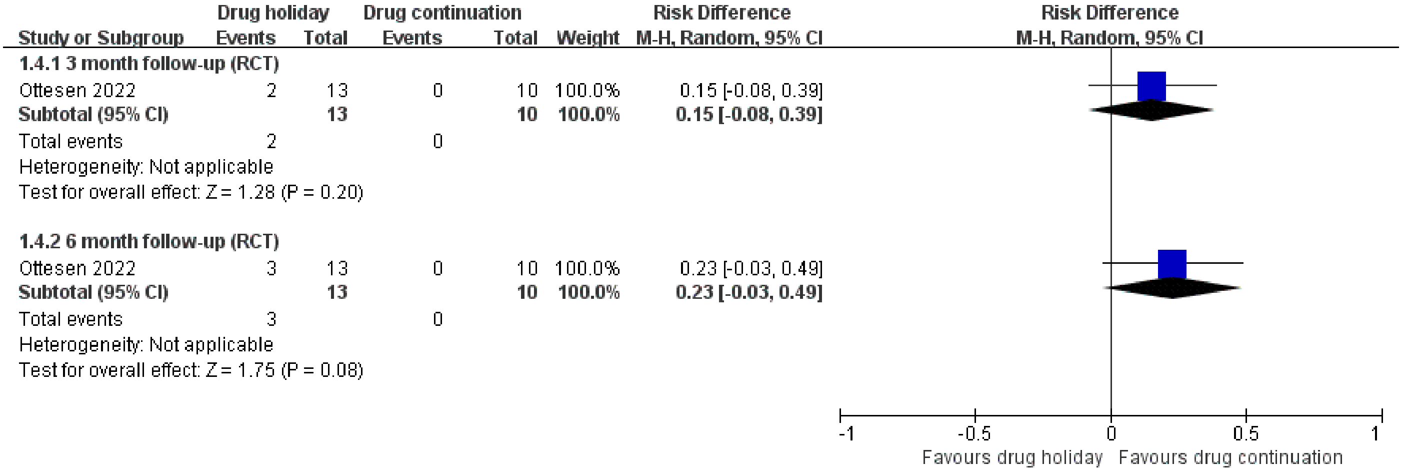

Study or Subgroup	Drug holiday Events	Drug holiday Total	Drug continuation Events	Drug continuation Total	Weight	Risk Ratio M-H, Random, 95% CI
**1.4.1 3 month follow-up (RCT)**						
Ottesen 2022	2	13	0	10	100.0%	3.93 [0.21, 73.71]
**Subtotal (95% CI)**		**13**		**10**	**100.0%**	**3.93 [0.21, 73.71]**
Total events	2		0			
Heterogeneity: Not applicable						
Test for overall effect: Z = 0.91 (P = 0.36)						
**1.4.2 6 month follow-up (RCT)**						
Ottesen 2022	3	13	0	10	100.0%	5.50 [0.32, 95.66]
**Subtotal (95% CI)**		**13**		**10**	**100.0%**	**5.50 [0.32, 95.66]**
Total events	3		0			
Heterogeneity: Not applicable						
Test for overall effect: Z = 1.17 (P = 0.24)						

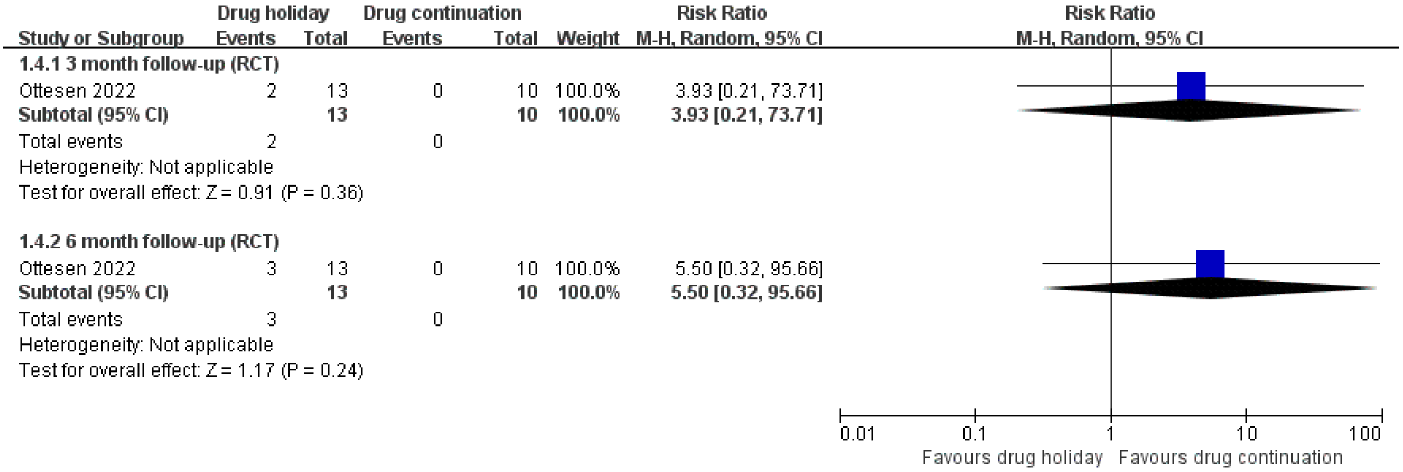

Comparison 2: APC versus control

2.1 PRF versus control

Study or Subgroup	APC Events	APC Total	Control Events	Control Total	Weight	Risk Difference M-H, Random, 95% CI
**2.1.1 Prevalence of delayed healing (2-week follow-up, NRS)**						
Asaka 2017	18	29	55	73	100.0%	-0.13 [-0.34, 0.07]
**Subtotal (95% CI)**		**29**		**73**	**100.0%**	**-0.13 [-0.34, 0.07]**
Total events	18		55			
Heterogeneity: Not applicable						
Test for overall effect: Z = 1.29 (P = 0.20)						
**2.1.2 Prevalence of delayed healing (4-week follow-up, NRS)**						
Asaka 2017	0	29	9	73	100.0%	-0.12 [-0.21, -0.03]
**Subtotal (95% CI)**		**29**		**73**	**100.0%**	**-0.12 [-0.21, -0.03]**
Total events	0		9			
Heterogeneity: Not applicable						
Test for overall effect: Z = 2.72 (P = 0.007)						
**2.1.3 Incidence proportion of MRONJ (over-2-month follow-up, NRS)**						
Asaka 2017	0	29	0	73	100.0%	0.00 [-0.05, 0.05]
**Subtotal (95% CI)**		**29**		**73**	**100.0%**	**0.00 [-0.05, 0.05]**
Total events	0		0			
Heterogeneity: Not applicable						
Test for overall effect: Z = 0.00 (P = 1.00)						
**2.1.4 Incidence proportion of MRONJ (3-month follow-up, RCT)**						
Poxleitner 2020	0	38	0	39	100.0%	0.00 [-0.05, 0.05]
**Subtotal (95% CI)**		**38**		**39**	**100.0%**	**0.00 [-0.05, 0.05]**
Total events	0		0			
Heterogeneity: Not applicable						
Test for overall effect: Z = 0.00 (P = 1.00)						

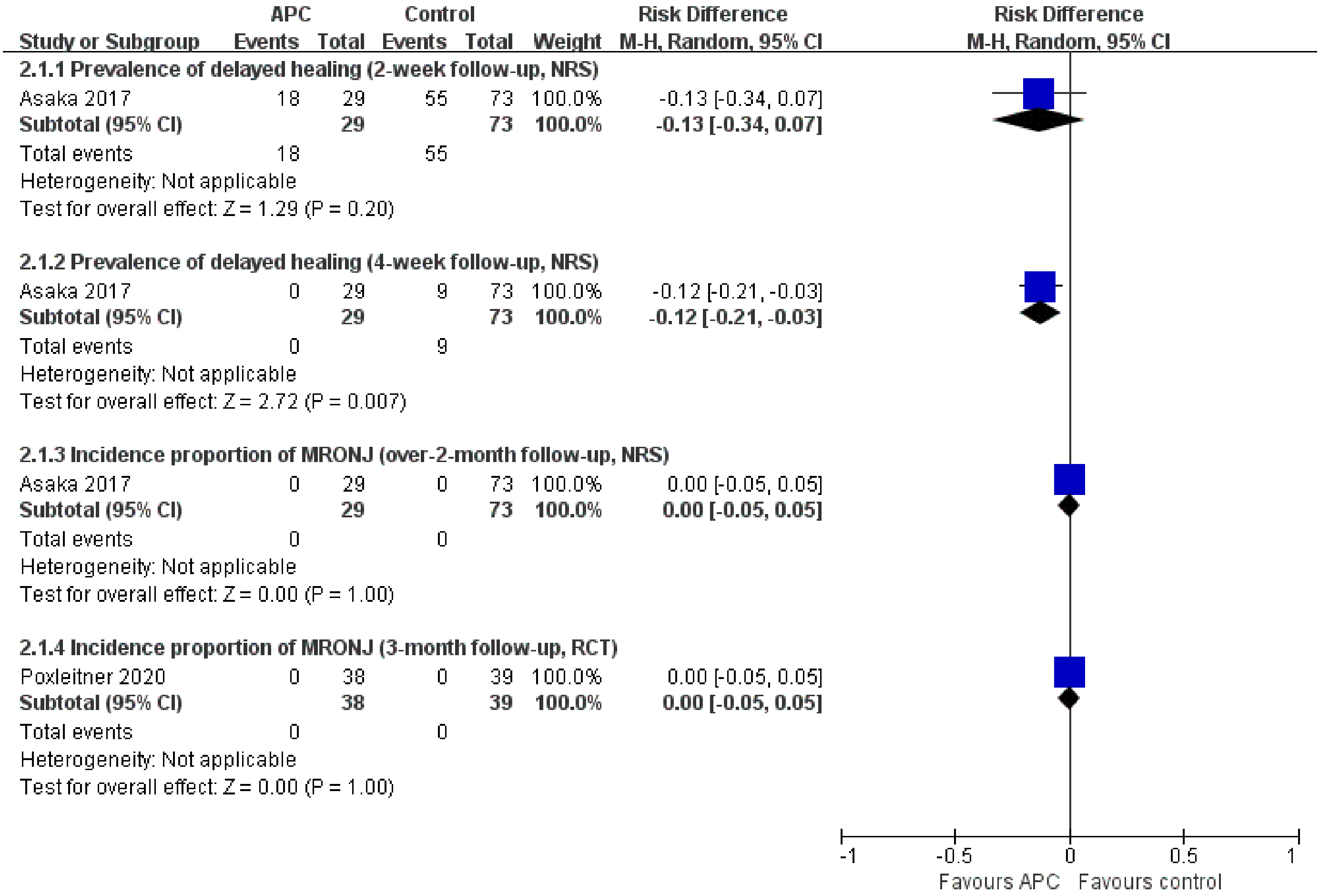

Study or Subgroup	APC Events	APC Total	Control Events	Control Total	Weight	Risk Ratio M-H, Random, 95% CI
**2.1.1 Prevalence of delayed healing (2-week follow-up, NRS)**						
Asaka 2017	18	29	55	73	100.0%	0.82 [0.60, 1.13]
**Subtotal (95% CI)**		**29**		**73**	**100.0%**	**0.82 [0.60, 1.13]**
Total events	18		55			
Heterogeneity: Not applicable						
Test for overall effect: Z = 1.21 (P = 0.23)						
**2.1.2 Prevalence of delayed healing (4-week follow-up, NRS)**						
Asaka 2017	0	29	9	73	100.0%	0.13 [0.01, 2.16]
**Subtotal (95% CI)**		**29**		**73**	**100.0%**	**0.13 [0.01, 2.16]**
Total events	0		9			
Heterogeneity: Not applicable						
Test for overall effect: Z = 1.42 (P = 0.15)						
**2.1.3 Incidence proportion of MRONJ (over-2-month follow-up, NRS)**						
Asaka 2017	0	29	0	73		Not estimable
**Subtotal (95% CI)**		**29**		**73**		**Not estimable**
Total events	0		0			
Heterogeneity: Not applicable						
Test for overall effect: Not applicable						
**2.1.4 Incidence proportion of MRONJ (3-month follow-up, RCT)**						
Poxleitner 2020	0	38	0	39		Not estimable
**Subtotal (95% CI)**		**38**		**39**		**Not estimable**
Total events	0		0			
Heterogeneity: Not applicable						
Test for overall effect: Not applicable						

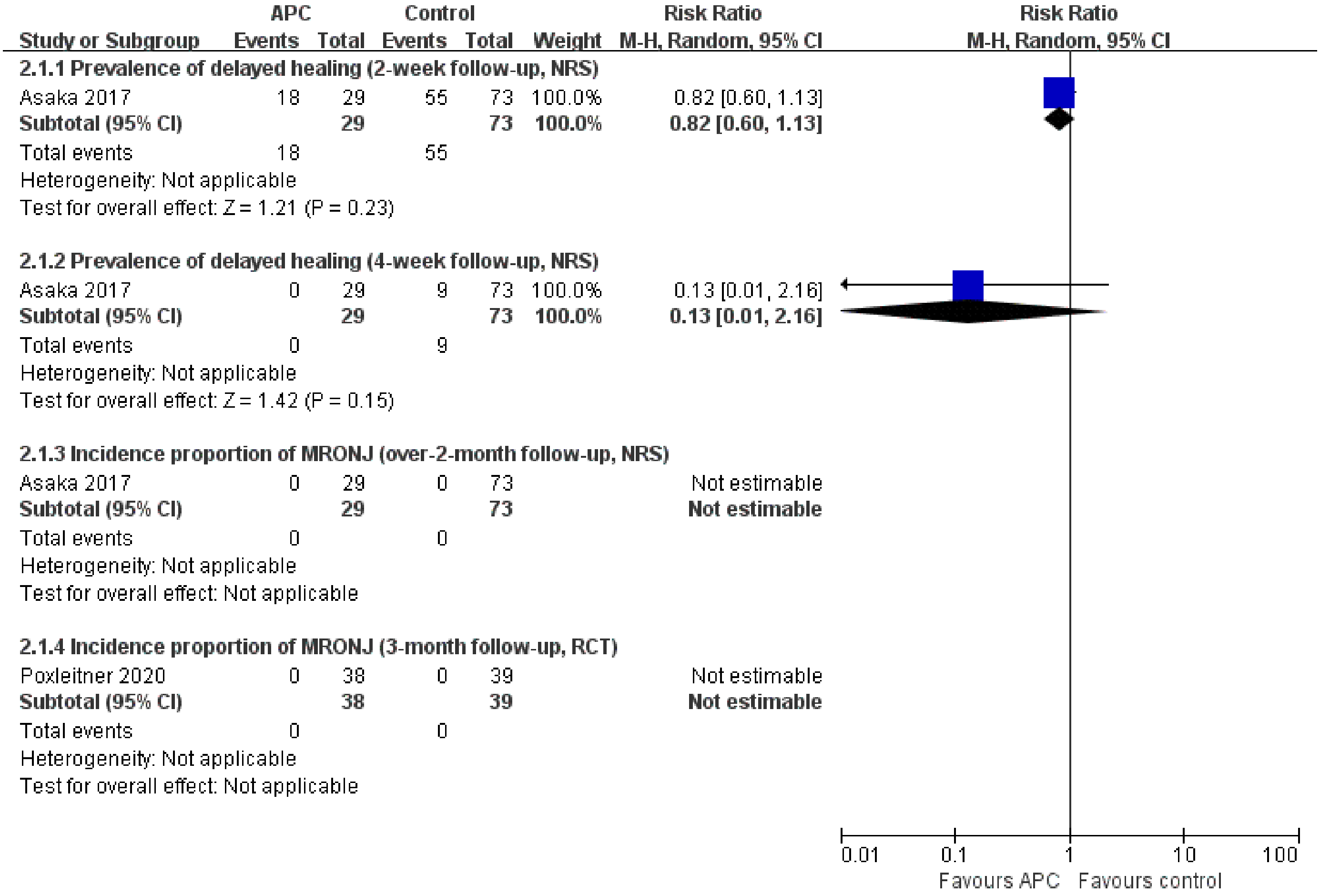

2.2 PRGF versus control

Study or Subgroup	APC Events	APC Total	Control Events	Control Total	Weight	Risk Difference M-H, Random, 95% CI
**2.2.1 Incidence proportion of MRONJ (over 24-month follow-up, RCT)**						
Mozzati 2012	0	91	5	85	100.0%	-0.06 [-0.11, -0.00]
**Subtotal (95% CI)**		**91**		**85**	**100.0%**	**-0.06 [-0.11, -0.00]**
Total events	0		5			
Heterogeneity: Not applicable						
Test for overall effect: Z = 2.14 (P = 0.03)						

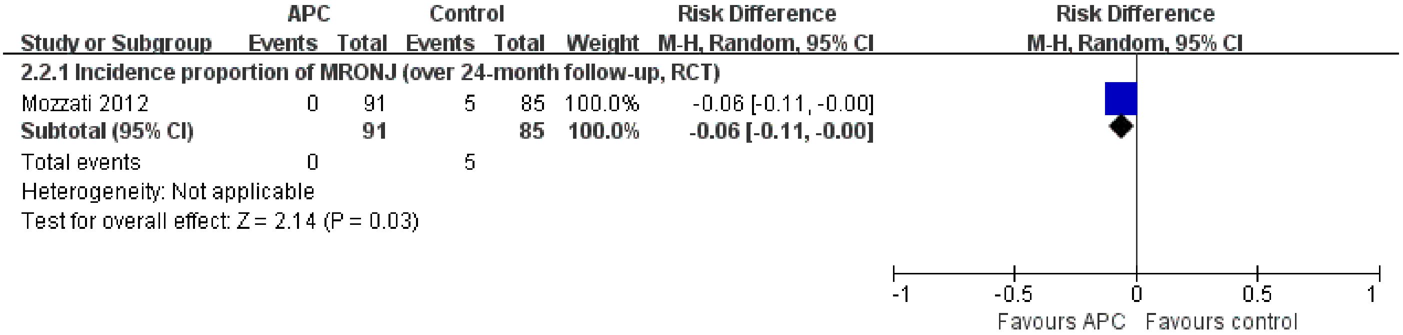

Study or Subgroup	APC Events	APC Total	Control Events	Control Total	Weight	Risk Ratio M-H, Random, 95% CI
**2.2.1 Incidence proportion of MRONJ (over 24-month follow-up, RCT)**						
Mozzati 2012	0	91	5	85	100.0%	0.08 [0.00, 1.51]
**Subtotal (95% CI)**		**91**		**85**	**100.0%**	**0.08 [0.00, 1.51]**
Total events	0		5			
Heterogeneity: Not applicable						
Test for overall effect: Z = 1.68 (P = 0.09)						

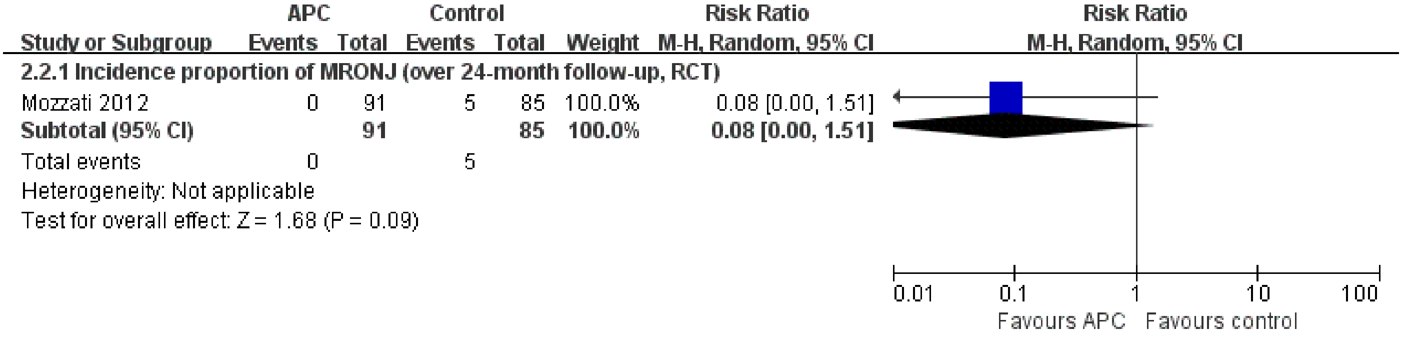

2.3 PRP versus control

Study or Subgroup	APC Events	APC Total	Control Events	Control Total	Weight	Risk Difference M-H, Random, 95% CI
**2.3.1 Incidence proportion of MRONJ (over 12-month follow-up, NRS)**						
Mauceri 2020	0	20	16	905	100.0%	-0.02 [-0.08, 0.05]
**Subtotal (95% CI)**		**20**		**905**	**100.0%**	**-0.02 [-0.08, 0.05]**
Total events	0		16			
Heterogeneity: Not applicable						
Test for overall effect: Z = 0.53 (P = 0.60)						

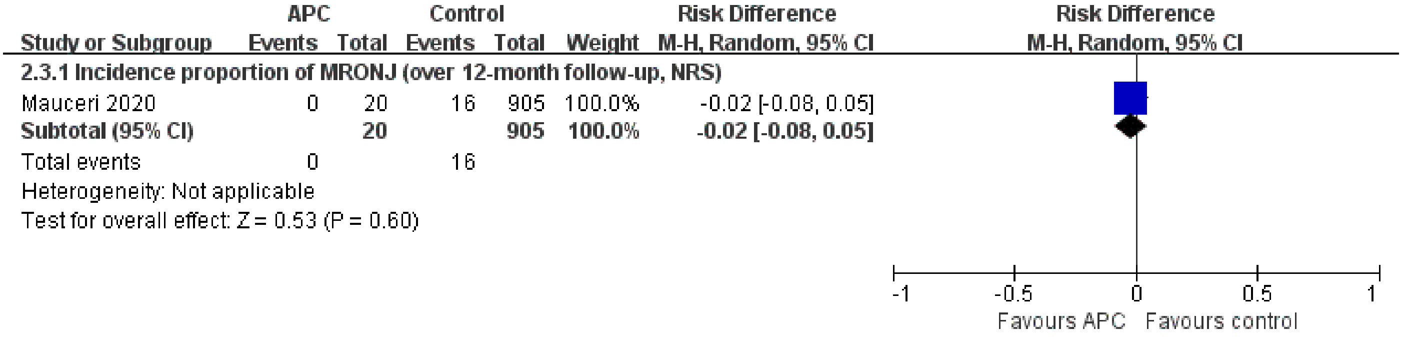

Study or Subgroup	APC Events	APC Total	Control Events	Control Total	Weight	Risk Ratio M-H, Random, 95% CI
**2.3.1 Incidence proportion of MRONJ (over 12-month follow-up, NRS)**						
Mauceri 2020	0	20	16	905	100.0%	1.31 [0.08, 21.07]
**Subtotal (95% CI)**		**20**		**905**	**100.0%**	**1.31 [0.08, 21.07]**
Total events	0		16			
Heterogeneity: Not applicable						
Test for overall effect: Z = 0.19 (P = 0.85)						

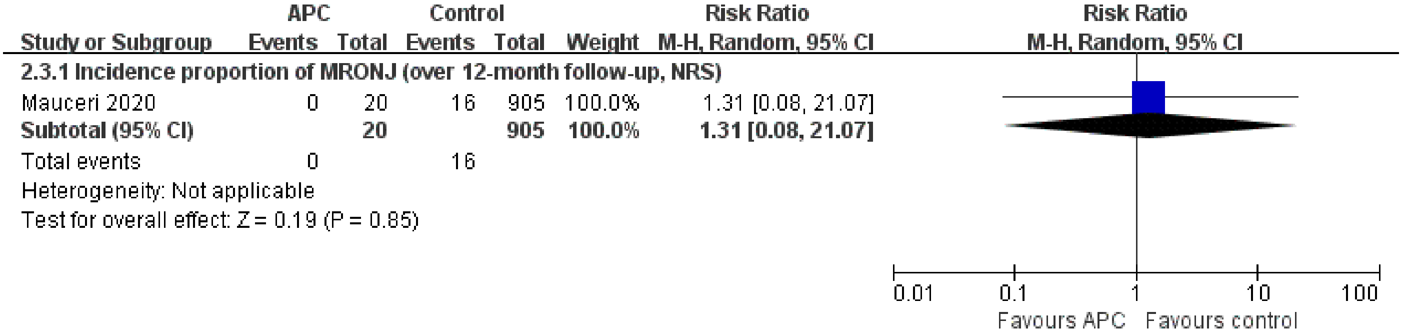

Comparison 3: Different surgical techniques

3.1 Secondary healing versus primary healing

	Secondary healing		Primary healing			Risk Difference
Study or Subgroup	Events	Total	Events	Total	Weight	M-H, Random, 95% CI
**3.1.1 Secondary healing with wound closure: incidence proportion of MRONJ (over 12-month follow-up, RCT)**						
Mozzati 2013	0	334	0	366	100.0%	0.00 [-0.01, 0.01]
**Subtotal (95% CI)**		**334**		**366**	**100.0%**	**0.00 [-0.01, 0.01]**
Total events	0		0			
Heterogeneity: Not applicable						
Test for overall effect: Z = 0.00 (P = 1.00)						
**3.1.2 Secondary healing with wound closure: incidence proportion of MRONJ (over 2-month follow-up, NRS; unit of analysis: tooth extractions)**						
Hasegawa 2017	18	1470	0	105	46.4%	0.01 [-0.00, 0.03]
Hasegawa 2019	22	85	0	2	18.6%	0.26 [-0.17, 0.69]
Hasegawa 2021	20	71	2	15	35.0%	0.15 [-0.05, 0.35]
**Subtotal (95% CI)**		**1626**		**122**	**100.0%**	**0.11 [-0.13, 0.35]**
Total events	60		2			
Heterogeneity: Tau² = 0.03; Chi² = 9.42, df = 2 (P = 0.009); I² = 79%						
Test for overall effect: Z = 0.86 (P = 0.39)						
**3.1.3 Secondary healing with wound open: incidence proportion of MRONJ (over 2-month follow-up, NRS; unit of analysis: tooth extractions)**						
Hasegawa 2017	23	855	0	105	44.3%	0.03 [0.01, 0.04]
Hasegawa 2019	17	57	0	2	20.9%	0.30 [-0.14, 0.74]
Hasegawa 2021	17	50	2	15	34.8%	0.21 [-0.01, 0.42]
**Subtotal (95% CI)**		**962**		**122**	**100.0%**	**0.15 [-0.13, 0.42]**
Total events	57		2			
Heterogeneity: Tau² = 0.04; Chi² = 11.02, df = 2 (P = 0.004); I² = 82%						
Test for overall effect: Z = 1.04 (P = 0.30)						

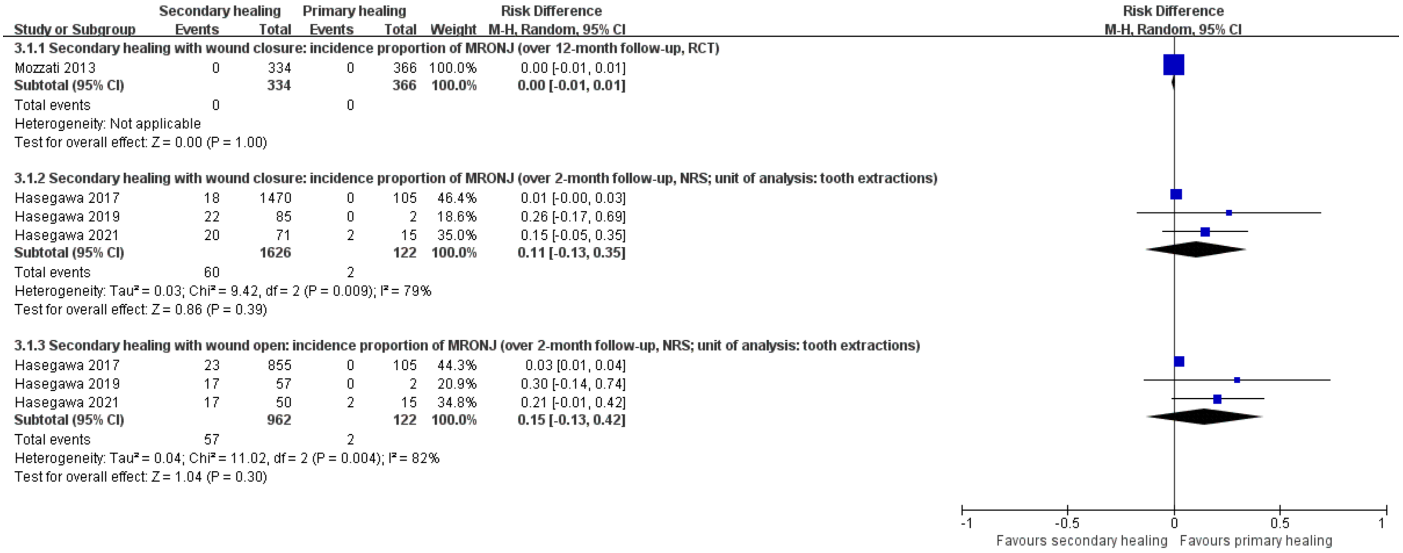

	Secondary healing		Primary healing			Risk Ratio
Study or Subgroup	Events	Total	Events	Total	Weight	M-H, Random, 95% CI
**3.1.1 Secondary healing with wound closure: incidence proportion of MRONJ (over 12-month follow-up, RCT)**						
Mozzati 2013	0	334	0	366		Not estimable
**Subtotal (95% CI)**		**334**		**366**		**Not estimable**
Total events	0		0			
Heterogeneity: Not applicable						
Test for overall effect: Not applicable						
**3.1.2 Secondary healing with wound closure: incidence proportion of MRONJ (over 2-month follow-up, NRS; unit of analysis: tooth extractions)**						
Hasegawa 2017	18	1470	0	105	15.2%	2.67 [0.16, 43.94]
Hasegawa 2019	22	85	0	2	18.3%	1.57 [0.12, 20.21]
Hasegawa 2021	20	71	2	15	66.4%	2.11 [0.55, 8.09]
**Subtotal (95% CI)**		**1626**		**122**	**100.0%**	**2.07 [0.69, 6.19]**
Total events	60		2			
Heterogeneity: Tau² = 0.00; Chi² = 0.08, df = 2 (P = 0.96); I² = 0%						
Test for overall effect: Z = 1.31 (P = 0.19)						
**3.1.3 Secondary healing with wound open: incidence proportion of MRONJ (over 2-month follow-up, NRS; unit of analysis: tooth extractions)**						
Hasegawa 2017	23	855	0	105	15.4%	5.82 [0.36, 95.12]
Hasegawa 2019	17	57	0	2	18.3%	1.81 [0.14, 23.43]
Hasegawa 2021	17	50	2	15	66.3%	2.55 [0.66, 9.80]
**Subtotal (95% CI)**		**962**		**122**	**100.0%**	**2.72 [0.91, 8.14]**
Total events	57		2			
Heterogeneity: Tau² = 0.00; Chi² = 0.42, df = 2 (P = 0.81); I² = 0%						
Test for overall effect: Z = 1.79 (P = 0.07)						

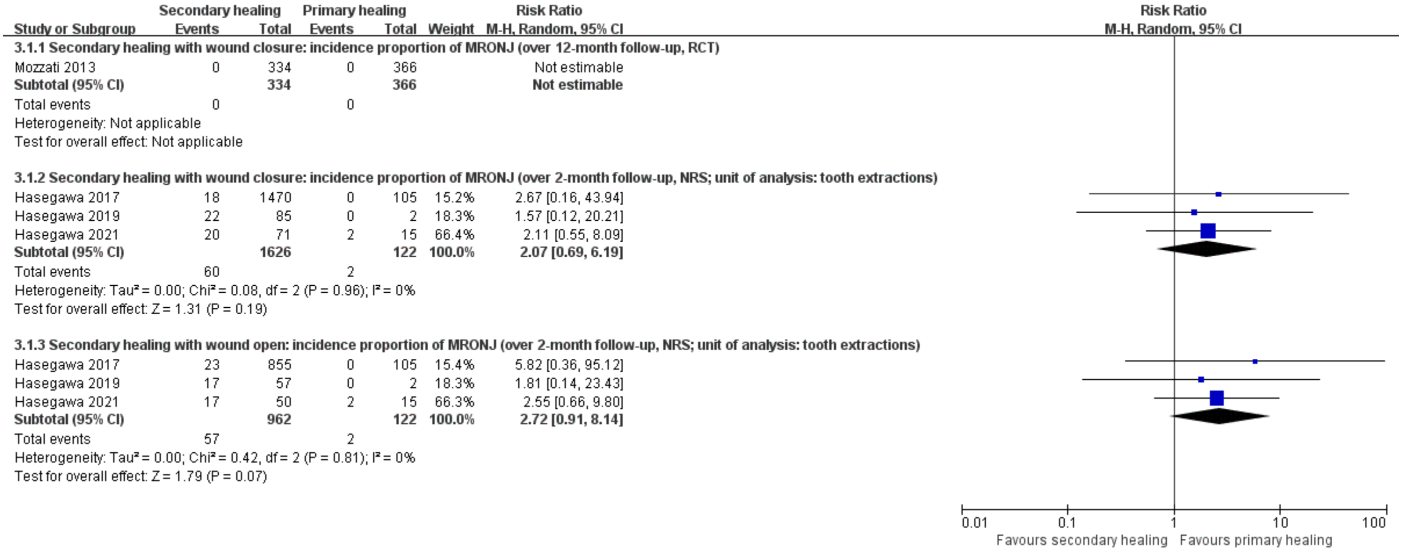

3.2 Primary healing with mucoperiosteal flap versus mucosal flap

	Mucoperiosteal flap		Mucosal flap			Risk Difference
Study or Subgroup	Events	Total	Events	Total	Weight	M-H, Random, 95% CI
**3.2.1 Incidence proportion (cumulative incidence) of MRONJ (2-month follow-up, RCT)**						
Ristow 2021	5	82	11	78	100.0%	-0.08 [-0.17, 0.01]
**Subtotal (95% CI)**		**82**		**78**	**100.0%**	**-0.08 [-0.17, 0.01]**
Total events	5		11			
Heterogeneity: Not applicable						
Test for overall effect: Z = 1.69 (P = 0.09)						
**3.2.2 Mortality (all-cause) (1-month follow-up, RCT)**						
Ristow 2021	2	82	0	78	100.0%	0.02 [-0.02, 0.07]
**Subtotal (95% CI)**		**82**		**78**	**100.0%**	**0.02 [-0.02, 0.07]**
Total events	2		0			
Heterogeneity: Not applicable						
Test for overall effect: Z = 1.17 (P = 0.24)						
**3.2.3 Mortality (all-cause) (4-month follow-up, RCT)**						
Ristow 2021	3	82	1	78	100.0%	0.02 [-0.02, 0.07]
**Subtotal (95% CI)**		**82**		**78**	**100.0%**	**0.02 [-0.02, 0.07]**
Total events	3		1			
Heterogeneity: Not applicable						
Test for overall effect: Z = 0.98 (P = 0.33)						
**3.2.4 Mortality (all-cause) (6-month follow-up, RCT)**						
Ristow 2021	4	82	2	78	100.0%	0.02 [-0.04, 0.08]
**Subtotal (95% CI)**		**82**		**78**	**100.0%**	**0.02 [-0.04, 0.08]**
Total events	4		2			
Heterogeneity: Not applicable						
Test for overall effect: Z = 0.78 (P = 0.44)						

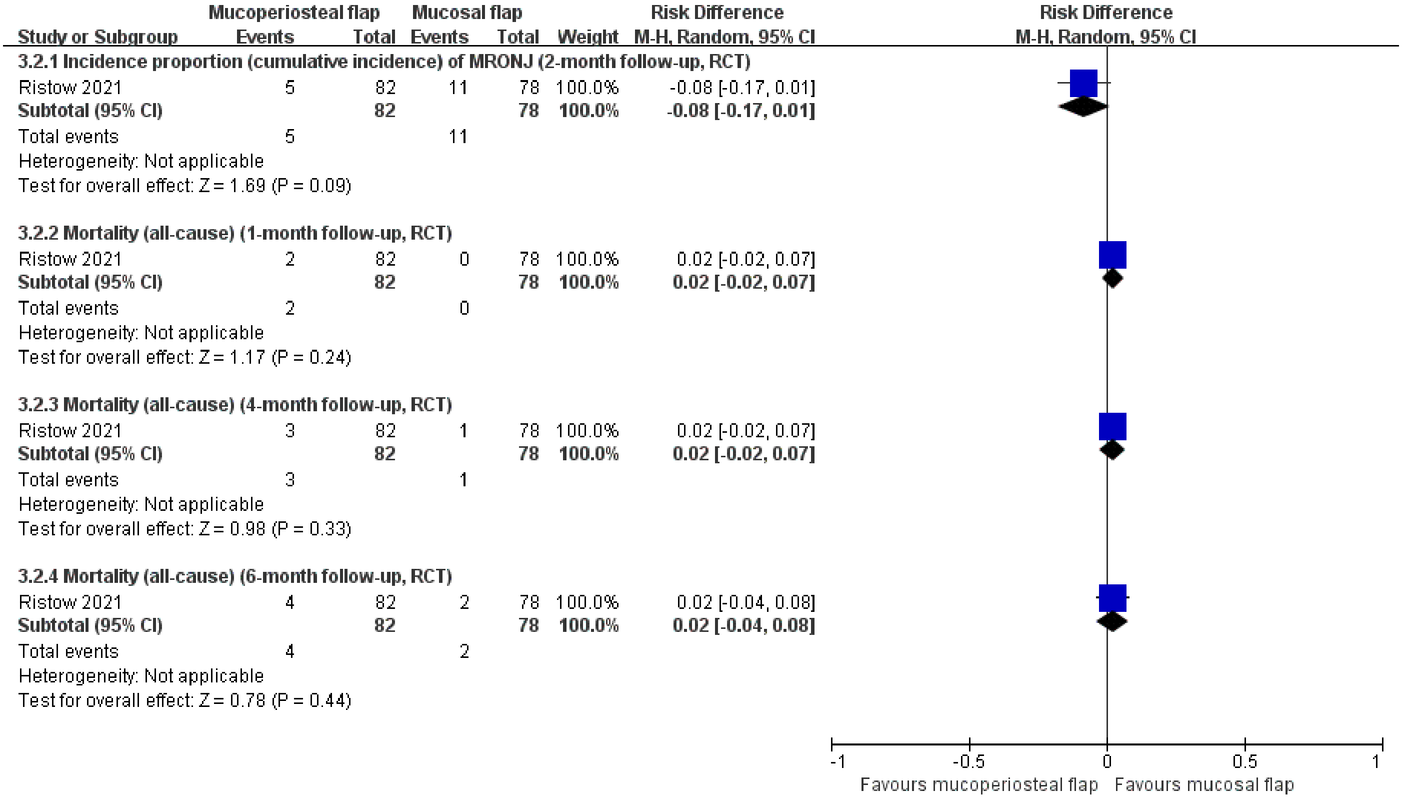

	Mucoperiosteal flap		Mucosal flap			Risk Ratio
Study or Subgroup	Events	Total	Events	Total	Weight	M-H, Random, 95% CI
**3.2.1 Incidence proportion (cumulative incidence) of MRONJ (2-month follow-up, RCT)**						
Ristow 2021	5	82	11	78	100.0%	0.43 [0.16, 1.19]
**Subtotal (95% CI)**		**82**		**78**	**100.0%**	**0.43 [0.16, 1.19]**
Total events	5		11			
Heterogeneity: Not applicable						
Test for overall effect: Z = 1.63 (P = 0.10)						
**3.2.2 Mortality (all-cause) (1-month follow-up, RCT)**						
Ristow 2021	2	82	0	78	100.0%	4.76 [0.23, 97.59]
**Subtotal (95% CI)**		**82**		**78**	**100.0%**	**4.76 [0.23, 97.59]**
Total events	2		0			
Heterogeneity: Not applicable						
Test for overall effect: Z = 1.01 (P = 0.31)						
**3.2.3 Mortality (all-cause) (4-month follow-up, RCT)**						
Ristow 2021	3	82	1	78	100.0%	2.85 [0.30, 26.85]
**Subtotal (95% CI)**		**82**		**78**	**100.0%**	**2.85 [0.30, 26.85]**
Total events	3		1			
Heterogeneity: Not applicable						
Test for overall effect: Z = 0.92 (P = 0.36)						
**3.2.4 Mortality (all-cause) (6-month follow-up, RCT)**						
Ristow 2021	4	82	2	78	100.0%	1.90 [0.36, 10.09]
**Subtotal (95% CI)**		**82**		**78**	**100.0%**	**1.90 [0.36, 10.09]**
Total events	4		2			
Heterogeneity: Not applicable						
Test for overall effect: Z = 0.76 (P = 0.45)						

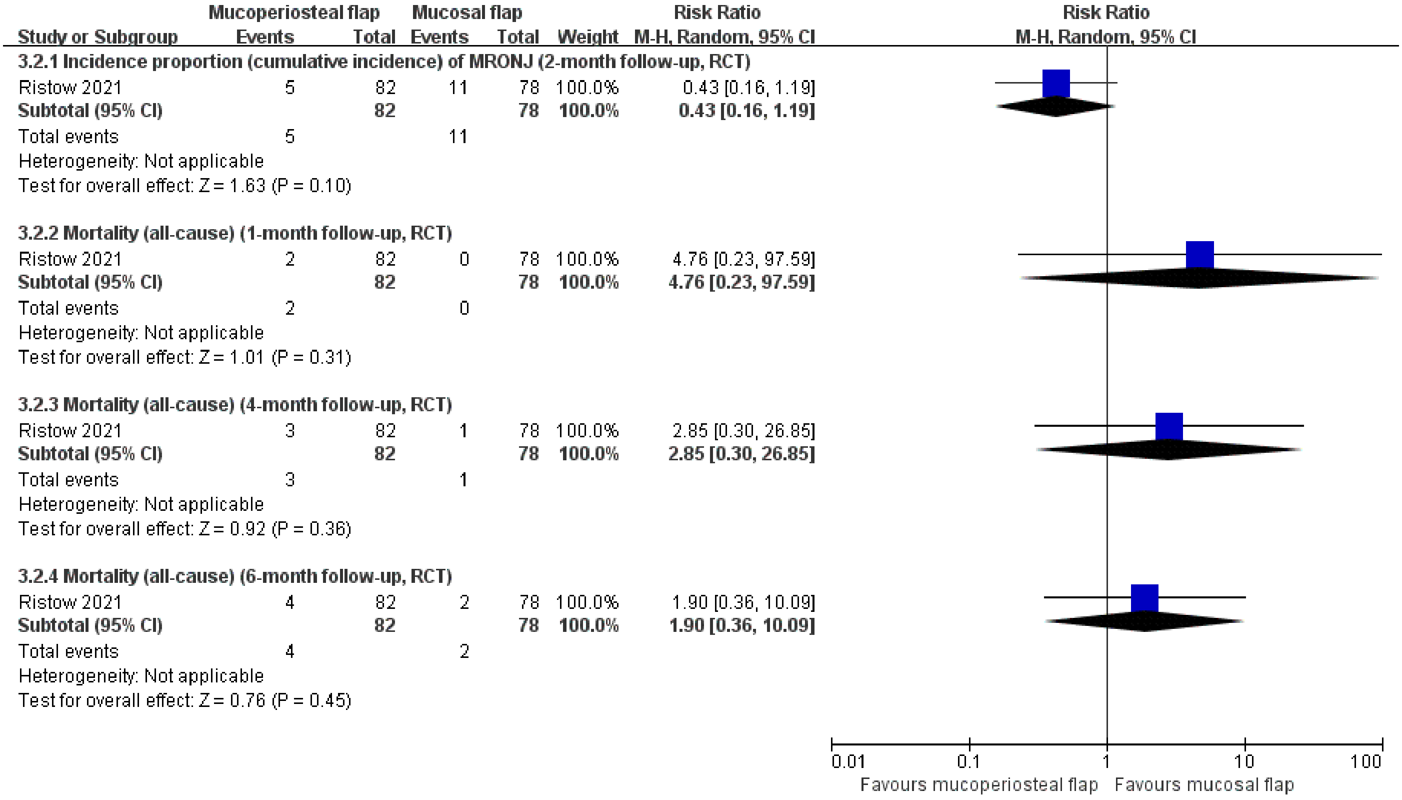

3.3 Secondary healing with mucoperiosteal flap versus no flap

Study or Subgroup	Mucoperiosteal flap Events	Mucoperiosteal flap Total	No flap Events	No flap Total	Weight	Risk Difference M-H, Random, 95% CI
**3.3.1 Incidence proportion of MRONJ (over 4-month follow-up, NRS)**						
Scoletta 2013	5	64	1	63	100.0%	0.06 [-0.01, 0.13]
**Subtotal (95% CI)**		**64**		**63**	**100.0%**	**0.06 [-0.01, 0.13]**
Total events	5		1			
Heterogeneity: Not applicable						
Test for overall effect: Z = 1.68 (P = 0.09)						

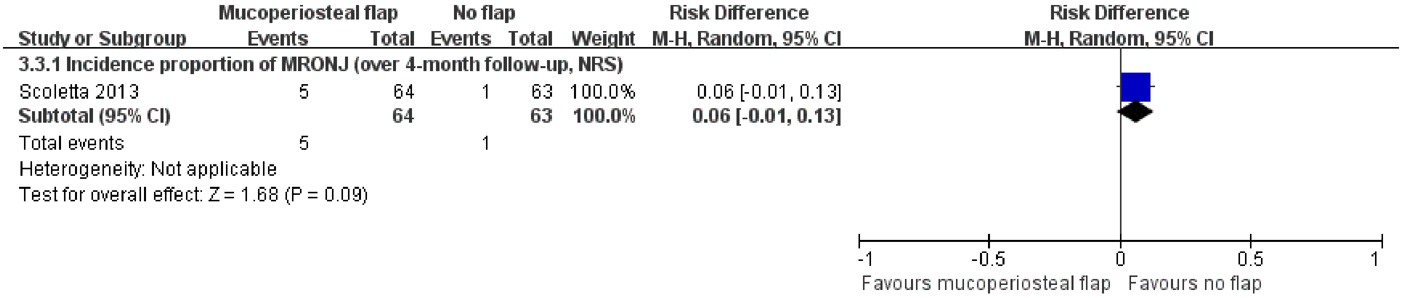

Study or Subgroup	Mucoperiosteal flap Events	Mucoperiosteal flap Total	No flap Events	No flap Total	Weight	Risk Ratio M-H, Random, 95% CI
**3.3.1 Incidence proportion of MRONJ (over 4-month follow-up, NRS)**						
Scoletta 2013	5	64	1	63	100.0%	4.92 [0.59, 40.95]
**Subtotal (95% CI)**		**64**		**63**	**100.0%**	**4.92 [0.59, 40.95]**
Total events	5		1			
Heterogeneity: Not applicable						
Test for overall effect: Z = 1.47 (P = 0.14)						

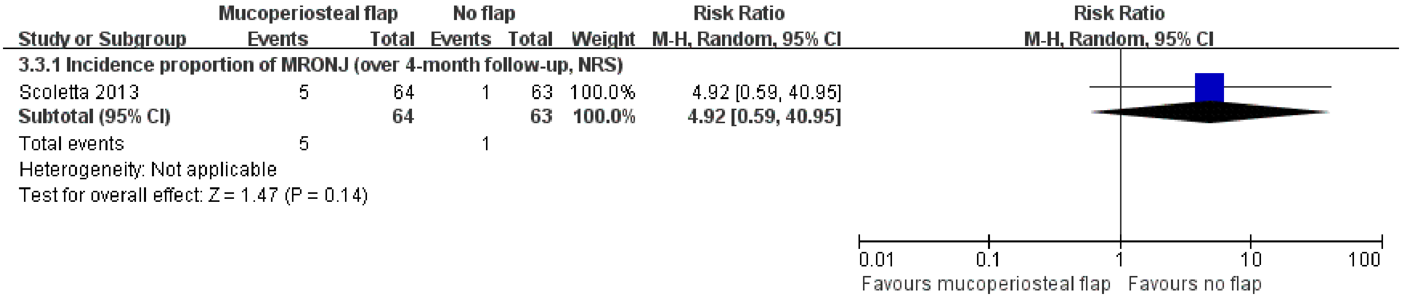

Comparison 4: Antibacterial prophylaxis versus control

4.1 Incidence proportion of MRONJ

Study or Subgroup	Antibacterial prophylaxis Events	Antibacterial prophylaxis Total	Control Events	Control Total	Weight	Risk Difference M-H, Random, 95% CI
**4.1.1 over-2-month follow-up (NRS)**						
Montefusco 2008	0	10	8	14	100.0%	-0.57 [-0.85, -0.29]
**Subtotal (95% CI)**		**10**		**14**	**100.0%**	**-0.57 [-0.85, -0.29]**
Total events	0		8			
Heterogeneity: Not applicable						
Test for overall effect: Z = 4.01 (P < 0.0001)						

Test for subgroup differences: Not applicable

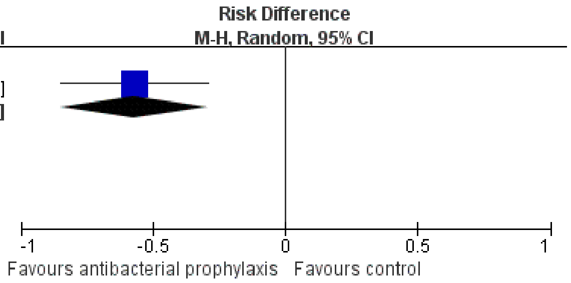

Study or Subgroup	Antibacterial prophylaxis Events	Antibacterial prophylaxis Total	Control Events	Control Total	Weight	Risk Ratio M-H, Random, 95% CI
**4.1.1 over-2-month follow-up (NRS)**						
Montefusco 2008	0	10	8	14	100.0%	0.08 [0.01, 1.25]
**Subtotal (95% CI)**		**10**		**14**	**100.0%**	**0.08 [0.01, 1.25]**
Total events	0		8			
Heterogeneity: Not applicable						
Test for overall effect: Z = 1.80 (P = 0.07)						

Test for subgroup differences: Not applicable

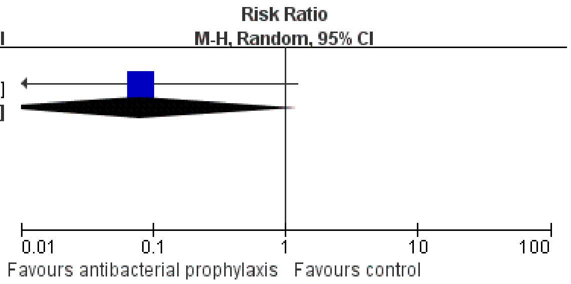

Summary of findings tables (SoF tables)

Comparison	Outcomes	Number of participants (studies)	Certainty of the evidence (GRADE)	Absolute effect/ relative effect (95% CI)
**1 Drug holiday versus drug continuation**				
Intervention: drug holiday before dental extraction<br>Control: drug continuation before dental extraction	**1.1.1** Prevalence of delayed healing<br>follow-up: 4 weeks	122 participants<br>(2 NRSs)	⊕◯◯◯<br>Very low[a,b]	**RD -0.10** (-0.24 to 0.03)<br>**RR 0.27** (0.08 to 0.94)*
	**1.2.1** Incidence proportion of MRONJ<br>follow-up: 3-6 months	23 participants<br>(1 RCT)	⊕⊕◯◯<br>Low[b]	**RD 0.31** (0.03 to 0.58)*<br>**RR 7.07** (0.42 to 117.81)
	**1.2.2** Incidence proportion of MRONJ<br>follow-up: over 2 months	115 participants<br>(3 NRSs)	⊕◯◯◯<br>Very low[a,b]	**RD -0.05** (-0.17 to 0.07)<br>**RR 0.88** (0.24 to 3.23)
	**1.2.3** Incidence proportion of MRONJ<br>follow-up: over 2 months	299 extractions<br>(2 NRSs)	⊕◯◯◯<br>Very low[a,b]	**RD -0.00** (-0.14 to 0.14)<br>**RR 0.99** (0.59 to 1.65)
	**1.2.4** Incidence proportion of MRONJ<br>follow-up: over 2 months	567 participants<br>(2 NRSs)	⊕◯◯◯<br>Very low[a,b]	**RD -0.01** (-0.02 to 0.01)<br>**RR 0.21** (0.01 to 5.10)
	**1.2.5** Incidence proportion of MRONJ<br>follow-up: over 2 months	2457 extractions<br>(1 NRS)	⊕◯◯◯<br>Very low[a,b]	**RD -0.01** (-0.02 to 0.01)<br>**RR 0.68** (0.36 to 1.28)
	**1.3.1** Mortality (MRONJ-related)<br>follow-up: 6 months	23 participants<br>(1 RCT)	⊕⊕◯◯<br>Low[b]	**RD 0.15** (-0.08 to 0.39)<br>**RR 3.93** (0.21 to 73.71)
	**1.4.1** Mortality (all-cause)<br>follow-up: 3 months	23 participants<br>(1 RCT)	⊕⊕◯◯<br>Low[b]	**RR 3.93** (0.21 to 73.71)<br>**RD 0.15** (-0.08 to 0.39)
	**1.4.2** Mortality (all-cause)<br>follow-up: 6 months	23 participants<br>(1 RCT)	⊕⊕◯◯<br>Low[b]	**RR 5.50** (0.32 to 95.66)<br>**RD 0.23** (-0.03 to 0.49)
**2 APC versus control**				
**2.1 PRF versus control**<br>Intervention: dental extraction with PRF<br>Control: dental extraction without PRF	**2.1.1** Prevalence of delayed healing<br>follow-up: 2 weeks	102 participants<br>(1 NRS)	⊕◯◯◯<br>Very low[a,b]	**RD -0.13** (-0.34 to 0.07)<br>**RR 0.82** (0.60 to 1.13)
	**2.1.2** Prevalence of delayed healing<br>follow-up: 4 weeks	102 participants<br>(1 NRS)	⊕◯◯◯<br>Very low[a,b]	**RD -0.12** (-0.21 to -0.03)*<br>**RR 0.13** (0.01 to 2.16)
	**2.1.3** Incidence proportion of MRONJ<br>follow-up: over 2 months	102 participants<br>(1 NRS)	⊕◯◯◯<br>Very low[a,b]	**RD 0.00** (-0.05 to 0.05)<br>**RR** not estimable
	**2.1.4** Incidence proportion of MRONJ<br>follow-up: 3 months	77 participants<br>(1 RCT)	⊕◯◯◯<br>Very low[a,b]	**RD 0.00** (-0.05 to 0.05)<br>**RR** not estimable
**2.2 PRGF versus control**<br>Intervention: with PRGF<br>Control: without PRGF	**2.2.1** Incidence proportion of MRONJ<br>follow-up: over 24 months	176 participants<br>(1 RCT)	⊕◯◯◯<br>Very low[a,b]	**RD -0.06** (-0.11 to -0.00)*<br>**RR 0.08** (0.00 to 1.51)
**2.3 PRP versus control**<br>Intervention: with PRP<br>Control: without PRP	**2.3.1** Incidence proportion of MRONJ<br>follow-up: over 12 months	925 participants<br>(1 NRS)	⊕◯◯◯<br>Very low[a,b]	**RD -0.02** (-0.08 to 0.05)<br>**RR 1.31** (0.08 to 21.07)
**3 Different surgical techniques**				
**3.1.1-3.1.2 Secondary healing with wound closure versus primary healing**	**3.1.1** Incidence proportion of MRONJ<br>follow-up: over 12 months	700 participants<br>(1 RCT)	⊕◯◯◯<br>Very low[a,b]	**RD 0.00** (-0.01 to 0.01)<br>**RR** not estimable
	**3.1.2** Incidence proportion of MRONJ<br>follow-up: over 2 months	1748 extractions<br>(3 NRSs)	⊕◯◯◯<br>Very low[a,b]	**RD 0.11** (-0.13 to 0.35)<br>**RR 2.07** (0.69 to 6.19)
**3.1.3 Secondary healing with wound open versus primary healing**	**3.1.3** Incidence proportion of MRONJ<br>follow-up: over 2 months	1748 extractions<br>(3 NRSs)	⊕◯◯◯<br>Very low[a,b]	**RD 0.15** (-0.13 to 0.42)<br>**RR 2.72** (0.91 to 8.14)
**3.2 Primary healing with mucoperiosteal flap versus mucosal flap**	**3.2.1** Incidence proportion of MRONJ<br>follow-up: 2 months	160 participants<br>(1 RCT)	⊕◯◯◯<br>Very low[a,b]	**RD -0.08** (-0.17 to 0.01)<br>**RR 0.43** (0.16 to 1.19)
	**3.2.2** Mortality (all-cause)<br>follow-up: 1 months	160 participants<br>(1 RCT)	⊕◯◯◯<br>Very low[a,b]	**RD 0.02** (-0.02 to 0.07)<br>**RR 4.76** (0.23 to 97.59)

Comparison	Outcomes	Number of participants (studies)	Certainty of the evidence (GRADE)	Absolute effect/ relative effect (95% CI)
	**3.2.3** Mortality (all-cause)<br>follow-up: 4 months	160 participants<br>(1 RCT)	⊕◯◯◯<br>Very low[a,b]	**RD 0.02** (-0.02 to 0.07)<br>**RR 2.85** (0.30 to 26.85)
	**3.2.4** Mortality (all-cause)<br>follow-up: 6 months	160 participants<br>(1 RCT)	⊕◯◯◯<br>Very low[a,b]	**RD 0.02** (-0.04 to 0.08)<br>**RR 1.90** (0.36 to 10.09)
**3.3 Secondary healing with mucoperiosteal flap versus no flap**	**3.3.1** Incidence proportion of MRONJ<br>follow-up: over 2 months	127 participants<br>(1 NRS)	⊕◯◯◯<br>Very low[a,b]	**RD 0.06** (-0.01 to 0.13)<br>**RR 4.92** (0.59 to 40.95)
**4 Antibacterial prophylaxis versus control**				
Intervention: dental extraction with antibacterial prophylaxis<br>Control: dental extraction without antibacterial prophylaxis	**4.1.1** Incidence proportion of MRONJ<br>follow-up: over 2 months	24 participants<br>(1 NRS)	⊕◯◯◯<br>Very low[a,b]	**RD -0.57** (-0.85 to -0.29)*<br>**RR 0.08** (0.01 to 1.25)

Footnotes:

a. Downgraded two levels due to very serious risk of bias (serious or high risk of bias in included studies);

b. Downgraded two levels due to very serious imprecision (small sample sizes);

* $P < 0.05$.

Appendix (Risk of bias assessment tools)

Appendix 1: ROBIS tool (Risk of Bias in Systematic Reviews)

Signaling questions

Domain 1: Study eligibility criteria

1.1 Did the review adhere to predefined objectives and eligibility criteria?

1.2 Were the eligibility criteria appropriate for the review question?

1.3 Were eligibility criteria unambiguous?

1.4 Were all restrictions in eligibility criteria based on study characteristics appropriate (e.g. date, sample size, study quality, outcomes measured)?

1.5 Were any restrictions in eligibility criteria based on sources of information appropriate (e.g. publication status or format, language, availability of data)?

Domain 2: Identification and selection of studies

2.1 Did the search include an appropriate range of databases/electronic sources for published and unpublished reports?

2.2 Were methods additional to database searching used to identify relevant reports?

2.3 Were the terms and structure of the search strategy likely to retrieve as many eligible studies as possible?

2.4 Were restrictions based on date, publication format, or language appropriate?

2.5 Were efforts made to minimize error in selection of studies?

Domain 3: Data collection and study appraisal

3.1 Were efforts made to minimize error in data collection?

3.2 Were sufficient study characteristics available for both review authors and readers to be able to interpret the results?

3.3 Were all relevant study results collected for use in the synthesis?

3.4 Was risk of bias (or methodological quality) formally assessed using appropriate criteria?

3.5 Were efforts made to minimize error in risk of bias assessment?

Domain 4: Synthesis and findings

4.1 Did the synthesis include all studies that it should?

4.2 Were all predefined analyses reported or departures explained?

4.3 Was the synthesis appropriate given the nature and similarity in the research questions, study designs and outcomes across included studies?

4.4 Was between-study variation (heterogeneity) minimal or addressed in the synthesis?

4.5 Were the findings robust, e.g. as demonstrated through funnel plot or sensitivity analyses?

4.6 Were biases in primary studies minimal or addressed in the synthesis?

Risk of bias in the review

A. Did the interpretation of findings address all of the concerns identified in Domains 1 to 4?

B. Was the relevance of identified studies to the review's research question appropriately considered?

C. Did the reviewers avoid emphasizing results on the basis of their statistical significance?

References

Whiting P, Savović J, Higgins JP, Caldwell DM, Reeves BC, Shea B, Davies P, Kleijnen J, Churchill R. ROBIS: A new tool to assess risk of bias in systematic reviews was developed. J Clin Epidemiol, 2016, 69: 225-234. [DOI: 10.1016/j.jclinepi.2015.06.005; PubMed: 26092286]

Appendix 2: RoB 2 tool (Revised Cochrane Risk-of-Bias tool for randomized trials)

Signaling questions

Domain 1: Bias arising from the randomization process

1.1 Was the allocation sequence random?

1.2 Was the allocation sequence concealed until participants were enrolled and assigned to interventions?

1.3 Did baseline differences between intervention groups suggest a problem with the randomization process?

Domain 2: Risk of bias due to deviations from the intended interventions (effect of assignment to intervention)

2.1. Were participants aware of their assigned intervention during the trial?
2.2. Were carers and people delivering the interventions aware of participants' assigned intervention during the trial?
2.3. If Y/PY/NI to 2.1 or 2.2: Were there deviations from the intended intervention that arose because of the trial context?
2.4 If Y/PY to 2.3: Were these deviations likely to have affected the outcome?
2.5. If Y/PY/NI to 2.4: Were these deviations from intended intervention balanced between groups?
2.6 Was an appropriate analysis used to estimate the effect of assignment to intervention?
2.7 If N/PN/NI to 2.6: Was there potential for a substantial impact (on the result) of the failure to analyze participants in the group to which they were randomized?

Domain 3: Bias due to missing outcome data

3.1 Were data for this outcome available for all, or nearly all, participants randomized?
3.2 If N/PN/NI to 3.1: Is there evidence that the result was not biased by missing outcome data?
3.3 If N/PN to 3.2: Could missingness in the outcome depend on its true value?
3.4 If Y/PY/NI to 3.3: Is it likely that missingness in the outcome depended on its true value?

Domain 4: Risk of bias in measurement of the outcome

4.1 Was the method of measuring the outcome inappropriate?
4.2 Could measurement or ascertainment of the outcome have differed between intervention groups?
4.3 If N/PN/NI to 4.1 and 4.2: Were outcome assessors aware of the intervention received by study participants?
4.4 If Y/PY/NI to 4.3: Could assessment of the outcome have been influenced by knowledge of intervention received?
4.5 If Y/PY/NI to 4.4: Is it likely that assessment of the outcome was influenced by knowledge of intervention received?

Domain 5: Risk of bias in selection of the reported result

5.1 Were the data that produced this result analyzed in accordance with a pre-specified analysis plan that was finalized before unblinded outcome data were available for analysis?
Is the numerical result being assessed likely to have been selected, on the basis of the results, from...
5.2. ... multiple eligible outcome measurements (e.g. scales, definitions, time points) within the outcome domain?
5.3 ... multiple eligible analyses of the data?

References

Sterne JAC, Savović J, Page MJ, Elbers RG, Blencowe NS, Boutron I, Cates CJ, Cheng HY, Corbett MS, Eldridge SM, Emberson JR, Hernán MA, Hopewell S, Hróbjartsson A, Junqueira DR, Jüni P, Kirkham JJ, Lasserson T, Li T, McAleenan A, Reeves BC, Shepperd S, Shrier I, Stewart LA, Tilling K, White IR, Whiting PF, Higgins JPT. RoB 2: a revised tool for assessing risk of bias in randomized trials. BMJ, 2019, 366:l4898. [DOI: 10.1136/bmj.l4898; PubMed: 31462531]

Appendix 3: ROBINS-I tool (Risk of Bias in Nonrandomized Studies of Interventions)

Signaling questions

Bias due to confounding

1.1 Is there potential for confounding of the effect of intervention in this study?
If N/PN to 1.1: the study can be considered to be at low risk of bias due to confounding and no further signaling questions need be considered
If Y/PY to 1.1: determine whether there is a need to assess time-varying confounding:
1.2. Was the analysis based on splitting participants' follow up time according to intervention received?
If N/PN, answer questions relating to baseline confounding (1.4 to 1.6)
If Y/PY, go to question 1.3.
1.3. Were intervention discontinuations or switches likely to be related to factors that are prognostic for the outcome?
If N/PN, answer questions relating to baseline confounding (1.4 to 1.6)
If Y/PY, answer questions relating to both baseline and time-varying confounding (1.7 and 1.8)
Questions relating to baseline confounding only
1.4. Did the authors use an appropriate analysis method that controlled for all the important confounding domains?
1.5. If Y/PY to 1.4: Were confounding domains that were controlled for measured validly and reliably by the variables available in this study?

1.6. Did the authors control for any post-intervention variables that could have been affected by the intervention?

Questions relating to baseline and time-varying confounding

1.7. Did the authors use an appropriate analysis method that controlled for all the important confounding domains and for time-varying confounding?

1.8. If Y/PY to 1.7: Were confounding domains that were controlled for measured validly and reliably by the variables available in this study?

Bias in selection of participants into the study

2.1. Was selection of participants into the study (or into the analysis) based on participant characteristics observed after the start of intervention?

If N/PN to 2.1: go to 2.4

2.2. If Y/PY to 2.1: Were the post-intervention variables that influenced selection likely to be associated with intervention?

2.3 If Y/PY to 2.2: Were the post-intervention variables that influenced selection likely to be influenced by the outcome or a cause of the outcome?

2.4. Do start of follow-up and start of intervention coincide for most participants?

2.5. If Y/PY to 2.2 and 2.3, or N/PN to 2.4: Were adjustment techniques used that are likely to correct for the presence of selection biases?

Bias in classification of interventions

3.1 Were intervention groups clearly defined?

3.2 Was the information used to define intervention groups recorded at the start of the intervention?

3.3 Could classification of intervention status have been affected by knowledge of the outcome or risk of the outcome?

Bias due to deviations from intended interventions

If your aim for this study is to assess the effect of assignment to intervention, answer questions 4.1 and 4.2

4.1. Were there deviations from the intended intervention beyond what would be expected in usual practice?

4.2. If Y/PY to 4.1: Were these deviations from intended intervention unbalanced between groups and likely to have affected the outcome?

Bias due to missing data

5.1 Were outcome data available for all, or nearly all, participants?

5.2 Were participants excluded due to missing data on intervention status?

5.3 Were participants excluded due to missing data on other variables needed for the analysis?

5.4 If PN/N to 5.1, or Y/PY to 5.2 or 5.3: Are the proportion of participants and reasons for missing data similar across interventions?

5.5 If PN/N to 5.1, or Y/PY to 5.2 or 5.3: Is there evidence that results were robust to the presence of missing data?

Bias in measurement of outcomes

6.1 Could the outcome measure have been influenced by knowledge of the intervention received?

6.2 Were outcome assessors aware of the intervention received by study participants?

6.3 Were the methods of outcome assessment comparable across intervention groups?

6.4 Were any systematic errors in measurement of the outcome related to intervention received?

Bias in selection of the reported result

Is the reported effect estimate likely to be selected, on the basis of the results, from...

7.1. ... multiple outcome measurements within the outcome domain?

7.2 ... multiple analyses of the intervention-outcome relationship?

7.3 ... different subgroups?

References

Sterne JA, Hernán MA, Reeves BC, Savović J, Berkman ND, Viswanathan M, Henry D, Altman DG, Ansari MT, Boutron I, Carpenter JR, Chan AW, Churchill R, Deeks JJ, Hróbjartsson A, Kirkham J, Jüni P, Loke YK, Pigott TD, Ramsay CR, Regidor D, Rothstein HR, Sandhu L, Santaguida PL, Schünemann HJ, Shea B, Shrier I, Tugwell P, Turner L, Valentine JC, Waddington H, Waters E, Wells GA, Whiting PF, Higgins JP. ROBINS-I: a tool for assessing risk of bias in non-randomised studies of interventions. BMJ, 2016, 355:i4919. [DOI: 10.1136/bmj.i4919; PubMed: 27733354]
